# RGS5 promotes arterial growth during arteriogenesis

**DOI:** 10.15252/emmm.201403864

**Published:** 2014-06-27

**Authors:** Caroline Arnold, Anja Feldner, Larissa Pfisterer, Maren Hödebeck, Kerstin Troidl, Guillem Genové, Thomas Wieland, Markus Hecker, Thomas Korff

**Affiliations:** 1 Division of Cardiovascular Physiology, Institute of Physiology and Pathophysiology, University of HeidelbergHeidelberg, Germany; 2 Department of Pharmacology, Max-Planck-Institute for Heart and Lung ResearchBad Nauheim, Germany; 3 Division of Vascular Biology, Department of Medical Biochemistry and Biophysics, Karolinska InstitutetStockholm, Sweden; 4 Institute of Experimental and Clinical Pharmacology and Toxicology, University of HeidelbergMannheim, Germany

**Keywords:** arteriogenesis, G-protein, remodelling, RGS5, vascular smooth muscle cells

## Abstract

Arteriogenesis—the growth of collateral arterioles—partially compensates for the progressive occlusion of large conductance arteries as it may occur as a consequence of coronary, cerebral or peripheral artery disease. Despite being clinically highly relevant, mechanisms driving this process remain elusive. In this context, our study revealed that abundance of regulator of G-protein signalling 5 (RGS5) is increased in vascular smooth muscle cells (SMCs) of remodelling collateral arterioles. RGS5 terminates G-protein-coupled signalling cascades which control contractile responses of SMCs. Consequently, overexpression of RGS5 blunted Gα_q/11_-mediated mobilization of intracellular calcium, thereby facilitating Gα_12/13_-mediated RhoA signalling which is crucial for arteriogenesis. Knockdown of RGS5 evoked opposite effects and thus strongly impaired collateral growth as evidenced by a blockade of RhoA activation, SMC proliferation and the inability of these cells to acquire an activated phenotype in RGS5-deficient mice after the onset of arteriogenesis. Collectively, these findings establish RGS5 as a novel determinant of arteriogenesis which shifts G-protein signalling from Gα_q/11_-mediated calcium-dependent contraction towards Gα_12/13_-mediated Rho kinase-dependent SMC activation.

**Subject Categories** Vascular Biology & Angiogenesis

## Introduction

In industrialized countries, cardiovascular diseases by far constitute the leading cause of morbidity and death. Arteriosclerosis often leads to progressive occlusion of large conductance arteries causing severe ischaemia in the affected tissues (Meier *et al*, 2012; Schaper, 2012). Given the clinical relevance of this disease, significant efforts have been made to attenuate consequences of tissue ischaemia by stimulating angiogenesis—the growth of new blood vessels from pre-existing ones. However, local blood supply strictly depends on the diameter of arterioles or arteries to which a capillary network is connected. Consequently, adaptive growth of pre-existing collateral arterioles bypassing an occluded conductance artery appears to be a prerequisite to compensate for the consequences of arteriosclerosis. Recently, a meta-analysis revealed that patients who suffer from coronary artery disease but develop a collateral circulation have a 36% reduced risk of mortality (Meier *et al*, 2012; Schaper, 2012) and thus underlines clinical relevance of this vascular remodelling process. It is referred to as arteriogenesis and basically driven by a change in biomechanical forces to which the vessel wall is exposed. Upon occlusion of a conductance artery, blood flow in collateral arterioles increases and elicits the release of nitric oxide (NO) from endothelial cells and subsequent relaxation of vascular smooth muscle cells (SMCs) (Unthank *et al*, 1996). Through the accompanying dilatation of the collateral arterioles, wall stress is increased which in turn promotes proliferation and pro-inflammatory responses of endothelial cells and SMCs (Heil *et al*, 2006; Demicheva *et al*, 2008). As a consequence, these cells express adhesion molecules (e.g. vascular cell adhesion molecule 1, VCAM-1) and release chemokines such as MCP-1 triggering the recruitment of mononuclear leucocytes—a prerequisite for the subsequent structural changes of the vessel wall (Heil & Schaper, 2004; Demicheva *et al*, 2008). In the long run, these processes lead to the enlargement and corkscrew-like appearance of the collaterals and finally to their transformation into arteries which are capable of bypassing the occluded artery.

A rate limiting and therefore crucial step of this remodelling process is the shift of the SMC phenotype from the resting, differentiated to the activated, proliferative state. Recently, it has been shown that balancing of G-protein signalling is crucial for the control of the SMC phenotype and thus vascular remodelling processes (Althoff *et al*, 2012). Yet, the intracellular effects of G-protein-dependent signalling critically depend on the activity of a group of regulatory proteins known as regulators of G-protein signalling (RGS) (Wieland & Mittmann, 2003; Wieland *et al*, 2007). These proteins contain a GTPase-activating protein (GAP) domain which accelerates hydrolysis of GTP upon binding to the corresponding Gα subunit (Berman *et al*, 1996; Ross & Wilkie, 2000). As a consequence, GPCR-induced signalling is effectively suppressed at high expression levels of these proteins and disconnected from the downstream signal transduction cascade.

Among the various RGS proteins, expression of RGS5 seems to be primarily restricted to pericytes and vascular SMCs (Adams *et al*, 2000). It is up-regulated in the developing vasculature (Cho *et al*, 2003) and in pericytes during wound healing or tumour angiogenesis (Berger *et al*, 2005) but down-regulated in SMCs present in advanced atherosclerotic lesions (Li *et al*, 2004). RGS5-deficient mice appear to be hypotensive (Cho *et al*, 2008; Nisancioglu *et al*, 2008) as compared to their wild-type littermates although initial findings with mice made RGS5-deficient by non-genetic strategies had suggested the opposite (Gu *et al*, 2009). Thus, the impact of RGS5 on the function of vascular SMCs remains elusive.

As G-protein signalling appears to be pivotal for vascular remodelling processes (Althoff *et al*, 2012), we hypothesized that RGS5 may similarly affect vascular remodelling during arteriogenesis by modulating G-protein activity. Consequently, we (i) analysed the expression of RGS5 during arteriogenesis, (ii) identified cellular mechanisms linked to a change in RGS5 expression and (iii) investigated the consequences on arteriogenesis evoked by the loss of RGS5.

## Results

### Increased expression of RGS5 in collateral arteriolar SMCs during arteriogenesis

**Figure 1:**
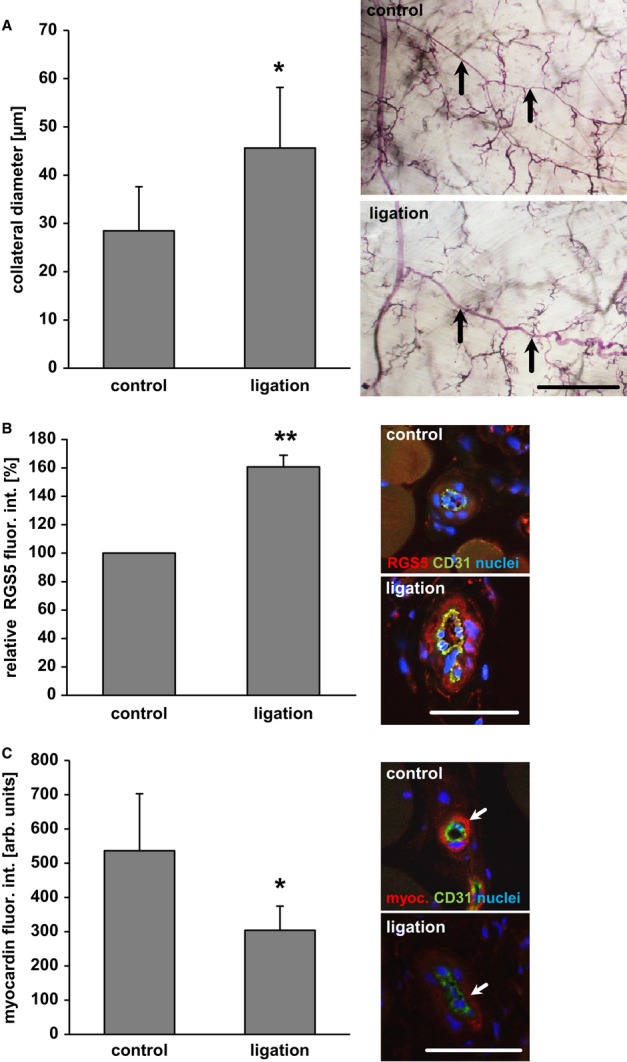
RGS5 protein abundance is increased in remodelling collateral arterioles A   Arteriogenic remodelling of collateral arterioles in the mouse hindlimb was analysed 7 days post ligation of the femoral artery. Growth of the remodelling arterioles (arrows) is significantly increased during this period (*P < 0.05 versus control, *n* = 5; scale bar: 1 mm). B   Quantification of RGS5-specific immunofluorescence intensity (red fluorescence staining) in the SMCs of these arterioles reveals a significant rise in RGS5 abundance over this period (**P < 0.05 versus control, *n* = 5, analysing up to 3 collaterals per animal; scale bar: 50 μm). C   Under these conditions, the fluorescence intensity of myocardin (red), detected in the collateral media, was significantly decreased (*P < 0.05 versus control, *n* = 5; arrows: endothelial cell; CD31 staining: green; scale bar: 50 μm).

Arteriogenesis was induced by ligation of the femoral artery of C57BL/6 wild-type mice stimulating the growth of collateral arterioles in the hindlimb (Fig 1A). Immunofluorescence analyses of the remodelling collaterals 7 days post ligation revealed a marked rise in RGS5 abundance in medial SMCs (Fig 1B). A moderate but significant increase of RGS5 abundance was also observed 3 days upon induction of arteriogenesis (Supplementary Fig S1). Likewise, exemplary microarray analyses of mRNA isolated from growing collaterals in the brain indicated a twofold increase in RGS5 expression (data not shown). To identify whether the change in smooth muscle cell RGS5 abundance during vascular remodelling is associated with a loss of the differentiated phenotype, we additionally examined the expression of myocardin. This transcriptional co-activator maintains the expression of SMC marker proteins and is degraded during biomechanically induced remodelling processes (Pfisterer *et al*, 2012). In line with this, arteriogenesis led to a notable decline of myocardin abundance in the medial SMCs (Fig 1C).

### Nitric oxide and cyclic stretch—critical determinants of arteriogenesis—increase RGS5 protein levels

**Figure 2:**
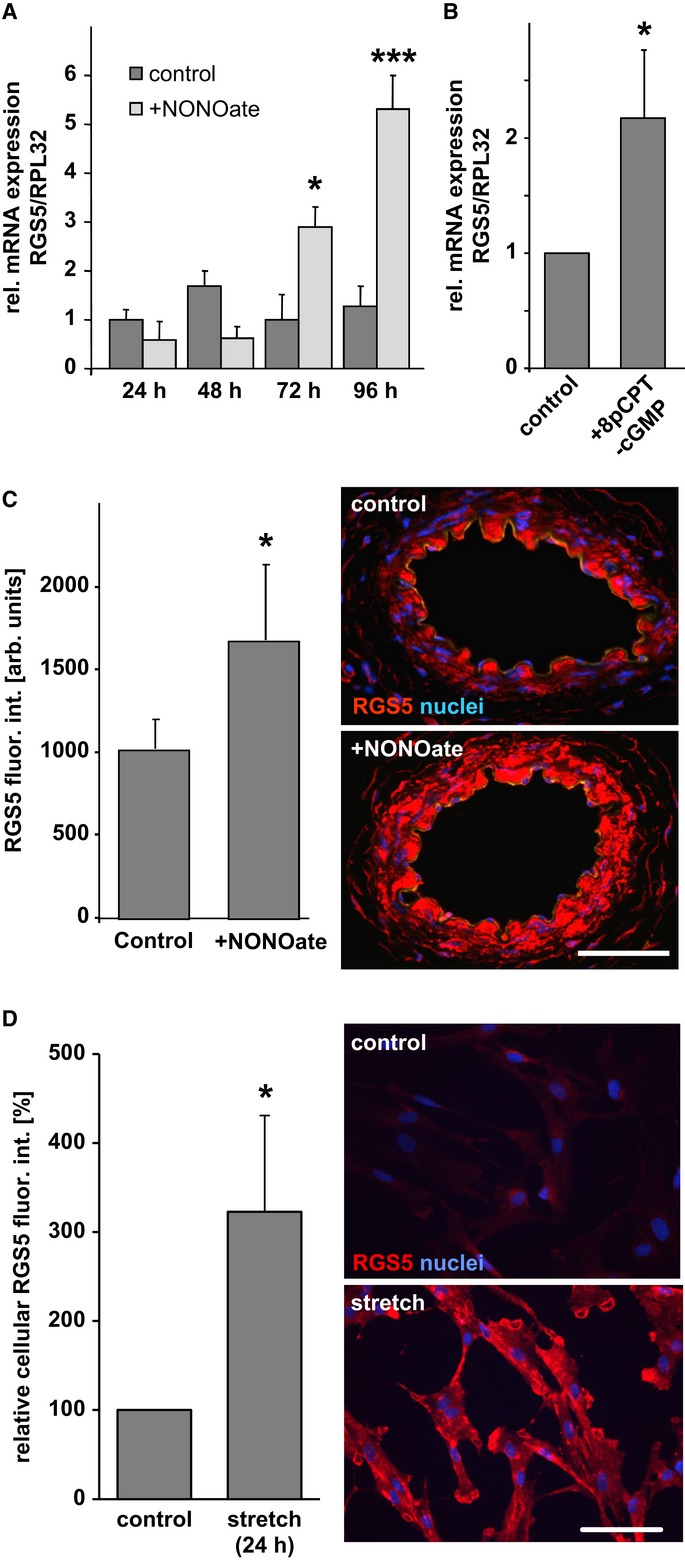
Nitric oxide and cyclic stretch—critical determinants of arteriogenesis—increase RGS5 protein levels A, B   Cultured human umbilical artery SMCs were exposed to the NO donor NONOate (100 μM) or the cell-permeable cyclic GMP analog 8pCPT-cGMP (100 μM) for up to 96 h. Subsequent changes in RGS5 mRNA levels were quantified by real-time PCR analysis (*P < 0.05 versus 0 h; ***P < 0.001 versus 0 h, *n* = 3; expression of the housekeeping gene RPL32 was utilized as internal standard). C   Treatment (72 h) of isolated femoral artery segments with NONOate also reveals a significant rise in RGS5-specific immunofluorescence (red fluorescence staining) in the medial SMCs (*P < 0.05 versus 0 h, *n* = 3, nuclei were counterstained with DAPI (blue fluorescence staining), scale bar: 50 μm). D   Immunofluorescence analyses of control cells cultured under static and stretch-stimulated conditions (0.5 Hz, 0 to 15%) HUASMCs showed an increase in RGS5-specific (red) fluorescence (*P < 0.05 versus control, *n* = 3; RGS5 abundance in the cells was quantified by determining the fluorescence intensity in at least five microscopic fields of view per condition. Control levels were set to 100%; scale bar: 100 μm).

Shear stress-induced release of nitric oxide (NO) from endothelial cells plays an important role in the onset and progression of arteriogenesis (Yu *et al*, 2005; Troidl *et al*, 2009b). Thus, we assumed that continuous exposure of vascular SMCs to NO stimulates RGS5 expression. In fact, exposure of human-cultured SMCs to the NO donor NONOate resulted in a delayed but prominent rise in RGS5 mRNA levels over 96 h (Fig 2A). The same effect was achieved with the cell-permeable cyclic GMP analog 8pCPT-cGMP (Fig 2B), pointing to a role of the soluble guanylyl cyclase/protein kinase G pathway in RGS5 expression downstream of the exposure to NO. Finally, NO-mediated up-regulation of RGS5 expression in the medial SMCs was also confirmed by immunofluorescence analyses of isolated mouse arteries treated with NONOate for up to 72 h (Fig 2C).

Likewise, exposure of human-cultured SMCs to cyclic stretch—a biomechanical stimulus that mimics an increase in wall stress which is another highly relevant determinant of arteriogenesis (Demicheva *et al*, 2008)—also significantly increased RGS5 abundance (Fig 2D).

### RGS5 modulates GPCR agonist-evoked mobilization of intracellular calcium in cultured SMCs

**Figure 3:**
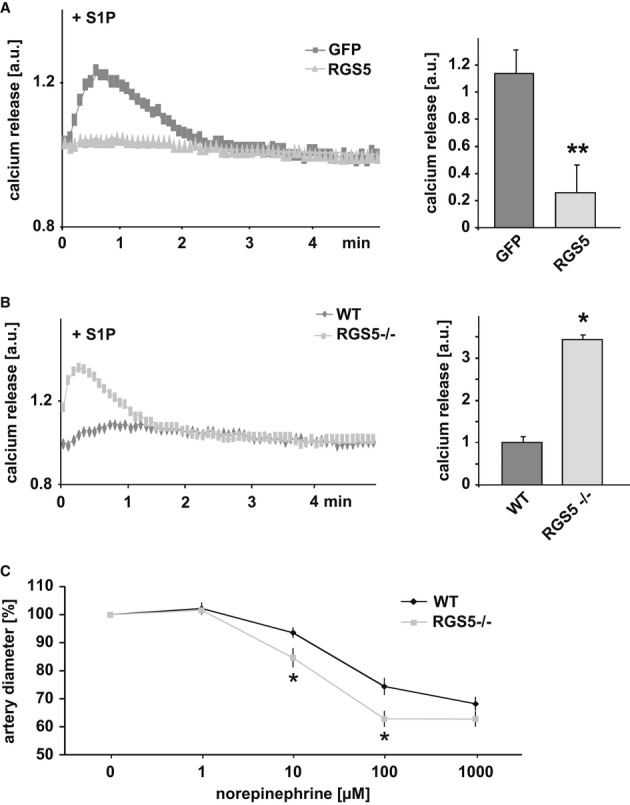
GPCR agonist-induced mobilization of intracellular calcium and arterial constriction is enhanced in RGS5-deficient SMCs A   Cultured human umbilical artery SMCs were transduced with an adenoviral control (GFP) or RGS5 expression vector (RGS5) and then loaded with the calcium-sensing fluorophore Rhod-4 AM. Sphingosine-1-phosphate (S1P, 10 μM) elicits a rapid but transient rise in intracellular calcium in GFP-expressing cells which is virtually abrogated in cells overexpressing RGS5 (**P < 0.01 versus GFP-expressing cells, *n* = 4; calcium transients were quantified by determining the area under the curve). B   Cultured arterial SMCs derived from wild-type (WT) or RGS5-deficient mice (RGS5^−/−^) were loaded with the calcium-sensing fluorophore Rhod-4 AM. S1P moderately increased the intracellular calcium concentration in control SMCs. This effect was significantly reinforced in RGS5-deficient SMCs (*P < 0.05 versus control, *n* = 4). C   Similarly, mesenteric artery segments of RGS5-deficient mice responded with a more pronounced constriction to increasing concentrations of norepinephrine as compared to segments of wild-type (WT) animals (*P < 0.05 versus WT, *n* = 3).

Recently, baseline signalling mediated by the G proteins Gα_q/11_ and Gα_12/13_ has been shown to control phenotype changes of SMCs during vascular remodelling processes (Althoff *et al*, 2012). To investigate possible functional consequences of the rise in RGS5 protein abundance for SMCs during arteriogenesis, G-protein-dependent signalling cascades which are directly or indirectly affected by RGS5 (e.g. Gα_q/11_- and Gα_12/13_-dependent signalling) were analysed. To this end, human-cultured SMCs were transduced with an adenoviral RGS5 or GFP control construct and treated with sphingosine-1-phosphate (S1P). This stimulus activates both Gα_q/11_(calcium)- and Gα_12/13_(RhoA)-dependent signalling pathways and therefore was utilized to assess the general activity of the corresponding G-proteins in the presence of RGS5. Overexpression of RGS5 (adenoviral transduction efficiency up to 70–90%) in fact blunted the Gα_q/11_-dependent increase in intracellular calcium evoked by S1P (Fig 3A), angiotensin II (Supplementary Fig S2A) or the Gα_q/11_-specific agonist bradykinin (Supplementary Fig S3). In agreement, loss of RGS5 reinforced mobilization of intracellular calcium in response to a stimulation with S1P (Fig 3B) or angiotensin II (Supplementary Fig S2B). Correspondingly, isolated mesenteric artery segments from RGS5-deficient mice revealed an enhanced contractile response (calcium-dependent) to increasing concentrations of the Gα_q/11_-specific agonist norepinephrine (Fig 3C).

### Overexpression of RGS5 in cultured SMCs reinforces GPCR agonist-evoked stress fibre formation via the Rho kinase pathway

**Figure 4:**
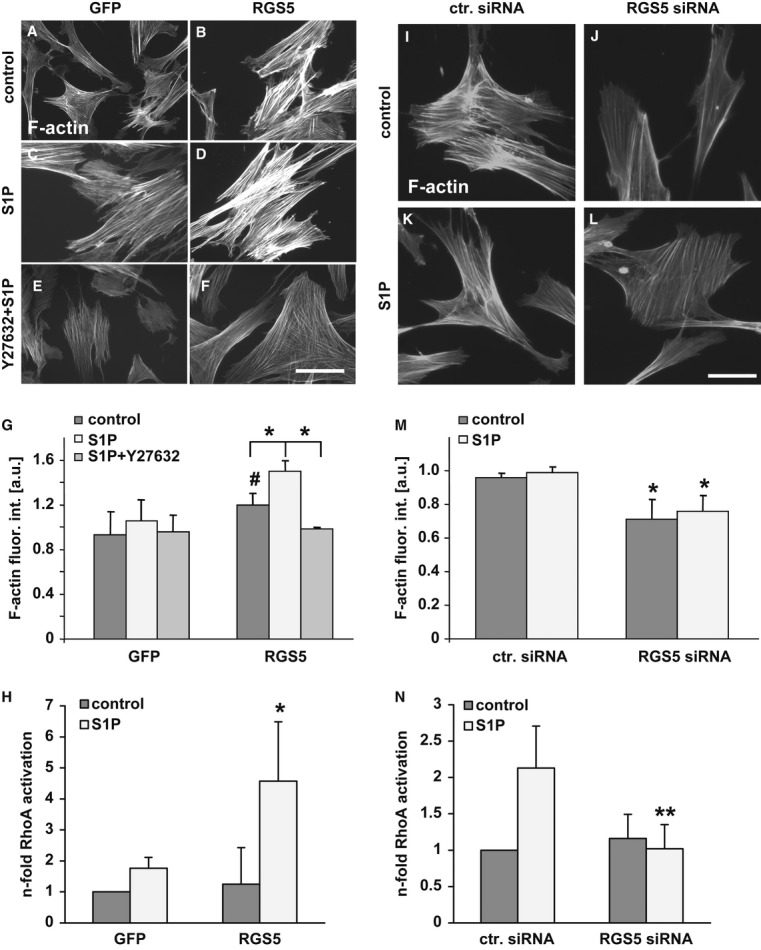
A–G   Cultured human umbilical artery SMCs were transduced with an adenoviral control vector (GFP; A, C, E) or RGS5 expression vector (RGS5; B, D, F). Thereafter, stress fibres (F-actin) were visualized by exposing the cells to TRITC-labelled phalloidin (1:200). RGS5 overexpression alone facilitates stress fibre formation (B and G, ^#^*P* < 0.05 versus GFP-expressing cells, *n* = 3). Stimulation with sphingosine-1-phosphate (C and D, S1P, 10 μM) increases stress fibre formation in cells overexpressing RGS5. This effect is abolished by pretreatment of control and RGS5-overexpressing cells with 5 μM of the Rho-kinase inhibitor Y27632 and subsequent S1P stimulation (E, F and G, *P < 0.05 versus RGS5-overexpressing cells, *n* = 3; cumulative fluorescence intensity of 3–6 whole cells was measured in at least 6 different fields of view, scale bar 20 μm). H   RGS5 overexpression facilitates S1P-stimulated RhoA activation as evidenced by G-LISA-based analyses (*P < 0.05 versus GFP-expressing/S1P-stimulated cells, *n* = 3). I–M   As compared to SMCs transfected with control siRNA (ctr. siRNA; I and K), knockdown of RGS5 significantly decreases stress fibre formation at baseline (RGS5 siRNA; I, J and M) but stimulation with S1P (K, L and M) does not further affect stress fibre formation upon RGS5 knockdown (M, *P < 0.05 versus ctr. siRNA-transfected cells, *n* = 3; cumulative fluorescence intensity of 3–6 whole cells was measured in at least 6 different fields of view, scale bar: 20 μm). N   Loss of RGS5 in HUASMCs inhibits S1P-stimulated RhoA activation as evidenced by G-LISA-based analyses (**P < 0.01 versus ctr. siRNA/S1P-treated cells, *n* = 4).

RGS proteins do not simply abrogate but modulate G-protein signalling in the cell. Turning off Gα_q/11_-mediated mobilization of intracellular calcium by R4 RGS proteins such as RGS5 should not affect the S1P/Gα_12/13_-dependent activation of the Rho kinase pathway in RGS5-overexpressing SMCs. Subsequent experiments analysing actin remodelling as a readout for Rho kinase activity in human-cultured SMCs revealed that RGS5 overexpression already reinforced stress fibre formation in these cells without further stimulation (Fig 4A, B and G). Stimulation with S1P increased stress fibre formation even further (Fig 4C, D and G). The RGS5-dependent stress fibre formation was sensitive to the Rho kinase inhibitor Y27632 (Fig 4E, F and G). Additionally, direct analyses of RhoA activity in SMCs revealed that the S1P-induced formation of RhoA-GTP was much more pronounced in SMCs overexpressing RGS5 (Fig 4H).

In line with this, knockdown of RGS5 expression in human-cultured SMCs through RNA interference (Supplementary Fig S4) diminished the formation of stress fibres at baseline, inhibited their accumulation upon S1P stimulation (Fig 4I–M) and diminished the S1P-dependent activation of RhoA (Fig 4N) without affecting baseline proliferation or apoptosis (Supplementary Figs S5 and S6). Furthermore, enhanced stress fibre formation in S1P-stimulated RGS5-overexpressing SMCs was blocked by RGS-Lsc—a specific inhibitor of Gα_12/13_ signalling (Wuertz *et al*, 2010)—corroborating that the RGS5-dependent RhoA activation occurred via a Gα_12/13_-regulated pathway (Supplementary Fig S7). Correspondingly, the abundance of globular actin is decreased in RGS5-overexpressing SMCs (Supplementary Fig S8). Collectively, these data imply that RGS5 attenuates Gα_q/11_-dependent signalling while promoting Gα_12/13_-mediated RhoA signalling in vascular SMCs. Exemplary profiling of the activation, that is, phosphorylation of a set of kinases after RGS5 overexpression in cultured human SMCs revealed that the activity of well-known RhoA-regulated protein kinases such as p38a (Zhang *et al*, 1995; Dubroca *et al*, 2005), JNK (Teramoto *et al*, 1996), HSP27 (Dubroca *et al*, 2005) and FAK (Zhang *et al*, 2012) were increased twofold, while RhoA-independent kinases such as ERK1/2 (Zuckerbraun *et al*, 2003) were not affected (data not shown).

### RGS5 deficiency impairs the growth of collateral arterioles during arteriogenesis

**Figure 5:**
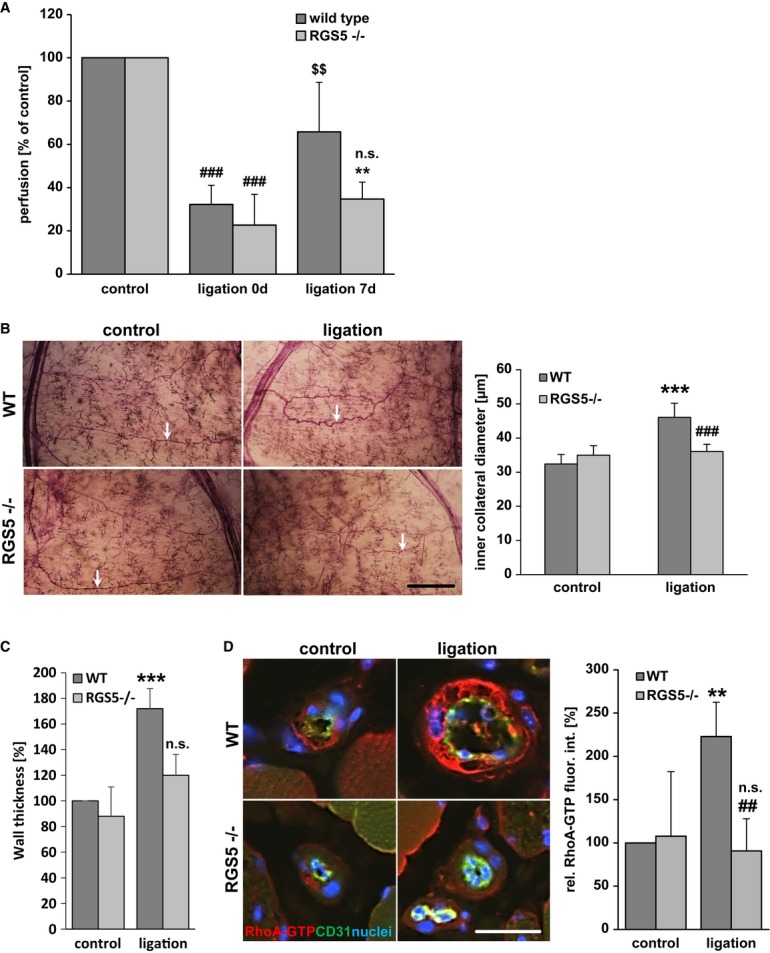
RGS5 deficiency inhibits growth of collateral arterioles A   To determine collateral-dependent blood flow recovery, hind foot perfusion was measured before, just after and 7 days after ligation. Blood flow recovery (expressed as flow in percent of non-ligated legs) was significantly attenuated in RGS5-deficient mice as compared to wild-type mice (wild type: ^###^*P* < 0.001 versus control, ^$$^*P* < 0.001 versus ligation 0 days (wild type); RGS5^−/−^: ^###^*P* < 0.001 versus control not significant (n.s.) versus ligation 0 days (RGS5^−/−^), **P < 0.01 versus ligation 7 days (wild type), *n* = 6–11; control values were set to 100%). B   Arteriogenic remodelling of collateral arterioles in the mouse hindlimb was analysed 7 days post ligation of the femoral artery. Growth of the remodelling arterioles (arrows) is significantly increased during this period in WT (***P < 0.001 versus control, *n* = 5) but not in RGS5-deficient mice (B, ^###^*P* < 0.001 versus WT ligation, *n* = 5; scale bar: 1 mm). C   Likewise, wall thickness is not increased during arteriogenesis in RGS5-deficient mice (***P < 0.001 versus control (WT) and not significant (n.s.) versus control (RGS5^−/−^), *n* = 5). D   Immunofluorescence detection of active RhoA (Rho-GTP) in cross sections of collateral arterioles revealed a significant increase in RhoA activity during arteriogenesis in WT but not in RGS5-deficient mice (**P < 0.01 versus control (WT), ^##^*P* < 0.01 versus ligation (WT) and not significant (n.s.) versus control (RGS5^−/−^), *n* = 5, analysing up to three collaterals per animal; scale bar: 25 μm).

**Figure 6:**
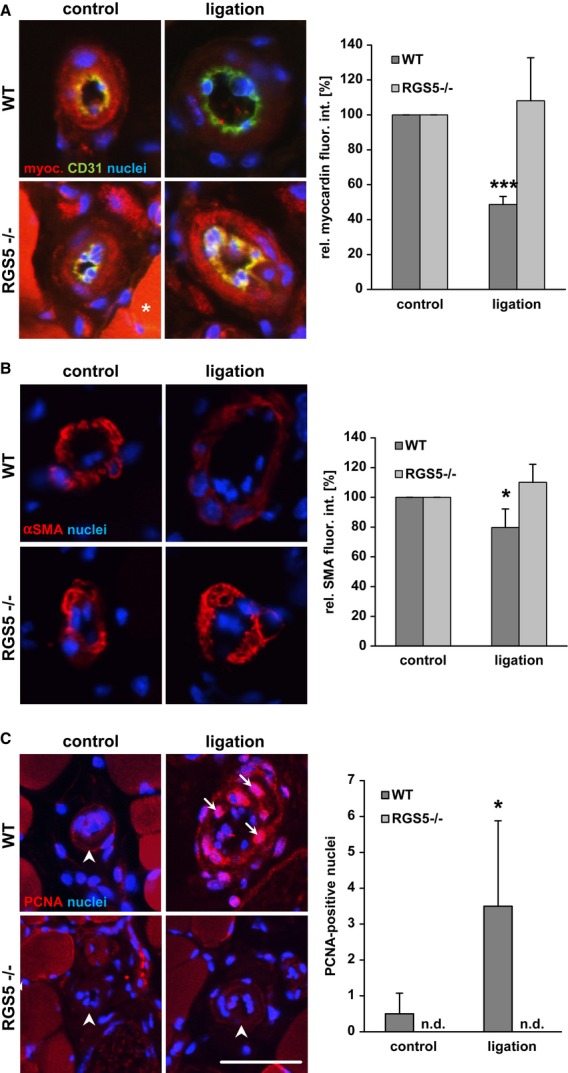
RGS5 deficiency preserves the differentiated SMC phenotype A, B   Immunofluorescence analyses of cross sections of arterioles undergoing arteriogenesis revealed a significant decline in myocardin (A) and alpha-smooth muscle actin (B) abundance in WT but not in RGS5-deficient mice over this period (*P < 0.05, ***P < 0.001 versus control, *n* = 5, analysing up to 3 collaterals per animal; asterisk: myoglobin-related red background fluorescence). C   Likewise, PCNA-positive nuclei (C, red fluorescence, arrows) indicating proliferating cells were detected in the SMCs of remodelling collaterals of WT mice but not in RGS5-deficient mice (*P < 0.05 versus control, *n* = 5; n.d: none detected; arrowheads indicate collateral arterioles; scale bar: 50 μm).

**Figure 7:**
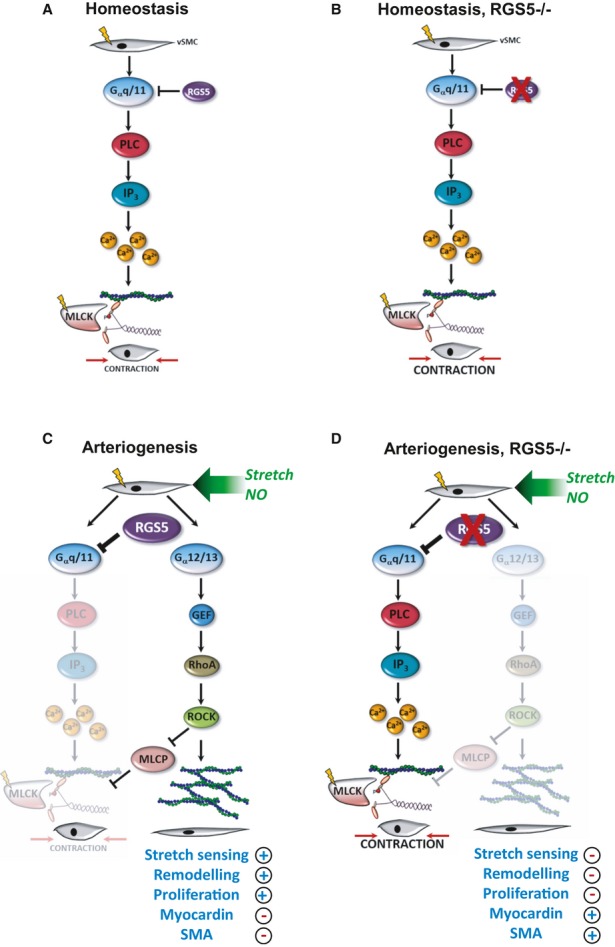
Impact of RGS5 on SMC function A   Contractile responses of vascular smooth muscle cells (vSMC) were evoked by Gα_q/11_ signalling which is sparsely inhibited under baseline conditions. B   Loss of RGS5 enhances these responses to some extend and leads to a slight increase in blood pressure. C   The increase in biomechanical stretch and release of nitric oxide (NO) triggers the expression of RGS5 during the onset of arteriogenesis, thereby favouring RhoA-mediated signal responses which promote biomechanical sensitivity of SMCs and the activated SMC phenotype. D   In RGS5-deficient mice, RhoA signalling is not amplified impairing the SMC phenotype switch under these conditions.

It is well established that RhoA signalling is crucial for the growth of collateral vessels during arteriogenesis (Eitenmuller *et al*, 2006; Troidl *et al*, 2009a). Based on the aforementioned findings, we therefore hypothesized that the absence of RGS5-dependent amplification of the RhoA pathway will hamper arteriogenesis. To pursue this idea, RGS5-deficient mice were utilized which display no obvious defects in the architecture, morphology and development of the vasculature (Supplementary Fig S9) (Nisancioglu *et al*, 2008) or ischaemia-induced angiogenesis (Supplementary Fig S10). As evidenced by telemetric blood pressure recordings, these mice have a slightly but not significantly increased mean arterial blood pressure (Supplementary Fig S11) as compared to wild-type mice but show normal cardiac functions (data not shown). Analyses of arteriogenic remodelling in RGS5-deficient mice revealed that the blood flow recovery was in fact severely impaired (Fig 5A). As the overall perfusion level of the lower limb does not clearly distinguish between angiogenesis and arteriogenesis, the diminished growth of collateral arterioles was additionally verified by morphometrical analyses (Fig 5B and C). Subsequent immunofluorescence analyses in remodelling collaterals by utilizing a specific antibody against (active) RhoA-GTP indicated that RhoA activation was more pronounced in SMCs of wild-type but not RGS5-deficient mice (Fig 5D). Furthermore, the abundance of myocardin and its target gene product smooth muscle actin was decreased in SMCs of wild-type mice but remained unchanged in RGS5-deficient mice (Fig 6A and B). These data indicate a failure of the SMCs in acquiring an activated phenotype which was also supported by the lack of SMC proliferation in the remodelling collaterals in these animals (Fig 6C) as a prototypic response during arteriogenesis. Moreover, this lack of arteriogenic SMC activation which usually promotes a distinct pro-inflammatory response through release of MCP-1 (Heil *et al*, 2004; Demicheva *et al*, 2008) may be the reason for the diminished recruitment of macrophages in RGS5-deficient mice (Supplementary Fig S12) (Banai *et al*, 1998; Waltenberger *et al*, 1999; Shibata *et al*, 2001; Matsumoto *et al*, 2004). Collectively, our findings suggest that RGS5 expression during arteriogenesis reinforces RhoA-mediated signal responses and promotes an activated SMC phenotype. In RGS5-deficient mice, Gα_q/11_ signalling prevails and maintains a quiescent and differentiated SMC phenotype under these conditions (Fig 7).

## Discussion

Downstream signalling of Gα subunits into the cell and the resulting alterations in SMC tone and phenotype are subject to regulation by different RGS proteins (Hepler *et al*, 1997). Intriguingly, the genes encoding RGS proteins 2, 4 and 5 are clustered on chromosome 1 within a locus that has been associated with the development of hypertension (Gu *et al*, 2009). RGS2-deficient mice for instance, are hypertensive (Heximer *et al*, 2003; Tang *et al*, 2003; Gross *et al*, 2005), show enhanced signalling through Gα_q/11_ and respond differently to treatment with Ang II or the α-adrenoceptor agonist norepinephrine (Heximer *et al*, 2003). RGS4 has been implicated in left ventricular hypertrophy in mice (Tokudome *et al*, 2008; Wang *et al*, 2008) as well as in inhibiting Gα_q/11_ signalling in cardiac myocytes (Tamirisa *et al*, 1999; Mittmann *et al*, 2002). Simultaneously, these proteins may affect SMC differentiation as baseline activity of G-protein signalling is essential for the phenotypic switch of SMCs and therefore for proliferation during vascular remodelling processes (Althoff *et al*, 2012). This study focused on the impact of RGS5—an inhibitor of Gα_q/11_ signalling—on arteriogenesis as a prototypic biomechanically induced adaptive arterial remodelling process. As compared to its closely related R4 family members RGS2, RGS4 and RGS16, RGS5 is only sparsely expressed in arterial SMCs (Supplementary Fig S13) and can hardly be detected *in vivo* in SMCs of collateral arterioles during vascular homeostasis. In addition, RGS5 deficiency does not induce any compensatory increase in the expression of other R4 RGS homologous (Supplementary Fig S13).

We demonstrated that arteriogenesis stimulates RGS5 expression in SMCs of collateral arterioles, presumably due to their prolonged exposure to increased wall stress or stretch which has been shown to be pivotal for these remodelling processes (Heerkens *et al*, 2007; Demicheva *et al*, 2008). In fact, prolonged stretching of cultured SMCs—mimicking an increase in wall stress *in vivo*—robustly increased RGS5 expression (Fig 2D). While medial SMCs can reduce wall stress temporarily through active constriction, long-term exposure requires remodelling of the vessel wall, typically resulting in an increase in SMC mass. This SMC hyperplasia (arterioles) or hypertrophy (conduit arteries) requires a shift from the resting to the activated phenotype (Scholz *et al*, 2000; Davis-Dusenbery *et al*, 2011) which is characterized, for example, by a decline in myocardin, a pivotal determinant of the expression of SMC marker proteins (Pipes *et al*, 2006; Parmacek, 2007; Pfisterer *et al*, 2012). Thus, RGS5 expression seems to correlate temporarily and spatially with adaptive remodelling processes in the arterial vessel wall.

Flow-induced vasodilation through enhanced shear stress-dependent NO release (Busse *et al*, 1985) from the collateral arteriolar endothelial cells figures prominently in the onset of arteriogenesis (Yu *et al*, 2005; Demicheva *et al*, 2008; Troidl *et al*, 2009b; Dai & Faber, 2010), presumably due to the concomitant flow-dependent increase in wall stress (Demicheva *et al*, 2008). Moreover, our results suggest that continuous exposure of arterial SMCs to NO in fact up-regulates RGS5 expression via the soluble guanylyl cyclase/protein kinase G pathway. Together with the increase in wall stress, this may cause the pronounced increase in RGS5 expression occurring in the SMCs of the remodelling arterioles during arteriogenesis. However, mouse models triggering the formation of collaterals are limited because they are usually based on a sudden occlusion of a conduit artery to alter biomechanical load within the collateral circulation. In contrast, arteriogenesis in human patients occurs slowly by the progressive occlusion of conduit arteries due to arteriosclerosis.

Experimental evidence delineating the functional consequences of an altered expression of RGS5 in vascular SMCs is rather scarce. Therefore, we employed both a loss-of-function and a gain-of-function approach via siRNA-based knockdown and adenoviral overexpression of the protein, respectively. Taken together, both approaches establish RGS5 as an important regulator of the SMC phenotype that attenuates Gα_q/11_ signalling by accelerating the GTP hydrolysis rate of this G-protein subunit (Gu *et al*, 2009). In agreement with our hypothesis that increased RGS5 expression blocks Gα_q/11_-phospholipase Cβ-inositol-1,4,5-trisphosphate-mediated calcium mobilization, overexpression of RGS5 in airway SMCs negatively impacts GPCR-mediated mobilization of intracellular calcium and subsequent constriction (Yang *et al*, 2012). Moreover, RGS5 allows for an enhanced Gα_12/13_-mediated activation of RhoA in SMCs not only by suppressing Gα_q/11_ activity but also by sequestration of the active protein. This would allow a predominant coupling of agonist-activated receptors to Gα_12/13_ subunits and therefore increase the activity of RhoA- and Rho-dependent signalling pathways.

RhoA is in fact involved in SMC contraction as it inhibits MLCP activity through ROCK. Nevertheless, RhoA activity may not directly affect the SMC phenotype, but it may act context-dependently to control chronic adaptive changes in the cytoskeleton (e.g. stress fibre formation) which is a prerequisite for SMCs to adequately respond to changes in biomechanical forces especially biomechanical stretch (Zhao *et al*, 1995; Numaguchi *et al*, 1999; Liu *et al*, 2007; Wojtowicz *et al*, 2010). Consequently, promoting a sustained RhoA activity by shifting Gα_q/11_ to Gα_12/13_ signalling appears to be rate limiting for arteriogenesis as this remodelling process is strictly dependent on Rho-dependent signalling (Eitenmuller *et al*, 2006) and adequate SMC responses to biomechanical stress (Demicheva *et al*, 2008). Likewise, the Rho signalling pathway also controls SMC proliferation during arteriogenesis (Troidl *et al*, 2009a), which was absent in RGS5-deficient mice. Despite solid evidence indicating that RhoA signalling promotes the expression of SMC differentiation markers (Wamhoff *et al*, 2004; Althoff *et al*, 2012; Pagiatakis *et al*, 2012), activation of this pathway in biomechanically stimulated SMCs may have a different outcome. In this context, Rho-dependent regulation of subcellular ERK1/2 localization (Zuckerbraun *et al*, 2003) may control the activity of myocardin, which is dependent on this kinase in SMCs exposed to biomechanical stress (Pfisterer *et al*, 2012). In fact, inhibition of Rho kinase has been shown to suppress stretch-induced ERK activation (Numaguchi *et al*, 1999) which underlines the relevance of the Rho signalling pathway for regulating adequate SMC responses to biomechanical stimuli. Moreover, Rho-mediated signalling in biomechanically stimulated SMCs controls the activity of the pro-inflammatory transcription factor activator protein 1 (AP-1) (Cattaruzza *et al*, 2001; Mohamed & Boriek, 2010), and blockade of Rho activity prevents vascular inflammation (e.g. diminished MCP-1 expression and macrophage infiltration) during stent-induced neointima formation (Matsumoto *et al*, 2004). Considering that AP-1-mediated pro-inflammatory responses of SMCs are rate-limiting for arteriogenesis (Demicheva *et al*, 2008), inadequate RhoA activity is likely to be incompatible with collateral growth and may contribute to the lack of macrophage infiltration during arteriogenesis in RGS5-deficient mice (Supplementary Fig S12). In conclusion, sustained repression of Rho signalling severely hampers the phenotype switch in SMCs during arteriogenesis which is consistent with our observation that activation of RhoA was blunted in RGS5-deficient mice.

Besides arteriogenesis, Rho signalling controls SMC proliferation, apoptosis and migration and thus plays an important role in neointima formation (Shibata *et al*, 2001; Matsumoto *et al*, 2004). Considering our findings, RGS5 may amplify this process by promoting RhoA activity. In fact, neointima formation induced by ligating the carotid artery (Kumar & Lindner, 1997; Kumar *et al*, 1997) was attenuated in RGS5-deficient mice (Supplementary Fig S14). Although this type of vascular remodelling is triggered by different biomechanical and molecular stimuli than arteriogenesis, these findings underline the importance of RGS5 in controlling the activity of SMCs during adaptive changes of the arterial vessel wall. Consequently, pharmaceutical modulation of RGS5 activity by small molecule approaches, as has been shown for RGS4 (Roman *et al*, 2007), may have great clinical relevance with respect to stimulating arteriogenesis or counteracting neointima formation during arteriosclerosis or in-stent restenosis. On the one hand, inhibition of RGS5 activity may block vascular remodelling by abolishing the amplification of the Rho signalling pathway. On the other hand, depending on the local activity of RGS5 in distinct vascular entities, RGS5 inhibition may have complex consequences on the regulation of the local myogenic tone. For instance, PPARγ- and PPARδ-controlled expression of RGS5 has been reported to blunt angiotensin II-mediated activation of protein kinase C, controlling the myogenic tone of mesenteric arteries (Ketsawatsomkron *et al*, 2012).

In line with the inhibitory effect of an increased level of RGS5 on GPCR agonist-evoked calcium mobilization in arterial SMCs, RGS5-deficient arteries showed an improved vasoconstrictor response. Likewise, arterial SMCs derived from RGS5-deficient mice not only revealed an enhanced sensitivity to GPCR agonist-induced signalling via Gα_q/11_, but also a distinct deficit in stress fibre formation already at baseline. In contrast, RGS5-deficient mice have repeatedly been reported to be hypotensive (Cho *et al*, 2008; Nisancioglu *et al*, 2008) or hypertensive (Holobotovskyy *et al*, 2013). The discrepancies in these findings are likely to be based on the use of different techniques (i.e. tail cuff versus telemetric measurement), genetic backgrounds and stress levels (increased cardiac output/systolic blood pressure). Our own telemetric blood pressure recordings show instead that mice utilized in our study have a modestly but not significantly increased systolic and diastolic blood pressure.

Collectively, our findings reveal that (i) vascular remodelling and the switch in SMC phenotype during arteriogenesis is accompanied by and dependent on up-regulation of RGS5 in arteriolar SMCs and (ii) RGS5 shifts Gα_q/11_-PLC_β_-MLCK-mediated constriction to Gα_12/13_-mediated RhoA activation and subsequent stress fibre formation in arterial SMCs—a prerequisite for arteriogenesis to take place (summarized in Fig 7). Therefore, stimulation of RGS5 expression in these cells may favour such an adaptive vascular remodelling process while diminishing RGS5 expression likely impairs activation of vascular SMCs and thus attenuates remodelling of the vessel wall.

## Materials and Methods

### Materials

The anti-human/mouse RGS5 antibody was purchased from Sigma-Aldrich (GW22900, Schnelldorf, Germany). Its specificity on tissue samples was confirmed by comparing the RGS5 staining of capillary pericytes in muscle tissue of wild-type and RGS5-deficient mice (Supplementary Fig S15). The monoclonal anti-mouse CD31 antibody (clone: MEC 13.3) was obtained from Santa Cruz Biotechnology (Heidelberg, Germany). The anti-mouse αSMA antibody was purchased from Dianova (Hamburg, Germany), the anti-mouse PCNA antibody was ordered from Abcam (Cambridge, UK), the anti-mouse myocardin antibody was purchased from Santa Cruz (Heidelberg, Germany), and the anti-active-RhoA (Rho-GTP) antibody (#26904) was ordered from New East Biosciences (Pennsylvania, USA). TRITC-labelled phalloidin was obtained from Invitrogen. Bradykinin, angiotensin II, norepinephrine and NONOate were purchased from Sigma-Aldrich, sphingosine-1-phosphate was obtained from Cayman Chemical (New Orleans, USA) and 8pCPT-cGMP from BioLog (Bremen, Germany). The adenoviral RGS5, GFP and Lsc vectors were kindly provided by Prof. Wieland (Department of Experimental Pharmacology, Heidelberg University).

### Arteriogenesis mouse model

All animal studies were performed with permission of the Regional Council Karlsruhe and conformed to the Guide for the Care and Use of Laboratory Animals published by the US National Institutes of Health (NIH Publication No. 85-23, revised 1996). RGS5-deficient mice were based on a C57BL/6 background. In these mice, the RGS domain coding sequence of RGS5 was replaced by GFP (Nisancioglu *et al*, 2008). At least 10- to 12-week-old male C57BL/6 (wild type, WT) or RGS5-deficient (RGS5^−/−^) mice were anesthetized with isoflurane, and the femoral artery was ligated just distal to the origin of the deep femoral artery. Each experimental group was comprised of 5–6 mice. To assess collateral-dependent blood flow recovery, perfusion of the mouse foot was determined before, just after and 7 days after ligation by laser Doppler analysis (PeriFlux 4001 Master, Perimed, Germany). On day 7 after surgery, mice were euthanized and the left ventricle of the heart was cannulated and perfused for 2 min at 100 mmHg with Ringer solution containing 100 μM adenosine and 10 μM sodium nitroprussid at 37°C followed by zinc fixative for the purpose of immunohistolgical stainings. A 4% paraformaldehyde containing a coloured pigment (HKS Gouache 318; Schmincke, Germany) that cannot pass the capillary system was used to visualize the arterial system. Thereafter, hindlimbs were dissected and processed for histological analysis.

### Visualization of the arterial system

Mouse hindlimb specimens perfused with pigment-containing paraformaldehyde were postfixed in 4% formaldehyde (18 h) and dehydrated using a series of ethanol and isopropanol following standard protocols. The tissue was then incubated in a mixture of benzyl alcohol and benzyl benzoate (1:1, v/v) having the same refractive index of the tissue for at least 18 h. This procedure induces transparency of the tissue and allows detailed analysis of the pigment-loaded arterial system. Growing collaterals in the hindlimb were identified by their constant course on the surface of the adductor muscles facilitating their identification in transparent tissue and histological preparations (Heil & Schaper, 2004). The luminal diameter of the collateral arterioles was measured using the morphological analysis software Cell^R from Olympus (Hamburg, Germany) in at least three different sites of an individual arteriole.

### Telemetric blood pressure measurement

For blood pressure measurements in conscious, naïve male wild-type (WT) or RGS5-deficient (RGS5^−/−^) mice, telemetric devices were implanted (PA-C10, Data Sciences International) as described earlier (Mills *et al*, 2000). In brief, catheter tips were advanced into the aortic arch through the left common carotid artery. The signal transducer unit was placed into a subcutaneous pocket on the right ventrolateral side of the animal. Mice were allowed to recover after surgery for 1 week before blood pressure recording was started for five consecutive days using the Dataquest A.R.T. software 4.0. The data were acquired every 30 min for 30 s, and the means were calculated from the values obtained during 6 h either at night-time or daytime.

### Perfusion of isolated mouse arteries

C57BL/6 wild-type and RGS5-deficient mice (Nisancioglu *et al*, 2008) were sacrificed; third- or fourth-order mesenteric arteries were extracted and inserted into the chamber of a myograph (Culture Myograph, DMT, Copenhagen, Denmark) containing Tyrode's buffer. Arteries were perfused with Tyrode's buffer at a longitudinal pressure gradient of 20 mmHg (70 mmHg at the inflow and 50 mmHg at the outflow) with a resulting flow of approximately 0.07 ml/min. Constriction was induced with different concentrations of norepinephrine, and vessel diameter was measured using the VediView Software (DMT, Copenhagen, Denmark).

### Cell culture and adenoviral transduction

Human arterial smooth muscle cells (HUASMCs) were isolated from human umbilical cord arteries, and mouse smooth muscle cells were isolated from third- or fourth-order mesenteric arteries of wild-type and RGS5-deficient mice (Nisancioglu *et al*, 2008) and cultured in DMEM (Invitrogen) supplemented with 15% FCS and 50 U/ml penicillin, 50 μg/ml streptomycin and fungizone (Invitrogen, Darmstadt, Germany). To isolate umbilical cord arterial smooth muscle cells, the umbilical artery was cannulated and flushed with HBSS to remove blood and endothelial cells. The artery media were cut into pieces which were placed in a Petri dish and covered with DMEM medium containing 15% FCS and antibiotics. After 10–14 days, the smooth muscle cells which grew out of the artery were removed by trypsin treatment and transferred into a T75 cell culture flask. Mouse vascular smooth muscle cells were isolated from third- or fourth-order mesenteric arteries. After separating the vessel from surrounding fat and connective tissue, the artery was cut into pieces and incubated in 1% collagenase (Sigma-Aldrich, C0130) in DMEM with 15% FCS and antibiotics overnight. Upon centrifugation, the artery pieces were resuspended in fresh medium. Outgrowing smooth muscle cells were removed by trypsin treatment and transferred into a T75 cell culture flask. Cell type specificity was routinely checked by performing smooth muscle actin immunofluorescence stainings. Only cells cultured up to passage 3 were used throughout. The isolation of HUASMCs was approved by the local ethics committee (Heidelberg, Germany) and conformed to the principles outlined in the Declaration of Helsinki (1997). In order to expose HUASMCs to cyclic stretch, they were cultured on plastic dishes or BioFlex™ 6-well plates (Flexcell, Hillsborough, NC). Stretching was performed using a Flexcell® FX-5000™ Tension System with 13% cyclic elongation at 0.5 Hz. Cyclic elongation is needed to prevent that the cells evade the biomechanical stimulus through rearranging their focal contacts. HUASMCs were transduced (200 MOI) in 2 ml DMEM w/o supplements using recombinant adenoviruses encoding either GFP or GFP plus RGS5 or GFP plus RGS-Lsc under the control of independent CMV promoters (He *et al*, 1998). Viruses were removed after 18 h, and the experiments performed 48 h later.

### siRNA-based gene silencing

For functional studies of RGS5, an siRNA-based silencing approach was used. Control siRNA (siGENOME Non-Targeting siRNA Pool #2, Thermo Scientific, Germany) and customized RGS5-targeting siRNA (sense: CCUGAAGUCUGAAUUCAGU, antisense: CCAUGAAUGUGGACUGGCA) was purchased from Sigma-Aldrich and used at a concentration of 100 nM. HUASMCs were transfected by utilizing the MATRAsi technique (IBA bioTAGnology, Göttingen, Germany) according to the manufacturer's instructions. Transfected cells were incubated for 48 h, and the siRNA-based knockdown efficiency was verified before further usage (Supplementary Fig S4).

### Measurement of intracellular calcium mobilization

Cells were seeded in black, clear bottom 96-well plates (Greiner Bio-One, Frickenhausen, Germany) and used for adenoviral transduction as described before. Thereafter, medium was removed, and cells were loaded with 2.5 μM Rhod-4 AM (AAT Bioquest, Sunnyvale, USA) in HBSS with Ca^2+^/Mg^2+^ (PAA, Pasching, Austria) containing 20 mM Hepes and 0.001% detergent (Pluronic F12, AAT Bioquest) for 30 min at 37°C and another 30 min at room temperature in the dark. GPCR agonist-evoked mobilization of intracellular calcium was measured using the f_max_ Fluorimeter (Molecular Devices, Biberach, Germany). Relative fluorescence units were measured every 5 s for a period of 5 min. Fluorescence intensities were normalized against non-stimulated controls.

### PCR analysis

Total RNA was isolated from cultured cells or isolated mouse arteries by solid-phase extraction with the RNeasy MiniKit (Qiagen, Düsseldorf, Germany) according to the manufacturer's instructions. Subsequently, reverse transcription (RT, Sensiscript® Reverse Transcription kit, according to manufacturer's instructions) and either conventional PCR or quantitative real-time RT-PCR (qRT PCR) was performed for the target sequences; the latter was performed in a LightCycler (LC, Roche Diagnostics, Germany). Primers based on the following sequences were used for amplification (annealing temperature 60°C): huRGS5, forward: GGTGGAACCTTCCCTGAGCAGC, reverse: AGAGCGCACAAAGCGAGGCA; huRPL32, forward: AGGCATTGACAACAGGGTTC, reverse: GTTGCACATCAG-CAGCACTT; LC mRPL32, forward: GGGAGCAACAAGAAAACCAA, reverse: ATTGTGGACCAGGAACTTGC (annealing: 60°C); LC mRGS5, forward: GCGGAGAAGGCAAAGCAA, reverse: GTGGTCAATGTTCACCTCTTTAGG; LC mRGS2 forward: ATCAAGCCTTCTCCTGAGGAA, reverse: GCCAGCAGTTCATCAAATGC; LC mRGS4, forward: GGGCTGAATCGTTGGAAAAC, reverse: ATTCCGACTTCAGGAAAGCTTT; LC mRGS16, forward: CCTGGTACTTGCTACTCGCTTTT, reverse: AGCACGTCGTGGAGAGGAT. Fluorescence was monitored (excitation at 470 nm and emission at 530 nm) at the end of the annealing phase. Threshold cycle (*C*_t_) was set within the exponential phase of the PCR. Quantification of the PCR product was performed using the ΔΔ*C*_t_ method (Livak & Schmittgen, 2001). Amplification of the 60S ribosomal protein L32 (RPL32) cDNA served as an internal standard.

### Immunofluorescence analyses

Deparaffinized vessel sections and methanol-fixed HUASMCs were blocked in Tris buffer (pH 7.6) containing 1% (w/v) BSA. Primary antibodies were diluted in blocking solution as follows: RGS5 1:200, CD31 1:50, PCNA and SMA 1:500, myocardin 1:100. Samples were incubated with the antibodies for 18 h at 4°C. After rinsing, sections or cells were incubated with secondary antibodies for 2 h at room temperature, additional 10 min with 4′,6-Diamidin-2-phenylindol (DAPI, Invitrogen) in PBS and mounted in Mowiol (Calbiochem). Quantification of fluorescence intensity in cultured cells or medial SMCs in arteries was performed by utilizing the morphological analysis software Cell^R from Olympus (Hamburg, Germany). Cytoplasmic fluorescence intensities were analysed by comparing size-matched areas in three to five randomly selected microscopic fields of view. When analysing the fluorescence intensity of medial SMCs in arteries, three to five size-matched areas per section and artery were quantified. Nuclei containing PCNA were detected by a purple colour upon merging the blue DAPI-fluorescence with the corresponding red fluorescence.

### RhoA activation assay

To measure the amount of activated, that is, GTP-bound, RhoA, the RhoA G-LISA Kit (colorimetric format) from Cytoskeleton (TebuBio, Offenbach, Germany) was used. HUASMCs were cultured in 6-well plates and transfected with either control/RGS5 siRNA or GFP/RGS5 construct-containing adenoviral vectors as described above. Thirty hours after transfection, the culture medium was changed to 5% FCS-containing DMEM for another 18 h to obtain more responsive cells prior to S1P stimulation. Afterwards, cells were stimulated with 10 μM S1P for 2 min and then lysed for protein extraction and further assay procedure according to manufacturer's instructions.

### Statistical analysis

All results are expressed as mean ± SD. Differences between two experimental groups were analysed by unpaired Student's *t*-test or one sample *t*-test if applicable, with a probability value of *P* < 0.05 considered statistically significant. Differences among three or more experimental groups were analysed by one-way ANOVA followed by a Bonferroni post hoc test for selected pairs of groups, with a probability value of *P* < 0.05 considered statistically significant.

**The paper explained**

**Problem**

Recent reports underlined that the development of a collateral circulation through arteriogenesis reduces the risk of mortality due to coronary artery disease by 36%. This natural compensatory vascular remodelling process is initiated when arteriosclerotic lesions occlude conduit arteries which subsequently lead to an increase in the pre- to poststenotic pressure difference. As a consequence and depending on the (progressive) stenosis level, the local perfusion of collateral arterioles and small arteries gradually increases and promotes an adaptive growth to enhance their perfusion capacity. However, therapies promoting collateral growth are not available as the mechanisms controlling this complex arteriolar remodelling process remain elusive. To define suitable targets for corresponding pharmacologic therapies, the delineation of key molecules orchestrating the activity of vascular smooth muscle cells as basis for the reorganization of the vessel wall architecture is indispensable.

**Results**

Against this background, our study indicated for the first time that balancing of G-protein signalling in arteriolar smooth muscle cells by RGS5 is a prerequisite for arteriogenesis to occur. While RGS5 appears to be abundantly expressed in smooth muscle cells of remodelling collaterals, thereby promoting a Gα_12/13_-mediated RhoA-dependent activation of these cells, genetic ablation of RGS5 in mice impairs collateral growth by inhibiting activation of these cells as evidenced by a blockade of their proliferation and RhoA activity.

**Impact**

From a clinical point of view, enhancing the abundance and/or stability of RGS5 appears to support the activated vascular smooth muscle cell phenotype and thus may be a promising therapeutic strategy to locally promote arteriogenesis and favouring the development of natural bypasses.

## Supporting information

for this article is available online: http://embomolmed.embopress.org

## References

[b1] Adams LD, Geary RL, McManus B, Schwartz SM (2000). A comparison of aorta and vena cava medial message expression by cDNA array analysis identifies a set of 68 consistently differentially expressed genes, all in aortic media. Circ Res.

[b2] Althoff TF, Juarez JA, Troidl K, Tang C, Wang S, Wirth A, Takefuji M, Wettschureck N, Offermanns S (2012). Procontractile G protein-mediated signaling pathways antagonistically regulate smooth muscle differentiation in vascular remodeling. J Exp Med.

[b3] Banai S, Wolf Y, Golomb G, Pearle A, Waltenberger J, Fishbein I, Schneider A, Gazit A, Perez L, Huber R (1998). PDGF-receptor tyrosine kinase blocker AG1295 selectively attenuates smooth muscle cell growth *in vitro* and reduces neointimal formation after balloon angioplasty in swine. Circulation.

[b4] Berger M, Bergers G, Arnold B, Hammerling GJ, Ganss R (2005). Regulator of G-protein signaling-5 induction in pericytes coincides with active vessel remodeling during neovascularization. Blood.

[b5] Berman DM, Wilkie TM, Gilman AG (1996). GAIP and RGS4 are GTPase-activating proteins for the Gi subfamily of G protein alpha subunits. Cell.

[b6] Busse R, Trogisch G, Bassenge E (1985). The role of endothelium in the control of vascular tone. Basic Res Cardiol.

[b7] Cattaruzza M, Eberhardt I, Hecker M (2001). Mechanosensitive transcription factors involved in endothelin B receptor expression. J Biol Chem.

[b8] Cho H, Kozasa T, Bondjers C, Betsholtz C, Kehrl JH (2003). Pericyte-specific expression of Rgs5: implications for PDGF and EDG receptor signaling during vascular maturation. FASEB J.

[b9] Cho H, Park C, Hwang IY, Han SB, Schimel D, Despres D, Kehrl JH (2008). Rgs5 targeting leads to chronic low blood pressure and a lean body habitus. Mol Cell Biol.

[b10] Dai X, Faber JE (2010). Endothelial nitric oxide synthase deficiency causes collateral vessel rarefaction and impairs activation of a cell cycle gene network during arteriogenesis. Circ Res.

[b11] Davis-Dusenbery BN, Wu C, Hata A (2011). Micromanaging vascular smooth muscle cell differentiation and phenotypic modulation. Arterioscler Thromb Vasc Biol.

[b12] Demicheva E, Hecker M, Korff T (2008). Stretch-induced activation of the transcription factor activator protein-1 controls monocyte chemoattractant protein-1 expression during arteriogenesis. Circ Res.

[b13] Dubroca C, You D, Levy BI, Loufrani L, Henrion D (2005). Involvement of RhoA/Rho kinase pathway in myogenic tone in the rabbit facial vein. Hypertension.

[b14] Eitenmuller I, Volger O, Kluge A, Troidl K, Barancik M, Cai WJ, Heil M, Pipp F, Fischer S, Horrevoets AJ (2006). The range of adaptation by collateral vessels after femoral artery occlusion. Circ Res.

[b15] Gross V, Tank J, Obst M, Plehm R, Blumer KJ, Diedrich A, Jordan J, Luft FC (2005). Autonomic nervous system and blood pressure regulation in RGS2-deficient mice. Am J Physiol Regul Integr Comp Physiol.

[b16] Gu S, Cifelli C, Wang S, Heximer SP (2009). RGS proteins: identifying new GAPs in the understanding of blood pressure regulation and cardiovascular function. Clin Sci.

[b17] He TC, Zhou S, da Costa LT, Yu J, Kinzler KW, Vogelstein B (1998). A simplified system for generating recombinant adenoviruses. Proc Natl Acad Sci USA.

[b18] Heerkens EH, Izzard AS, Heagerty AM (2007). Integrins, vascular remodeling, and hypertension. Hypertension.

[b19] Heil M, Schaper W (2004). Pathophysiology of collateral development. Coron Artery Dis.

[b20] Heil M, Ziegelhoeffer T, Wagner S, Fernandez B, Helisch A, Martin S, Tribulova S, Kuziel WA, Bachmann G, Schaper W (2004). Collateral artery growth (arteriogenesis) after experimental arterial occlusion is impaired in mice lacking CC-chemokine receptor-2. Circ Res.

[b21] Heil M, Eitenmuller I, Schmitz-Rixen T, Schaper W (2006). Arteriogenesis versus angiogenesis: similarities and differences. J Cell Mol Med.

[b22] Hepler JR, Berman DM, Gilman AG, Kozasa T (1997). RGS4 and GAIP are GTPase-activating proteins for Gq alpha and block activation of phospholipase C beta by gamma-thio-GTP-Gq alpha. Proc Natl Acad Sci USA.

[b23] Heximer SP, Knutsen RH, Sun X, Kaltenbronn KM, Rhee MH, Peng N, Oliveira-dos-Santos A, Penninger JM, Muslin AJ, Steinberg TH (2003). Hypertension and prolonged vasoconstrictor signaling in RGS2-deficient mice. J Clin Invest.

[b24] Holobotovskyy V, Manzur M, Tare M, Burchell J, Bolitho E, Viola H, Hool LC, Arnolda LF, McKitrick DJ, Ganss R (2013). Regulator of G-protein signaling 5 controls blood pressure homeostasis and vessel wall remodeling. Circ Res.

[b25] Ketsawatsomkron P, Lorca RA, Keen HL, Weatherford ET, Liu X, Pelham CJ, Grobe JL, Faraci FM, England SK, Sigmund CD (2012). PPARgamma regulates resistance vessel tone through a mechanism involving RGS5-mediated control of protein kinase C and BKCa channel activity. Circ Res.

[b26] Kumar A, Hoover JL, Simmons CA, Lindner V, Shebuski RJ (1997). Remodeling and neointimal formation in the carotid artery of normal and P-selectin-deficient mice. Circulation.

[b27] Kumar A, Lindner V (1997). Remodeling with neointima formation in the mouse carotid artery after cessation of blood flow. Arterioscler Thromb Vasc Biol.

[b28] Li J, Adams LD, Wang X, Pabon L, Schwartz SM, Sane DC, Geary RL (2004). Regulator of G protein signaling 5 marks peripheral arterial smooth muscle cells and is downregulated in atherosclerotic plaque. J Vasc Surg.

[b29] Liu WF, Nelson CM, Tan JL, Chen CS (2007). Cadherins, RhoA, and Rac1 are differentially required for stretch-mediated proliferation in endothelial versus smooth muscle cells. Circ Res.

[b30] Livak KJ, Schmittgen TD (2001). Analysis of relative gene expression data using real-time quantitative PCR and the 2(-Delta Delta C(T)) Method. Methods.

[b31] Matsumoto Y, Uwatoku T, Oi K, Abe K, Hattori T, Morishige K, Eto Y, Fukumoto Y, Nakamura K, Shibata Y (2004). Long-term inhibition of Rho-kinase suppresses neointimal formation after stent implantation in porcine coronary arteries: involvement of multiple mechanisms. Arterioscler Thromb Vasc Biol.

[b32] Meier P, Hemingway H, Lansky AJ, Knapp G, Pitt B, Seiler C (2012). The impact of the coronary collateral circulation on mortality: a meta-analysis. Eur Heart J.

[b33] Mills PA, Huetteman DA, Brockway BP, Zwiers LM, Gelsema AJ, Schwartz RS, Kramer K (2000). A new method for measurement of blood pressure, heart rate, and activity in the mouse by radiotelemetry. J Appl Physiol.

[b34] Mittmann C, Chung CH, Hoppner G, Michalek C, Nose M, Schuler C, Schuh A, Eschenhagen T, Weil J, Pieske B (2002). Expression of ten RGS proteins in human myocardium: functional characterization of an upregulation of RGS4 in heart failure. Cardiovasc Res.

[b35] Mohamed JS, Boriek AM (2010). Stretch augments TGF-beta1 expression through RhoA/ROCK1/2, PTK, and PI3K in airway smooth muscle cells. Am J Physiol Lung Cell Mol Physiol.

[b36] Nisancioglu MH, Mahoney WM, Kimmel DD, Schwartz SM, Betsholtz C, Genove G (2008). Generation and characterization of rgs5 mutant mice. Mol Cell Biol.

[b37] Numaguchi K, Eguchi S, Yamakawa T, Motley ED, Inagami T (1999). Mechanotransduction of rat aortic vascular smooth muscle cells requires RhoA and intact actin filaments. Circ Res.

[b38] Pagiatakis C, Gordon JW, Ehyai S, McDermott JC (2012). A novel RhoA/ROCK-CPI-17-MEF2C signaling pathway regulates vascular smooth muscle cell gene expression. J Biol Chem.

[b39] Parmacek MS (2007). Myocardin-related transcription factors: critical coactivators regulating cardiovascular development and adaptation. Circ Res.

[b40] Pfisterer L, Feldner A, Hecker M, Korff T (2012). Hypertension impairs myocardin function - a novel mechanism facilitating arterial remodeling. Cardiovasc Res.

[b41] Pipes GC, Creemers EE, Olson EN (2006). The myocardin family of transcriptional coactivators: versatile regulators of cell growth, migration, and myogenesis. Genes Dev.

[b42] Roman DL, Talbot JN, Roof RA, Sunahara RK, Traynor JR, Neubig RR (2007). Identification of small-molecule inhibitors of RGS4 using a high-throughput flow cytometry protein interaction assay. Mol Pharmacol.

[b43] Ross EM, Wilkie TM (2000). GTPase-activating proteins for heterotrimeric G proteins: regulators of G protein signaling (RGS) and RGS-like proteins. Annu Rev Biochem.

[b44] Schaper W (2012). Collateral vessels reduce mortality. Eur Heart J.

[b45] Scholz D, Ito W, Fleming I, Deindl E, Sauer A, Wiesnet M, Busse R, Schaper J, Schaper W (2000). Ultrastructure and molecular histology of rabbit hind-limb collateral artery growth (arteriogenesis). Virchows Arch.

[b46] Shibata R, Kai H, Seki Y, Kato S, Morimatsu M, Kaibuchi K, Imaizumi T (2001). Role of Rho-associated kinase in neointima formation after vascular injury. Circulation.

[b47] Tamirisa P, Blumer KJ, Muslin AJ (1999). RGS4 inhibits G-protein signaling in cardiomyocytes. Circulation.

[b48] Tang KM, Wang GR, Lu P, Karas RH, Aronovitz M, Heximer SP, Kaltenbronn KM, Blumer KJ, Siderovski DP, Zhu Y (2003). Regulator of G-protein signaling-2 mediates vascular smooth muscle relaxation and blood pressure. Nat Med.

[b49] Teramoto H, Crespo P, Coso OA, Igishi T, Xu N, Gutkind JS (1996). The small GTP-binding protein rho activates c-Jun N-terminal kinases/stress-activated protein kinases in human kidney 293T cells. Evidence for a Pak-independent signaling pathway. J Biol Chem.

[b50] Tokudome T, Kishimoto I, Horio T, Arai Y, Schwenke DO, Hino J, Okano I, Kawano Y, Kohno M, Miyazato M (2008). Regulator of G-protein signaling subtype 4 mediates antihypertrophic effect of locally secreted natriuretic peptides in the heart. Circulation.

[b51] Troidl K, Ruding I, Cai WJ, Mucke Y, Grossekettler L, Piotrowska I, Apfelbeck H, Schierling W, Volger OL, Horrevoets AJ (2009a). Actin-binding rho activating protein (Abra) is essential for fluid shear stress-induced arteriogenesis. Arterioscler Thromb Vasc Biol.

[b52] Troidl K, Tribulova S, Cai WJ, Eitenmuller I, Wustrack H, Schierling W, Troidl C, Schmitz-Rixen T, Schaper W (2009b). Effects of endogenous NO and of DETA NONOate in Arteriogenesis. J Cardiovasc Pharmacol.

[b53] Unthank JL, Nixon JC, Dalsing MC (1996). Inhibition of NO synthase prevents acute collateral artery dilation in the rat hindlimb. J Surg Res.

[b54] Waltenberger J, Uecker A, Kroll J, Frank H, Mayr U, Bjorge JD, Fujita D, Gazit A, Hombach V, Levitzki A (1999). A dual inhibitor of platelet-derived growth factor beta-receptor and Src kinase activity potently interferes with motogenic and mitogenic responses to PDGF in vascular smooth muscle cells. A novel candidate for prevention of vascular remodeling. Circ Res.

[b55] Wamhoff BR, Bowles DK, McDonald OG, Sinha S, Somlyo AP, Somlyo AV, Owens GK (2004). L-type voltage-gated Ca^2+^ channels modulate expression of smooth muscle differentiation marker genes via a rho kinase/myocardin/SRF-dependent mechanism. Circ Res.

[b56] Wang X, Adams LD, Pabon LM, Mahoney WM, Beaudry D, Gunaje J, Geary RL, Deblois D, Schwartz SM (2008). RGS5, RGS4, and RGS2 expression and aortic contractibility are dynamically co-regulated during aortic banding-induced hypertrophy. J Mol Cell Cardiol.

[b57] Wieland T, Mittmann C (2003). Regulators of G-protein signalling: multifunctional proteins with impact on signalling in the cardiovascular system. Pharmacol Ther.

[b58] Wieland T, Lutz S, Chidiac P (2007). Regulators of G protein signalling: a spotlight on emerging functions in the cardiovascular system. Curr Opin Pharmacol.

[b59] Wojtowicz A, Babu SS, Li L, Gretz N, Hecker M, Cattaruzza M (2010). Zyxin mediation of stretch-induced gene expression in human endothelial cells. Circ Res.

[b60] Wuertz CM, Lorincz A, Vettel C, Thomas MA, Wieland T, Lutz S (2010). p63RhoGEF–a key mediator of angiotensin II-dependent signaling and processes in vascular smooth muscle cells. FASEB J.

[b61] Yang Z, Balenga N, Cooper PR, Damera G, Edwards R, Brightling CE, Panettieri RA, Druey KM (2012). RGS5 inhibits bronchial smooth muscle contraction in severe asthma. Am J Respir Cell Mol Biol.

[b62] Yu J, deMuinck ED, Zhuang Z, Drinane M, Kauser K, Rubanyi GM, Qian HS, Murata T, Escalante B, Sessa WC (2005). Endothelial nitric oxide synthase is critical for ischemic remodeling, mural cell recruitment, and blood flow reserve. Proc Natl Acad Sci USA.

[b63] Zhang S, Han J, Sells MA, Chernoff J, Knaus UG, Ulevitch RJ, Bokoch GM (1995). Rho family GTPases regulate p38 mitogen-activated protein kinase through the downstream mediator Pak1. J Biol Chem.

[b64] Zhang W, Huang Y, Gunst SJ (2012). The small GTPase RhoA regulates the contraction of smooth muscle tissues by catalyzing the assembly of cytoskeletal signaling complexes at membrane adhesion sites. J Biol Chem.

[b65] Zhao S, Suciu A, Ziegler T, Moore JE, Burki E, Meister JJ, Brunner HR (1995). Synergistic effects of fluid shear stress and cyclic circumferential stretch on vascular endothelial cell morphology and cytoskeleton. Arterioscler Thromb Vasc Biol.

[b66] Zuckerbraun BS, Shapiro RA, Billiar TR, Tzeng E (2003). RhoA influences the nuclear localization of extracellular signal-regulated kinases to modulate p21Waf/Cip1 expression. Circulation.

