# Supplementary material for: RGS5 promotes arterial growth during arteriogenesis
Source: EMBO Mol Med. 2014 Jun 27;6(8):1075–89. doi: 10.15252/emmm.201403864 (PMC4154134; doi:10.15252/emmm.201403864)
Supplement: Supplementary file 1 [file emmm0006-1075-sd1.pdf]

Supplement Fig S1

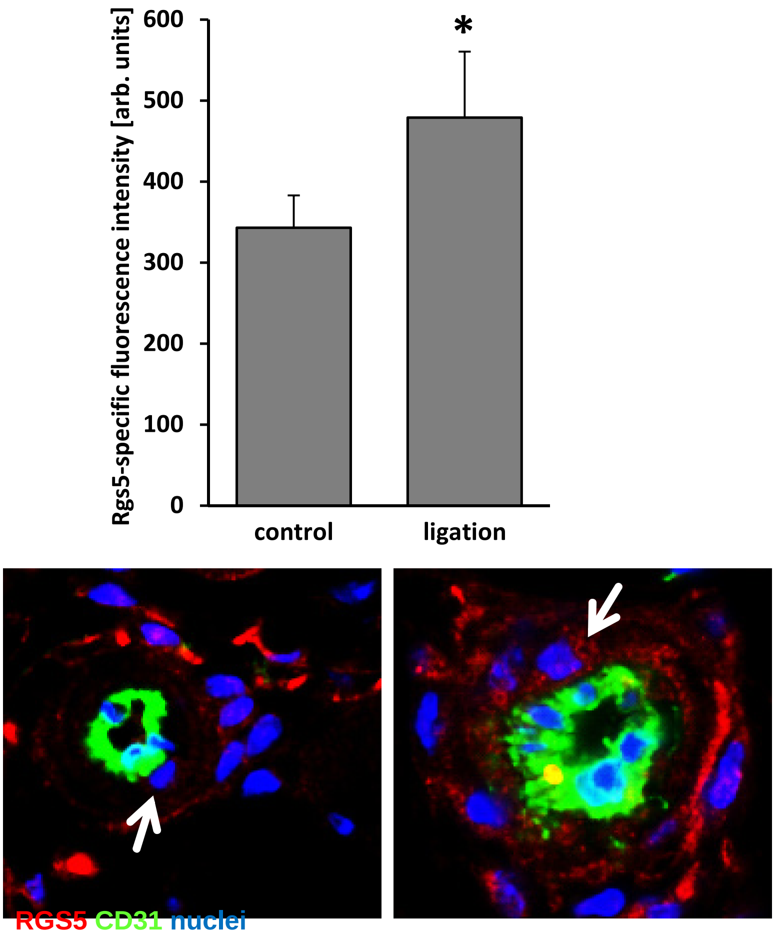

RGS5 protein abundance is increased in remodelling collateral arterioles (3d)
Arteriogenic remodelling of collateral arterioles in the mouse hindlimb was analysed 3 days post ligation of the femoral artery (early remodeling phase). Quantification of RGS5-specific immunofluorescence intensity (red fluorescence staining) in the SMCs of these arterioles (arrow) revealed a moderate rise in RGS5 abundance over this period (*p<0.05 vs. control, n=5, analysing up to 3 collaterals per animal).
