# Supplementary material for: RGS5 promotes arterial growth during arteriogenesis
Source: EMBO Mol Med. 2014 Jun 27;6(8):1075–89. doi: 10.15252/emmm.201403864 (PMC4154134; doi:10.15252/emmm.201403864)
Supplement: Supplementary file 2 [file emmm0006-1075-sd2.pdf]

# Supplement [illegible]

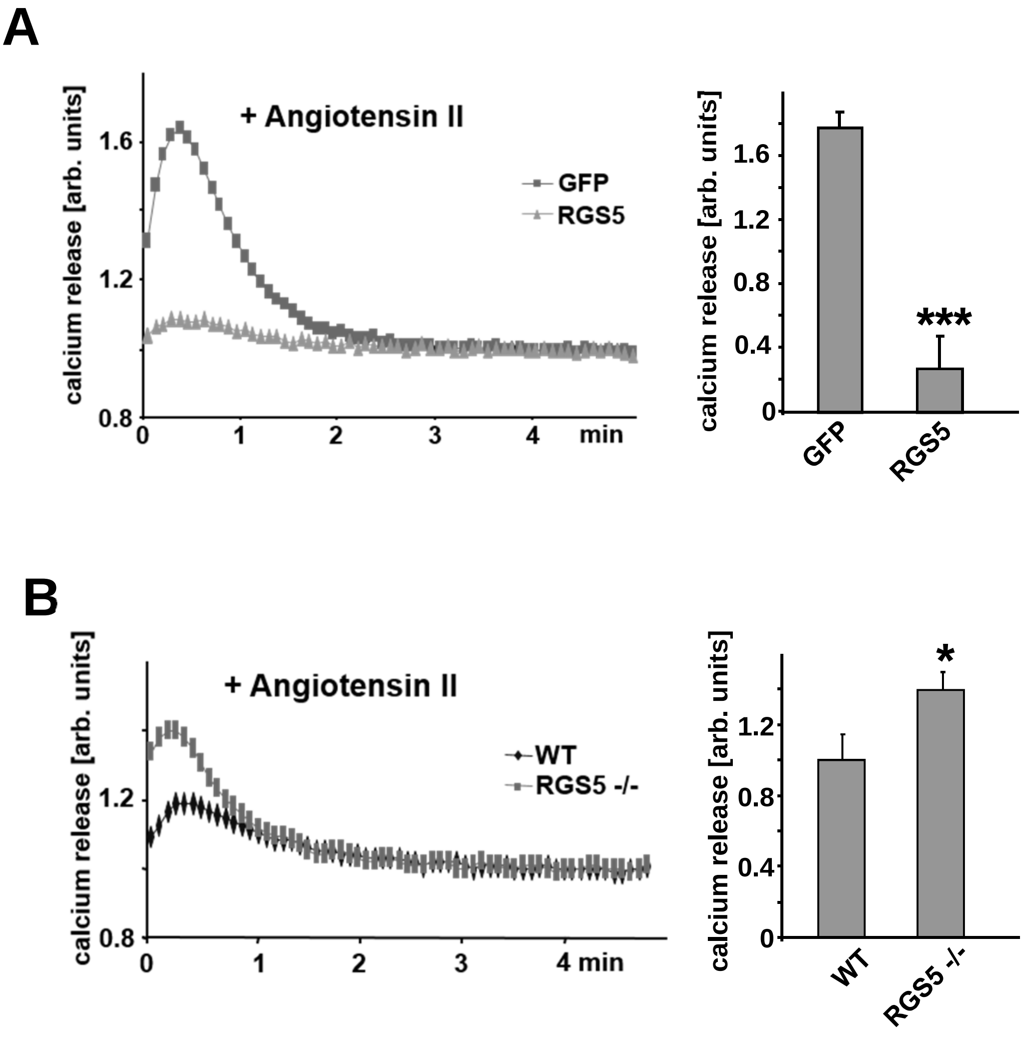


**Ang II-induced mobilization of intracellular calcium is modulated by RGS5**

Cultured human umbilical artery SMCs were transduced with an adenoviral control (GFP) or RGS5 expression vector (RGS5), and then loaded with the calcium-sensing fluorophore Rhod-4 AM. Angiotensin II (1 µmol/L, A) elicit a rapid but transient rise in intracellular calcium in GFP-expressing cells which is virtually abrogated in cells overexpressing RGS5 (***p<0.001 vs. GFP-expressing cells, n=4; calcium transients were quantified by determining the area under the curve).

Cultured arterial SMCs derived from wild type (WT) or RGS5-deficient mice (RGS5-/-) were loaded with the calcium-sensing fluorophore Rhod-4 AM. Angiotensin II (1 µmol/L, B) moderately increased the intracellular calcium concentration in control SMCs. This effect was significantly reinforced in RGS5-deficient SMCs (*p<0.05 vs. control, n=4)
