# Supplementary material for: RGS5 promotes arterial growth during arteriogenesis
Source: EMBO Mol Med. 2014 Jun 27;6(8):1075–89. doi: 10.15252/emmm.201403864 (PMC4154134; doi:10.15252/emmm.201403864)
Supplement: Supplementary file 3 [file emmm0006-1075-sd3.pdf]

# Supplementl-[illegible]

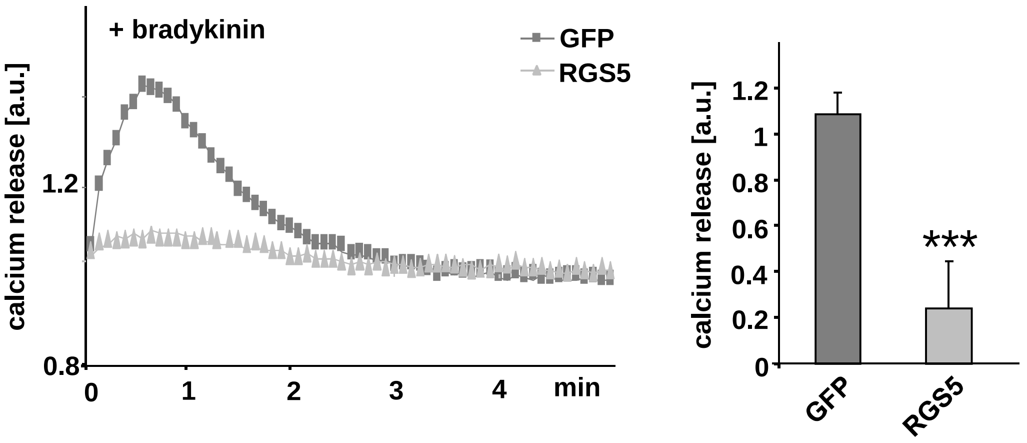


**Bradykinin-induced mobilization of intracellular calcium is modulated by RGS5**
Umbilical artery SMCs were transduced with an adenoviral control (GFP) or RGS5 expression vector (RGS5) and then loaded with the calcium-sensing fluorophore Rhod-4 AM. Bradykinin (0.01 µmol/L) elicits a rapid but transient rise in intracellular calcium in GFP-expressing cells which is virtually abrogated in cells overexpressing RGS5 (***$p<0.001$ vs. GFP-expressing cells, n=4; calcium transients were quantified by determining the area under the curve).
