# Supplementary material for: RGS5 promotes arterial growth during arteriogenesis
Source: EMBO Mol Med. 2014 Jun 27;6(8):1075–89. doi: 10.15252/emmm.201403864 (PMC4154134; doi:10.15252/emmm.201403864)
Supplement: Supplementary file 4 [file emmm0006-1075-sd4.pdf]

Supplement[illegible]

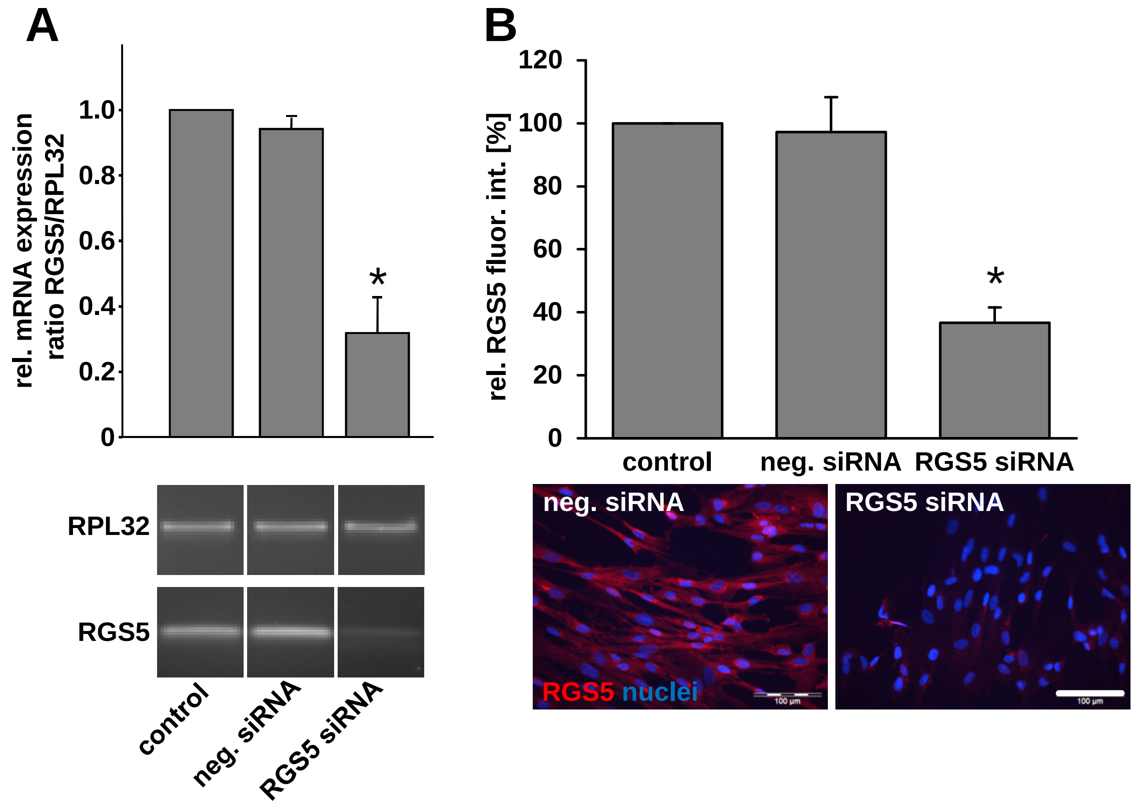

Validation of RGS5 knockdown efficiency

Human cultured umbilical artery SMCs were treated with control siRNA (neg. siRNA) and RGS5-targeting siRNA (RGS5 siRNA) or left untreated (control). Knockdown efficiency was verified by PCR indicating a robust decrease of RGS5 mRNA expression in RGS5-siRNA-treated HUASMCs but not in cells treated with neg. siRNA (A, *$p<0.05$ vs. neg. siRNA, n=4; the mRNA expression of RPL32 served as an internal standard with the values (ratio RGS5/RPL32) of untreated cells (control) set to 1). Correspondingly, immunofluorescence analyses of HUASMCs treated with RGS5 siRNA revealed a decrease in RGS5-specific (red) fluorescence (B, *$p<0.05$ vs. neg. siRNA; shown are the means±SD of one representative of two independent experiments with comparable results summarizing 5 randomly selected fields of view per condition. Control levels were set to 100%. The bottom panel shows representative fields of view with RGS5 stained in red and nuclei in blue; scale bar: 100 µm).
