# Supplementary material for: RGS5 promotes arterial growth during arteriogenesis
Source: EMBO Mol Med. 2014 Jun 27;6(8):1075–89. doi: 10.15252/emmm.201403864 (PMC4154134; doi:10.15252/emmm.201403864)
Supplement: Supplementary file 5 [file emmm0006-1075-sd5.pdf]

Supplement[illegible]

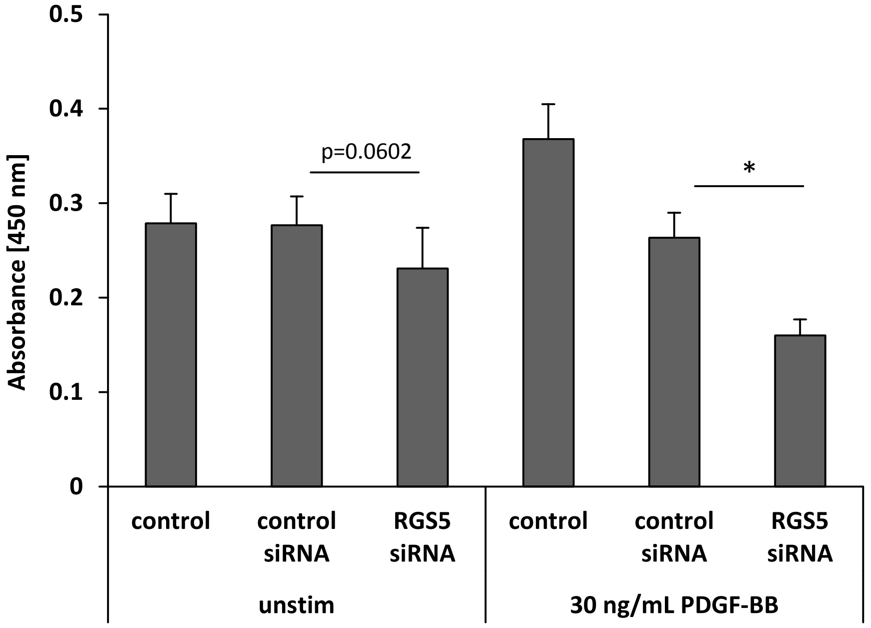

Proliferation analysis of human arterial smooth muscle cells (HUASMCs) upon knockdown of RGS5

HUASMCs were treated with control siRNA or RGS5-specific siRNA, respectively or left untreated for 48 hrs followed by addition of 30 ng/mL PDGF-BB and BrdU for 24 hrs. Afterwards, the BrdU porliferation assay was performed according to manufacturer‘s instructions (Cell Signaling). Knockdown of RGS5 in HUASMCs did not significantly affect baseline proliferation ($p=0.0602$ vs. control siRNA, pentaplicates). However, knockdown of RGS5 in PDGF-BB-stimulated HUASMCs significantly decreases proliferation (*$p<0.05$ vs. control siRNA, one out of two independent experiments with comparable results performed in pentaplicates).
