# Supplementary material for: RGS5 promotes arterial growth during arteriogenesis
Source: EMBO Mol Med. 2014 Jun 27;6(8):1075–89. doi: 10.15252/emmm.201403864 (PMC4154134; doi:10.15252/emmm.201403864)
Supplement: Supplementary file 6 [file emmm0006-1075-sd6.pdf]

# Supplement[illegible]

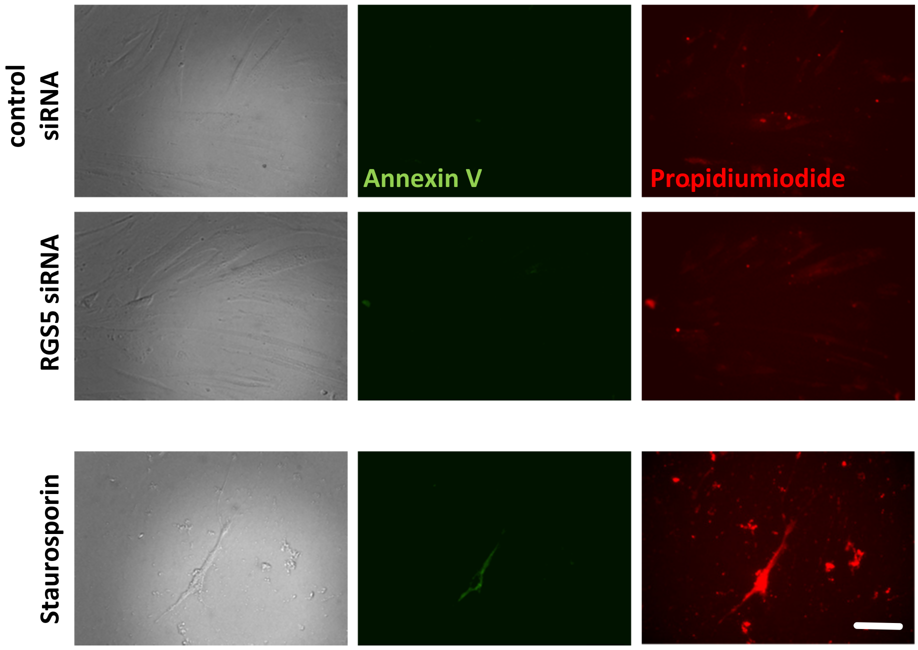


**Knockdown of RGS5 does not induce apoptosis in HUASMCs**

HUASMCs were treated with control siRNA or RGS5-specific siRNA, respectively, for 48 hrs. Untreated HUASMCs were stimulated with 1 µM staurosporin to induce apoptosis which was detected by an annexin V/propidiumiodide fluorescence staining (Trevigen). No induction of apoptosis was detected upon RGS5 knockdown. The representative images show one out of 3 independent experiments with similar results. The left panel shows bright filed images demonstrating evenly distribution of cells except after stauropsorin treatment which led to detachment of cells. The middle panel shows the Annexin V staining (green fluorescence) marking early apoptotic cells which was only detectable in straurosporine treated cells. The right panel shows the propidiumiodide staining characteristic for late apoptotic/necrotic cells (red fluorescence) which was only visible after staurosporine treatment (scale bar: 100 µm).
