# Supplementary material for: RGS5 promotes arterial growth during arteriogenesis
Source: EMBO Mol Med. 2014 Jun 27;6(8):1075–89. doi: 10.15252/emmm.201403864 (PMC4154134; doi:10.15252/emmm.201403864)
Supplement: Supplementary file 7 [file emmm0006-1075-sd7.pdf]

Supplement[illegible]

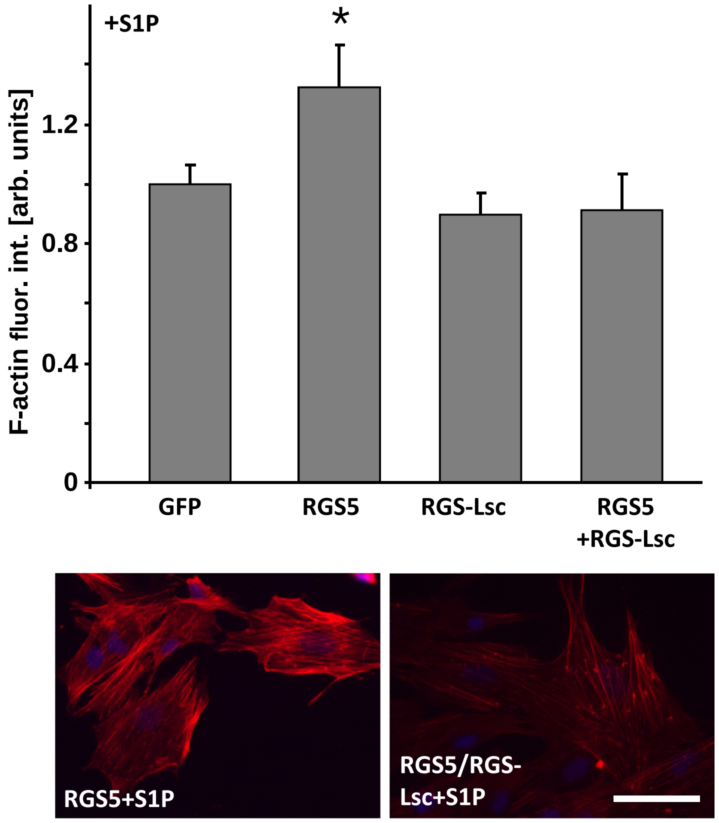

Analysis of the impact of RGS-Lsc on S1P-induced stress fiber formation in RGS5-overexpressing SMCs

Umbilical artery SMCs were transduced with an adenoviral control vector (GFP), RGS5 expression vector (RGS5) or RGS-Lsc expression vector (RGS-Lsc) alone or both RGS5 and RGS-Lsc expesion vectors. Stress fibers (F-actin) were visualized after stimulating the cells with S1P (15 min, 10 µmol) and exposing them to TRITC-labelled phalloidin (1:200) for 20 minutes.

Stress fiber formation in S1P-stimulated, RGS5-over-expressing cells was enhanced while simultaneous overexpression of RGS5 and RGS-Lsc abrogated this effect (*p<0.05 vs. control GFP-overexpressing cells; shown are the means±SD of one experiment summarizing three randomly selected fields of view per condition determining the cumulative phalloidin fluorescence intensity of at least four (transduced) cells, scale bar 20 µm). This result suggests that S1P-stimulated Rho-dependent stress fiber formation in RGS5-overexpressing cells is due to the activation of $G_a12/13$ and subsequent RGS-Lsc activation.
