# Supplementary material for: RGS5 promotes arterial growth during arteriogenesis
Source: EMBO Mol Med. 2014 Jun 27;6(8):1075–89. doi: 10.15252/emmm.201403864 (PMC4154134; doi:10.15252/emmm.201403864)
Supplement: Supplementary file 8 [file emmm0006-1075-sd8.pdf]

Supplementl-Ǌề CƛƎ {y

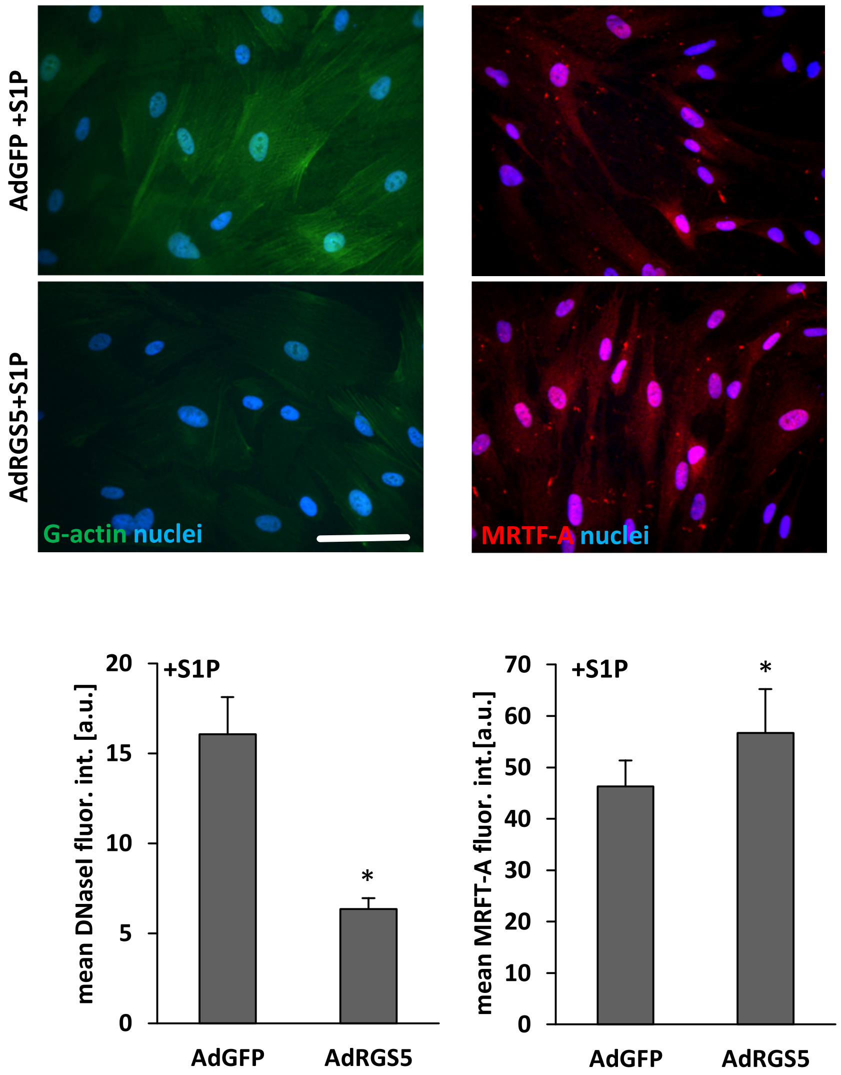

RGS5 over-expression in human arterial smooth muscle cells (HUASMCs) affects G-actin and MRTF-A localization

HUASMCs were transduced with adenoviruses expressing GFP (AdGFP) or RGS5 (AdRGS5) in serum-free medium for 48 hrs. Cells were stimulated with 10 µM S1P and processed for fluorescence detection of globular actin (monomeric G-actin binds to fluorescence-labeled DNaseI, green fluorescence) and MRTF-A (red immunofluorescence). Nuclei were visualized with DAPI (blue fluorescence). RGS5 over-expressing HUASMCs exhibited a decrease of DNaseI fluorescence intensity and more MRTF-A-positive nuclei as compared to AdGFP-transfected cells (*<p0.05 vs. AdGFP, one out of three experiments with comparable results performed in triplicates, scale bar: 100 µm).
