# Supplementary material for: RGS5 promotes arterial growth during arteriogenesis
Source: EMBO Mol Med. 2014 Jun 27;6(8):1075–89. doi: 10.15252/emmm.201403864 (PMC4154134; doi:10.15252/emmm.201403864)
Supplement: Supplementary file 9 [file emmm0006-1075-sd9.pdf]

Supplement[illegible]

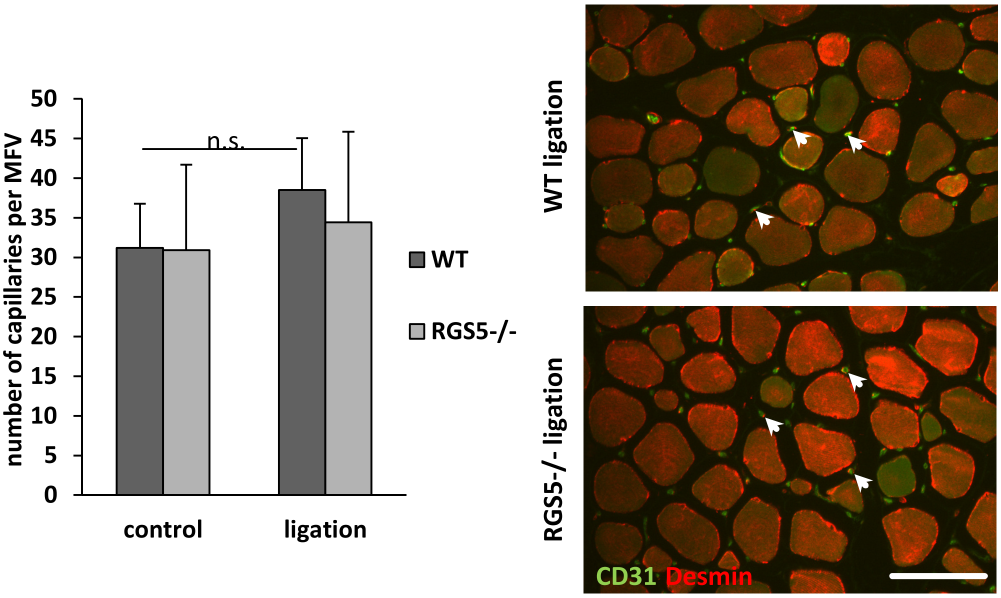

Comparison of the capillary density in the adductor muscle of WT and RGS5-/- mice
Seven days after ligation of the femoral or control surgery, adductor muscle tissue (is not affected by hypoxia under these conditions) was dissected, fixed in zinc fixative, embedded in paraffin and sectioned. The graph shows the mean number of capillaries +SD per microscopic field of view (MFV) in the adductor muscle tissue of WT and RGS5-/- mice (n=5, determining CD31-positive capillaries in at least two MFV per animal and condition). No significant differences were observed either under control conditions or after femoral artery ligation between WT and RGS5-/- mice (n.s - $p>0.05$ vs. WT control). Representative images are shown on the right. White arrows identify desmin-positive (red fluorescence) endothelial cells (green fluorescence) indicating no defect in pericyte coverage (maturation) under these conditions (scale bar: 100 µm).
