# Supplementary material for: RGS5 promotes arterial growth during arteriogenesis
Source: EMBO Mol Med. 2014 Jun 27;6(8):1075–89. doi: 10.15252/emmm.201403864 (PMC4154134; doi:10.15252/emmm.201403864)
Supplement: Supplementary file 10 [file emmm0006-1075-sd10.pdf]

Supplement[illegible]

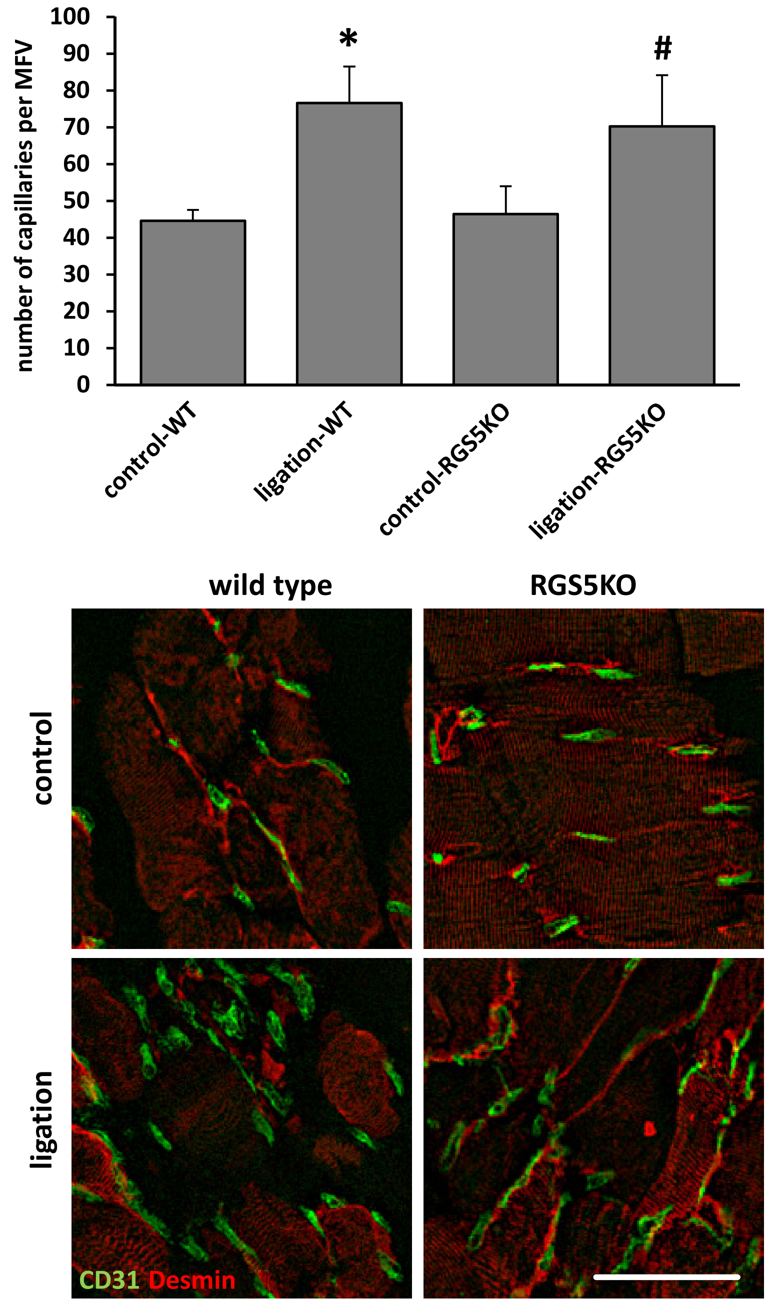

Comparison of the capillary density in the ischemic calf muscle of WT and RGS5-/- mice

Three days after induction of ischemia through ligating the femoral artery calf muscle tissue was dissected, fixed in zinc fixative, embedded in paraffin and sectioned. The graph shows the mean number of capillaries +SD per microscopic field of view (MFV) in the calf muscle tissue of WT and RGS5-deficient (RGS5KO) mice (n=5, determining CD31-positive capillaries in at least two MFV per animal and condition). Ligation-induced ischemia stimulated an increase in capillary density in muscle tissues of both wild type (WT) and RGS5KO mice (*$p<0.05$ vs. WT control, #$p<0.05$ vs. RGS5KO control). Representative images show CD31-positive endothelial cells (green fluorescence, scale bar: 50 µm).
