# Supplementary material for: RGS5 promotes arterial growth during arteriogenesis
Source: EMBO Mol Med. 2014 Jun 27;6(8):1075–89. doi: 10.15252/emmm.201403864 (PMC4154134; doi:10.15252/emmm.201403864)
Supplement: Supplementary file 11 [file emmm0006-1075-sd11.pdf]

Supplemen[illegible]

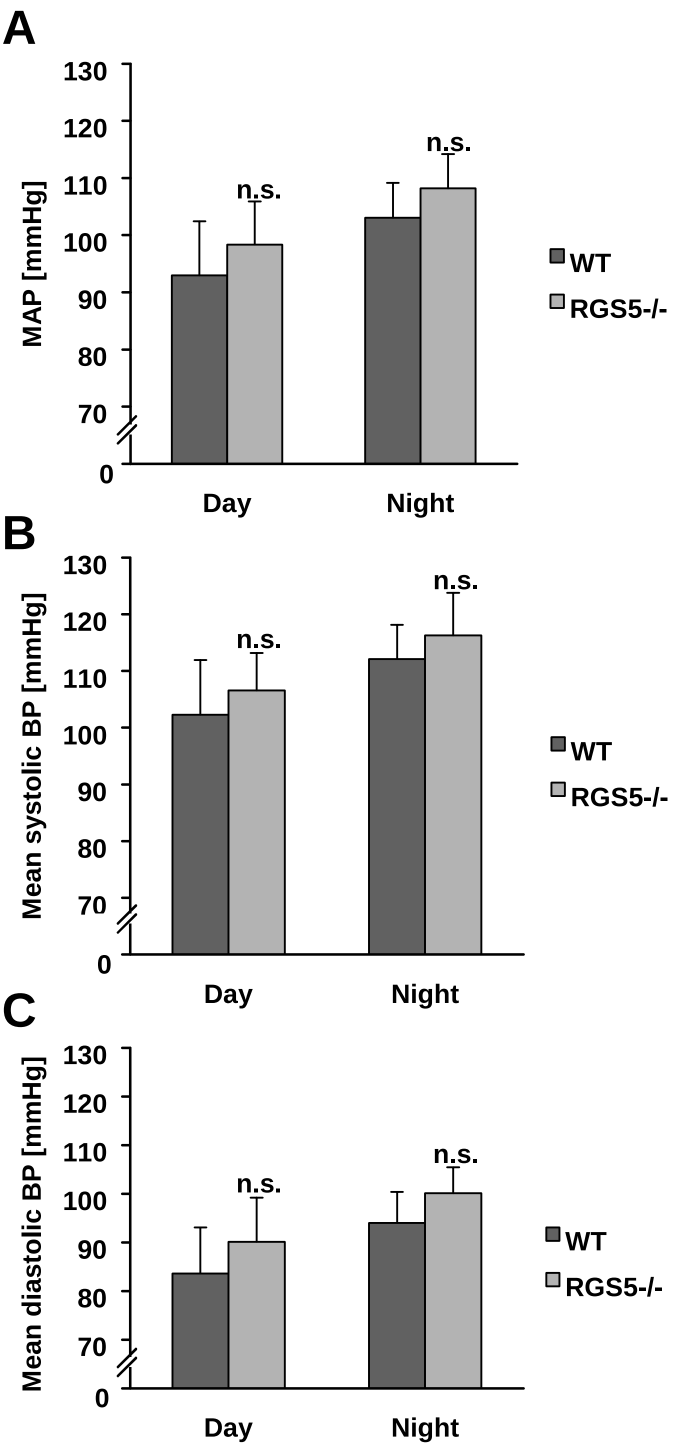

Telemetric blood pressure analysis in wild type and RGS5-deficient mice
Mean (MAP), systolic and diastolic arterial blood pressure values were continuously (6 hours, every 30 min for 30 s) recorded in wild type (WT) and RGS5-deficient (RGS5-/-) mice. A slight but not significant increase in these blood pressure values was observed (A-C, shown are the means±SD recorded at day- or nighttime, n.s. – not significant vs. WT mice, n=4).
