# Supplementary material for: RGS5 promotes arterial growth during arteriogenesis
Source: EMBO Mol Med. 2014 Jun 27;6(8):1075–89. doi: 10.15252/emmm.201403864 (PMC4154134; doi:10.15252/emmm.201403864)
Supplement: Supplementary file 12 [file emmm0006-1075-sd12.pdf]

# Supplement [illegible]

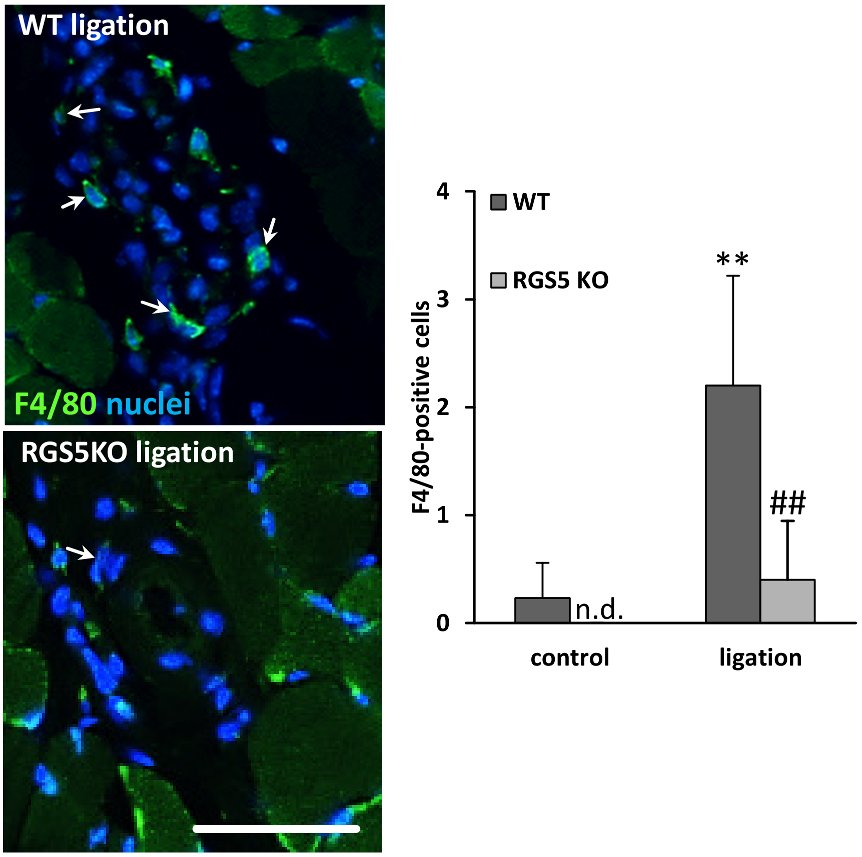


**Comparison of arteriogenesis-associated macrophage recruitment in WT and RGS5-/- mice**
Seven days after ligation of the femoral artery the number of macrophages (F4/80-positive cells, arrows, green fluorescence) in the media/adventitia of collateral arterioles was determined. While arteriogenic remodeling in wild type (WT) is associated with a significant increase in macrophage infiltration (**p<0.01 vs. control WT, n=5), decreased numbers of collateral-associated macrophages were detected in RGS5-deficient (RGS5KO; ##p<0.01 vs. WT ligation) mice (scale bar: 40 µm).
