# Supplementary material for: RGS5 promotes arterial growth during arteriogenesis
Source: EMBO Mol Med. 2014 Jun 27;6(8):1075–89. doi: 10.15252/emmm.201403864 (PMC4154134; doi:10.15252/emmm.201403864)
Supplement: Supplementary file 13 [file emmm0006-1075-sd13.pdf]

Supplement [illegible]

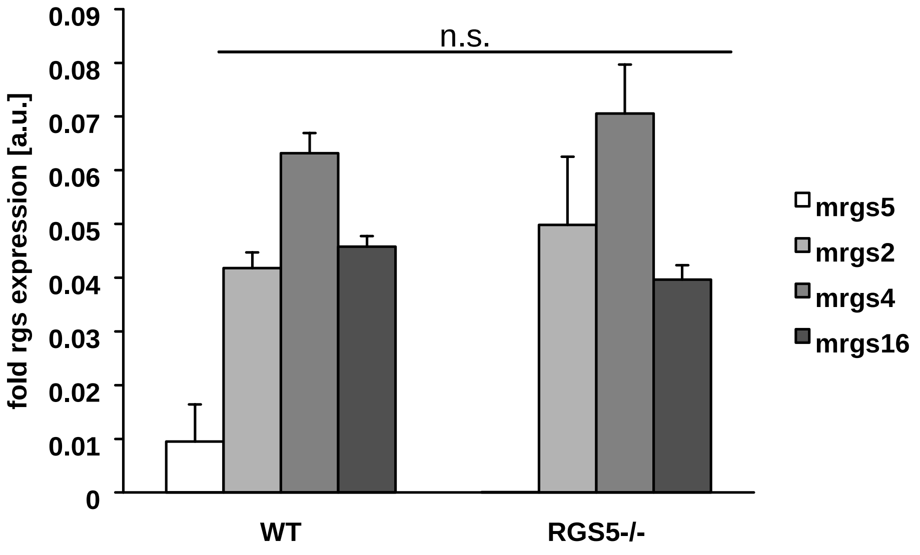

Comparison of the RGS expression in wild type and RGS5-deficient mice

Quantitative real time PCR analyses of mRNA from wild type (WT) or RGS5-deficient (RGS5-/-) arteries revealed a low expression level of RGS5 as compared to other related RGS R4 family members (n=4; the mRNA expression of mRPL32 served as an internal standard, shown are the means±SD). The loss of RGS5 is not compensated by an increase in mRNA expression of other RGS family members. This may explain for the unincisive phenotype of these mice under regular conditions and is compatible with the blood pressure values.
