# Supplementary material for: RGS5 promotes arterial growth during arteriogenesis
Source: EMBO Mol Med. 2014 Jun 27;6(8):1075–89. doi: 10.15252/emmm.201403864 (PMC4154134; doi:10.15252/emmm.201403864)
Supplement: Supplementary file 14 [file emmm0006-1075-sd14.pdf]

Supplement[illegible]

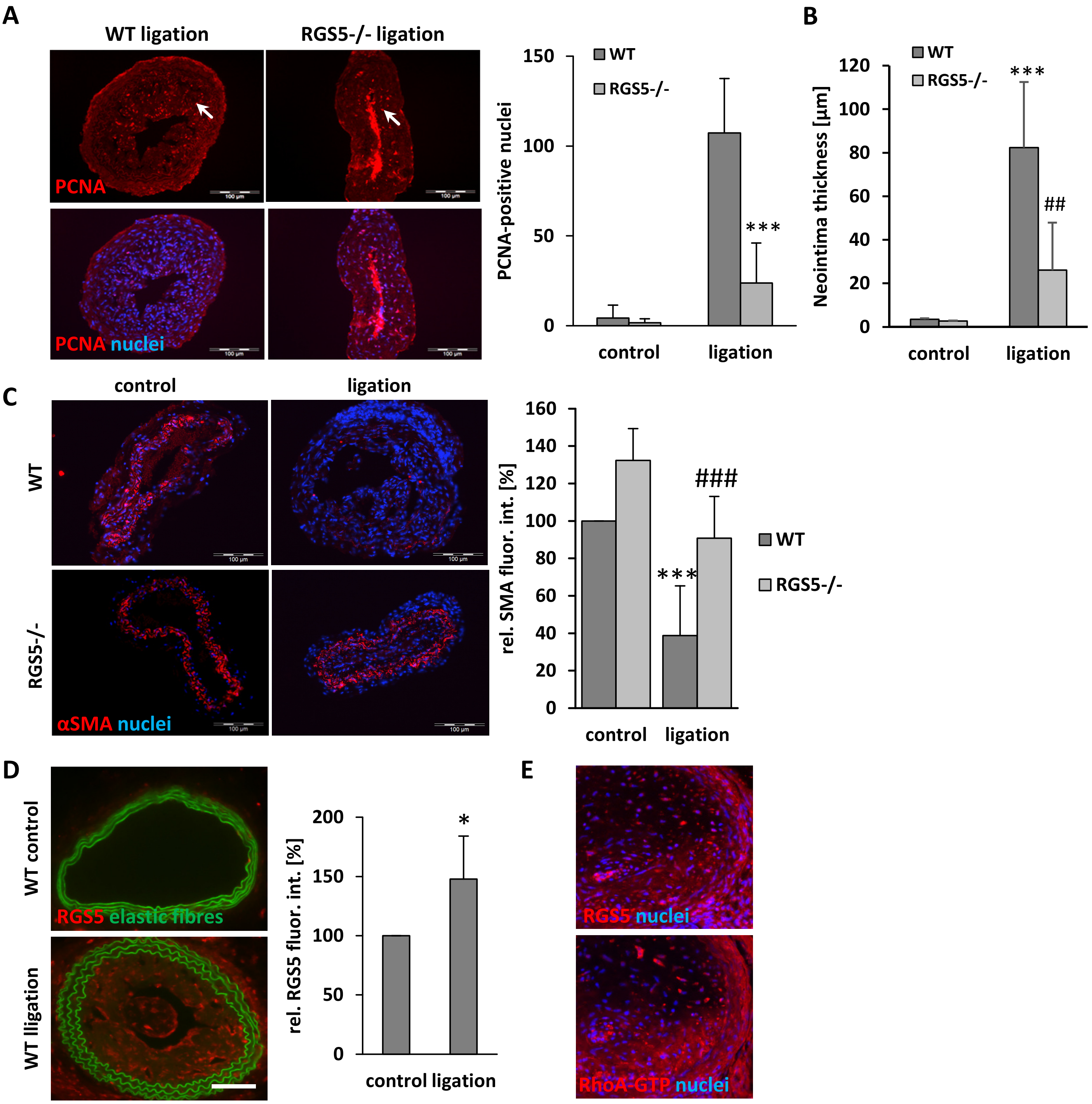

Comparison of neointima formation in wild type (WT) and RGS5-/- mice

The left common carotid artery was occulded proximal to the bifurcation of the external and internal carotid artery to induce flow cessation in the distal part of that artery. After 4 weeks carotids were harvested, fixed in zinc fixative, embedded in paraffin and sectioned. Proliferation of neointimal cells was significantly decreased in RGS5-deficient mice as evidenced by staining of the proliferation marker PCNA (A, ***$p<0.001$ vs. WT ligation; PCNA - red fluorescence, white arrows; scale bar 100 µm). Neointima and intima thickness was measured in cross-sections of carotid arteries by using the morphometric Cell^R software (Olympus). A significant increase in intima thickness was observed in WT animals (B, ***$p<0.001$ vs. WT control, n=6) but not in RGS5-/- mice (###$p<0.001$ vs. WT ligation, n=7). SMA-specific (red) fluorescence intensity was significantly decreased in the neointima of WT mice (C, ***$p<0.001$ vs. WT control) while increased in RGS5-deficient mice (D, ###$p<0.001$ vs. WT ligation). RGS5-specific (red) fluorescence intensity was significantly increased in neointimal SMCs (D, *$p<0.05$ vs. control; RGS5-fluorescence in the (control) media was set to 100%, green fluorescence: medial elastic fibers, scale bar 100 µm). Serial sections indicated that RGS5 RhoA-GTP were present in the same neointimal regions (E, red fluorescence).
