# Supplementary material for: RGS5 promotes arterial growth during arteriogenesis
Source: EMBO Mol Med. 2014 Jun 27;6(8):1075–89. doi: 10.15252/emmm.201403864 (PMC4154134; doi:10.15252/emmm.201403864)
Supplement: Supplementary file 15 [file emmm0006-1075-sd15.pdf]

Supplement [illegible]

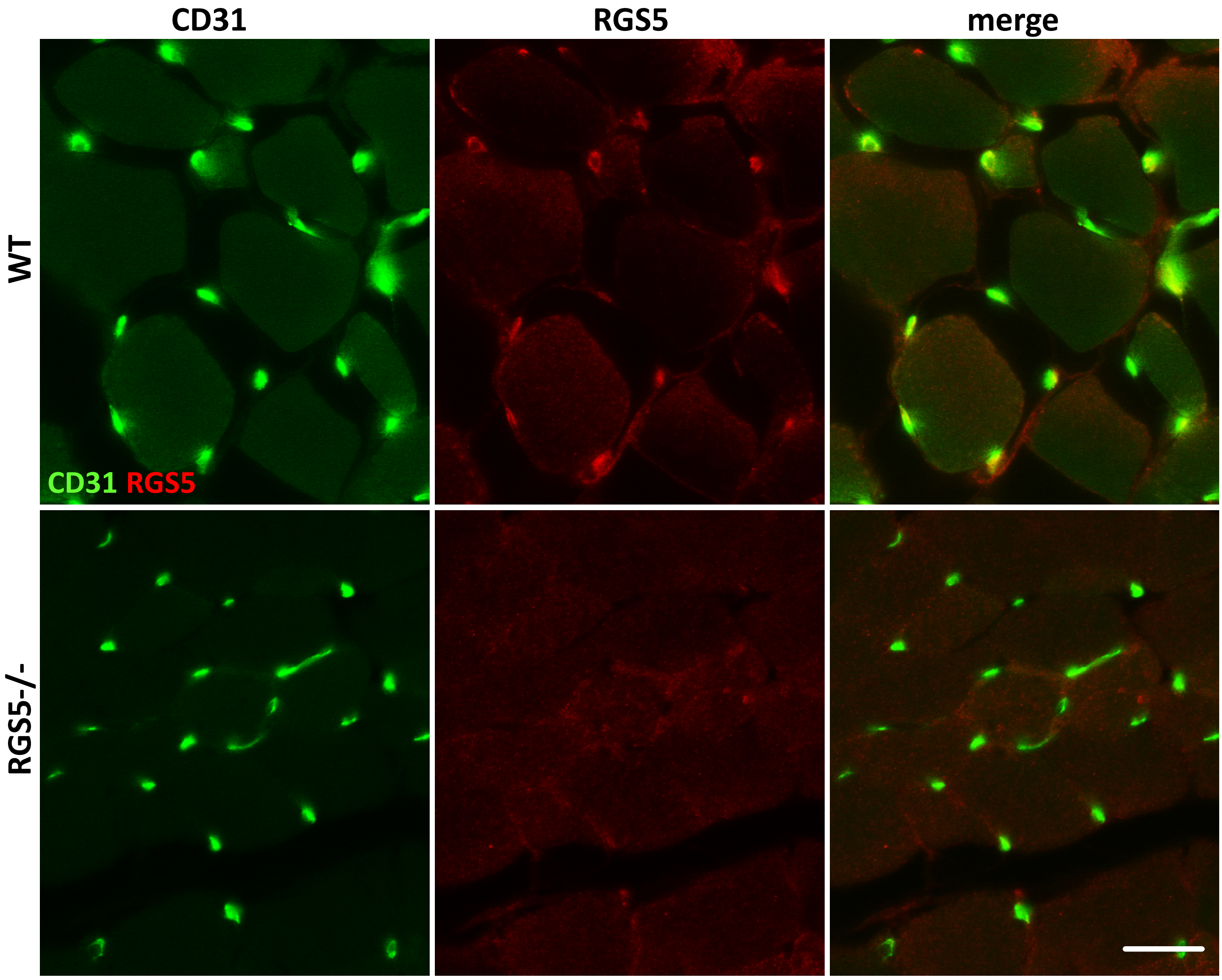

Immunofluorescence-based detection of RGS5 in wild type and RGS5-deficient mice

Pericyte-specific RGS5 was labelled on zinc-fixed and paraffin embedded muscle tissue sections of wild type and RGS5-deficient (RGS5-/-) mice (red fluorescence) by immunofluorescence techniques. Endothelial cells were visualized through detection of CD31 (green fluorescence). RGS5 was detected only in endothelial cell-associated pericytes of wild type mice which were reported to abundantly express RGS5 (scale bar: 20 µm).
