# Supplementary material for: RGS5 promotes arterial growth during arteriogenesis
Source: EMBO Mol Med. 2014 Jun 27;6(8):1075–89. doi: 10.15252/emmm.201403864 (PMC4154134; doi:10.15252/emmm.201403864)
Supplement: Supplementary file 16 [file emmm0006-1075-sd16.pdf]

EMBO
Molecular Medicine

RGS5 promotes arterial growth during arteriogenesis

Caroline Arnold, Anja Feldner, Larissa Pfisterer, Maren Hödebeck, Kerstin Troidl, Guillem Genové, Thomas Wieland, Markus Hecker and Thomas Korff

Corresponding author: Thomas Korff, University of Heidelberg

Review timeline:

Submission date:	15 January 2014
Editorial Decision:	07 February 2014
Revision received:	06 May 2014
Editorial Decision:	14 May 2014
Revision received:	22 May 2014
Accepted:	26 May 2014

Transaction Report:

(Note: With the exception of the correction of typographical or spelling errors that could be a source of ambiguity, letters and reports are not edited. The original formatting of letters and referee reports may not be reflected in this compilation.)

Editor: Céline Carret

1st Editorial Decision 07 February 2014

Thank you for the submission of your manuscript to EMBO Molecular Medicine. We have now heard back from the three referees whom we asked to evaluate your manuscript. Although the referees find the study to be of potential interest, they also raise a number of concerns that should be addressed in the next final version of the manuscript.

As you will see from the comments below, the three referees are enthusiastic about the study but do have suggestions and recommendations to further improve conclusiveness and clarity as well as increase the potential clinical implications, which is particularly important for your scope.
I will not get into experimental details, but we feel that the referees' reports are very clear and nicely detailed and we would strongly encourage you to address all issues raised as recommended.

Given these evaluations, I would like to give you the opportunity to revise your manuscript, with the understanding that the referees' concerns must be fully addressed and that acceptance of the manuscript would entail a second round of review. Please note that it is EMBO Molecular Medicine policy to allow only a single round of revision and that, as acceptance or rejection of the manuscript will depend on another round of review, your responses should be as complete as possible.

EMBO Molecular Medicine has a "scooping protection" policy, whereby similar findings that are published by others during review or revision are not a criterion for rejection. Should you decide to submit a revised version, I do ask that you get in touch after three months if you have not completed it, to update us on the status.

Please also contact us as soon as possible if similar work is published elsewhere. If other work is published we may not be able to extend the revision period beyond three months.

I look forward to seeing a revised form of your manuscript as soon as possible.

***** Reviewer's comments *****

Referee #1 (Comments on Novelty/Model System):

The authors address an important point in the understanding of the regulation of arteriogenesis. The analysis of the function of Rgs5 for balancing the synthetic and contractile phenotype of smooth muscle cells during the progression of arteriogenesis/angiogenesis is an interesting and novel mechanistic point with possible future therapeutic implications. The authors apply an overall adequate sample recovery and use an elaborated and adequate experimental approach by the use of primary SMCs of low passages and Rgs5 knockout mice. Despite that a SMC-specific Rgs5 knockout model would be useful for future studies, in particular concerning the discrepant cardiac phenotypes of the mice used in this study and used in the publication of Holobotovskyy-V. et al. 2013, Circ Res 112: 781-791. Hypothesis, results and conclusions are clearly presented.

Referee #1 (Remarks):

General

The paper deals with the hypothesis, that Rgs5 affects vascular remodeling during arteriogenesis by modulating G-protein activity.
The authors demonstrate that Rgs5 expression is elevated during arteriogenesis after the ligation of the femoral artery in the hindlimb. Furthermore the collateral growth of arterioles was impaired in mice with a genetic knockdown of Rgs5 protein in the ligation model. Studies on isolated human SMCs showed that overexpression of Rgs5 attenuated S1P- or bradykinin-induced calcium release while calcium release was increased by S1P in SMCs from Rgs5-/- mice. Moreover the potency of norepinephrine was increased in artery segments of Rgs5 deficient mice. Induced stress fibre formation was elevated in Rgs5 overexpressing smooth muscle cells accompanied by an increased RhoA activation, while the opposite was observed in a siRNA-mediated knockdown of Rgs5.
The authors conclude that: "... vascular remodelling and the switch in SMC phenotype during arteriogenesis is accompanied by and dependent on up-regulation of RGS5 in arteriolar SMCs" and that "... RGS5 shifts G q/11-PLC -MLCK-mediated constriction to G 12/13 -mediated RhoA activation and subsequent stress fibre formation in arterial SMCs".

Comments:

General

The authors address an important point by analyzing the function of Rgs5 for balancing the synthetic and contractile phenotype of smooth muscle cells during the progression of arteriogenesis/angiogenesis. The proposed shift from G q/11 to G 12/13 signaling by Rgs5 represents an interesting and novel mechanism in this context.
However, this important aspect in the manuscript is mainly supported by the analysis of semi-quantitative immunofluorescence imaging. It would be helpful to substantiate the regulation of G q/11 and G 12/13 signaling by Rgs5 in further experimental approaches and in more detail, to strengthen the evidence of the manuscript in this point (see comment 1 to 3).

© EMBO

Comment 1:

Holobotovskyy-V. et al. 2013 proposed janus activated kinase (JAK2) as a possible mediator of G q/11 to G 12/13 signaling. Hence, it would be helpful to provide further data about JAK2 activity/inhibition/expression in your experiments to elucidate a possible crosstalk of G q/11 /G 12/13 signaling.

"Strong evidence exists for G 12/13-independent G q-mediated mechanisms of RhoA activation.39-41 For instance, a recent report links the RhoA guanine exchange factor, ARHGEF1, with AngII-induced activation of RhoA signaling in VSMCs, which bypasses G 12/13. Instead, AngII activates ARHGEF1 via G q and Ca2+, involving phosphorylation through janus kinase 2.42 Interestingly, janus kinase 2 inhibition normalizes blood pressure in RGS5 KO mice and thus could provide a link between RGS5 and RhoA/Rho kinase (Online Figure XII)."

Comment 2:

More experiments should aim on the proposed shift of G q/11 to G 12/13 signaling by Rgs5 and could strengthen the manuscript by addressing some steps in the signaling cascades e.g. IP3 production, PLC activity and MLCP phosphorylation, myocardin mRNA/protein levels, JAK2 phosphorylation etc. in response to NO/stretch in Rgs5 deficient and WT cells and/or isolated arteries by additional experimental approaches such as western blotting or quantitative assays.

Comment 3:

It is not clear whether the effect shown in Figure 2C is exclusively dependent on Gαq/11 signaling since norepinephrine might activate Gαs-coupled α2-adrenergic receptors at high concentrations. This could be controlled by experiments using an α-adrenoceptor antagonist or the inclusion of a α-adrenergic antagonist to exclude indirect effects. In this context one can also argue that the S1P induced calcium release (Fig 2 A/B) could also modulated, beyond Gαq/11/Gα12/13, by other G-proteins like Gi, which are activated by S1P (e.g. Fegley et. al 2003 J. Surg. Res.). It would substantiate the experiment if other, more specific stimulations, like norepinephrine in combination which a α-adrenergic antagonist would be tested, to separate different G-protein dependent signaling cascades.

Comment 4:

The specificity of the Rgs5 antibody should be controlled in immunohistological or western blot-experiments using tissue samples from Rgs5-deficient mice in combination with a GFP antibody acting as a control in the Rgs5-Ko mouse line.

Minor Comments

Page 4, line 18: Please correct "remodellingremodelling"

Figure 1 B: Replace "flour" by "fluor" in the y-axis.

Material and Methods: Cell culture and adenoviral transduction (page 14, 2nd paragraph).
The description of cell isolation is very short. Please describe the isolation procedure in detail or refer to reference, where the isolation is described in detail.

Referee #2 (Comments on Novelty/Model System):

The ligation model is state-of-the art as regards development of collateral circulation but may limit the possibilities for molecular analysis. The supporting data using isolated cells and tissue is OK, but since the paper deals with smooth muscle phenotype regulation there is a risk that the different

models may yields inconsistent results taht need to be interpreted with caution.

Referee #2 (Remarks):

General: This interesting manuscript adds to an increasing literature describing the role of RGS5 in vascular homeostasis and injury responses. It is shown that ligation-induced arteriogenesis is accompanied by increased RGS5 signalling. Conversely, arteriogenesis was found to be inhibited in RGS5-defient mice. Increase (or presence) of RGS5 is shown to be associated with a shift of G-protein signalling from Gαq/11 towards Gα12/13-mediated responses. Evidence for this is decreased release of intracellular Ca and decreased contraction on agonist stimulation, as well as increased Rho signalling, accompanied by increased stress fibre formation, decreased G-actin contents and increased nuclear localisation of MRTF. This evidence was obtained partly by overexpression of RGS5 in human arterial cells and partly by a comparison of cells and vessels from WT and RGS5 KO mice.

The authors analyse their results in terms of a role for RGS5 in promoting a shift from a contractile to a synthetic phenotype of the VSMCs in their ligation model. This is based on decreased expression of myocardin and smooth muscle α-actin as well as increased nuclear PCNA in WT but not RGS5 KO collateral arteries. They also describe the response as a growth of the WT but not KO vessels, although only diameter increase but no other morphometric parameters are shown. Given the demonstration of increased contractile responses in the KO vessels, it can alternatively be argued that the KO vessels are more contracted and therefore limit blood perfusion. This issue may be important as previous studies have indicated that RGS5, via its negative effect on G q/11 signalling, indeed tends to limit vascular growth while increasing contractile responses. The manuscript would gain from a quantitation of vessel morphology to settle this point.

The authors repeatedly use the terms "contractile" and synthetic" phenotypes in a way that differs from their established usage in relation to the molecular mechanisms of phenotype regulation. This is most clearly shown in Fig. 6, where increased activity of the Gαq/11-PLC-IP3 pathway is implied to stimulate a contractile phenotype, whereas increase of Gα12/13-RhoA-ROCK-actin polymerisation is associated with a synthetic phenotype. Not shown in the schemes is that Gαq/11 signalling is also associated with the MEK-ERK -Elk1 proliferative pathway, as shown e.g. in the studies of Althoff et al. 2012 (referenced). This may be a semantic issue but is confusing to the reader and, more importantly, tends to lead thinking into perhaps too narrow paths. According to an overwhelming literature a shift towards more RhoA-dominated signalling would lead to an increase of smooth muscle marker expression and thus, conventionally, promotion of a "contractile" phenotype. On the other hand, in the specific context of the model used here perhaps inflammatory mediators or other factors might negatively influence the expression of myocardin and associated genes. This is however not specifically addressed in the manuscript, which instead concentrates on RhoA signalling, with ensuing apparent contradictions relative to previous literature. An additional concern is that the authors do not discuss a recent paper focusing on remodelling in RGS5 KO mice but showing discrepant results with respect to blood pressure.

Specific:

The manuscript is well written and the findings clearly described. As pointed out above more information on growth signalling and morphometry of remodelled vessels would be valuable and the discussion should be clarified. This in particular refers to the discussion of phenotype shifts and the schemes shown in Fig. 6.

Referee #3 (Remarks):

Arnold et al. report on the role of regulator of G-protein signaling 5 (RGS5) in collateral artery growth in a murine model. The authors show that RGS5 is necessary for the transition of smooth muscle cell (SMC) phenotype from contractile to synthetic and thus for arteriogenesis in-vivo. Increased Gα12/12 mediated RhoA signaling and hampered Gαq/11 mediated calcium influx is identified as an underlying mechanisms.

The manuscript elaborates on an important area in the field of vascular growth research, that is the onset of collateral artery growth, an adaptive natural process able to compensate for arterial obstruction. Understanding the mechanisms driving arteriogenesis might eventually help to stimulate this adaptive growth process in the clinical situation. Following identification of RGS5 in growing collateral arteries, the authors elucidate specific mechanisms of loss and gain of function models in-vivo and in-vitro.

There are some issues that need to be clarified in the current manuscript.

- The reported increase of RGS5 signaling in collateral arteries in-vivo on day 7 is late. The authors should also investigate RGS5 expression at earlier time points (e.g. day 3), where shear stress/wall stretch in the growing vessels are higher. At d7, much of the growth and proliferation have already taken place.

- While RGS5 knockdown leads to inrased aortic contractions upon norepinephrin stimulation, the role of RGS5 on endothelial function is not shown. Aortic ring reactions to carbachol or nitroglycerin would further improve our understand of RGS5 in vascular remodeling in the genetically modified mouse model used, and demonstrate if endothelial function also plays a role (especially as NO triggers RGS5).

- Along this line, the effect of RGS5 on endothelial cells (angiogenesis as a counterpart of arteriogenesis) should more thoroughly be investigated to round up current data. Loss and/or gain of function experiments should also be performed in endothelial cells (e.g. HUVEC) in-vitro. Capillary growth in-vivo, as investigated in the adductor muscle, should also be investigated in the lower limb, where it takes place to a stronger extent in the animal model used.

- Although SMC proliferation is essential for arteriogenesis, it is also important in neointima formation and atherosclerosis. The authors should at least discuss this potential drawback, or provide data (from the literature?). What are potential clinical applications in the long run of the data presented? Can RGS5 be stimulated e.g. in a small molecule approach?

- The mouse model used is discussed extensively in the supplemental section. However, data presented on cardiac remodeling of the two mouse models are difficult to compare as they are not both performed in the same laboratory using the same technique. Furthermore, the relation cardiac remodeling and vascular remodeling remains elusive. These data are, therefore, not helpful in the current manuscript. I suggest to shortly discuss the mouse model in the actual manuscript instead. Whether agitation of the animals during blood pressure measurements in earlier reports is debatable, as mice should have run-in measurements to get used to the procedure.

-Many data are presented in the supplement. Upregulation of RGS5 by strech or NO as presented in Supplement X is important. These data are, however, only alluded to in the discussion section, and need to be moved to the results part of the manuscript.

1st Revision - authors' response 06 May 2014

MS ID#: EMM-2014-03864

MS title: RGS5 promotes arterial growth during arteriogenesis

Point-by-point reply to reviewers' comments

Referee #1 (Comments on Novelty/Model System):

The authors address an important point in the understanding of the regulation of arteriogenesis. The analysis of the function of Rgs5 for balancing the synthetic and contractile phenotype of smooth muscle cells during the progression of arteriogenesis/angiogenesis is an interesting and novel mechanistic point with possible future therapeutic implications. The authors apply an overall adequate sample recovery and use an elaborated and adequate experimental approach by the use of primary SMCs of low passages and Rgs5 knockout mice. Despite that a SMC-specific Rgs5 knockout model would be useful for future studies, in particular concerning the discrepant cardiac phenotypes of the mice used in this study and used in the publication of Holobotovskyy-V. et al. 2013, Circ Res 112: 781-791. Hypothesis, results and conclusions are clearly presented.

Response

We appreciate these comments and agree with the reviewer that SMC-specific RGS5 deficient mice should be utilized in future studies. Corresponding mice are currently generated in collaboration with the group of Prof. Offermanns and will be available approximately within one year.

Referee #1 (Remarks):

General

The paper deals with the hypothesis, that Rgs5 affects vascular remodeling during arteriogenesis by modulating G-protein activity. The authors demonstrate that Rgs5 expression is elevated during arteriogenesis after the ligation of the femoral artery in the hindlimb. Furthermore the collateral growth of arterioles was impaired in mice with a genetic knockdown of Rgs5 protein in the ligation model. Studies on isolated human SMCs showed that overexpression of Rgs5 attenuated S1P- or bradykinin-induced calcium release while calcium release was increased by S1P in SMCs from Rgs5-/- mice. Moreover the potency of norepinephrine was increased in artery segments of Rgs5 deficient mice. Induced stress fibre formation was elevated in Rgs5 overexpressing smooth muscle cells accompanied by an increased RhoA activation, while the opposite was observed in a siRNA-mediated knockdown of Rgs5. The authors conclude that: "... vascular remodelling and the switch in SMC phenotype during arteriogenesis is accompanied by and dependent on up-regulation of RGS5 in arteriolar SMCs" and that "... RGS5 shifts Gαq/11-PLCβ-MLCK-mediated constriction to Gα12/13 -mediated RhoA activation and subsequent stress fibre formation in arterial SMCs".

The authors address an important point by analyzing the function of Rgs5 for balancing the synthetic and contractile phenotype of smooth muscle cells during the progression of arteriogenesis/angiogenesis. The proposed shift from Gαq/11 to Gα12/13 signaling by Rgs5 represents an interesting and novel mechanism in this context. However, this important

aspect in the manuscript is mainly supported by the analysis of semi-quantitative immunofluorescence imaging. It would be helpful to substantiate the regulation of Gαq/11 and Gα12/13 signaling by Rgs5 in further experimental approaches and in more detail, to strengthen the evidence of the manuscript in this point (see comment 1 to 3).

Comment 1: Holobotovskyy-V. et al. 2013 proposed janus activated kinase (JAK2) as a possible mediator of Gαq/11 to Gα12/13 signaling. Hence, it would be helpful to provide further data about JAK2 activity/inhibition/expression in your experiments to elucidate a possible crosstalk of Gαq/11 /Gα12/13 signaling. "Strong evidence exists for Gα12/13-independent Gαq-mediated mechanisms of RhoA activation. 39-41 For instance, a recent report links the RhoA guanine exchange factor, ARHGEF1, with AngII-induced activation of RhoA signaling in VSMCs, which bypasses Gα12/13. Instead, AngII activates ARHGEF1 via Gαq and Ca2+, involving phosphorylation through janus kinase 2.42 Interestingly, janus kinase 2 inhibition normalizes blood pressure in RGS5 KO mice and thus could provide a link between RGS5 and RhoA/Rho kinase (Online Figure XII)."

<u>*Response*</u>

We thank the reviewer for this comment and performed multiple Western blot analyses to investigate JAK2 expression and phosphorylation in our in vitro models by utilizing several combinations of antibodies. However, we could not observe any relevant change in JAK2 phosphorylation under the chosen conditions. In this context, we would like to stress that the data presented in the publication by Holobotovskyy et al. may not support the conclusion that JAK2 is involved in RGS5-modulated G-protein signalling (Online Figure XII). Literature inquiries underlined that AG-490 (Tyrphostin B42) is primarily an inhibitor of the EGF receptor. It is also known to inhibit Akt phosphorylation, activation of nuclear factor-κB, Stat3 and JAK2 and 3. With a concentration of 20 µM in the mouse, this inhibitor may exert a plethora of effects rather than specifically inhibiting JAK2.

Comment 2: More experiments should aim on the proposed shift of Gαq/11 to Gα12/13 signaling by Rgs5 and could strengthen the manuscript by addressing some steps in the signaling cascades e.g. IP3 production, PLC activity and MLCP phosphorylation, myocardin mRNA/protein levels, JAK2 phosphorylation etc. in response to NO/stretch in Rgs5 deficient and WT cells and/or isolated arteries by additional experimental approaches such as western blotting or quantitative assays.

<u>*Response*</u>

To address the reviewer’s suggestion and to evolve our findings, we additionally compared the IP3 production in wild type and RGS5-deficient mouse SMCs by utilizing a corresponding ELISA. While RGS5 deficiency per se generally increased the IP3 production in cultured VSMCs (see below), we did however not detect a further increase upon S1P-stimulation for technical reasons (peak IP3 production was reached within seconds as can be deduced from the calcium release – see Figure 3).

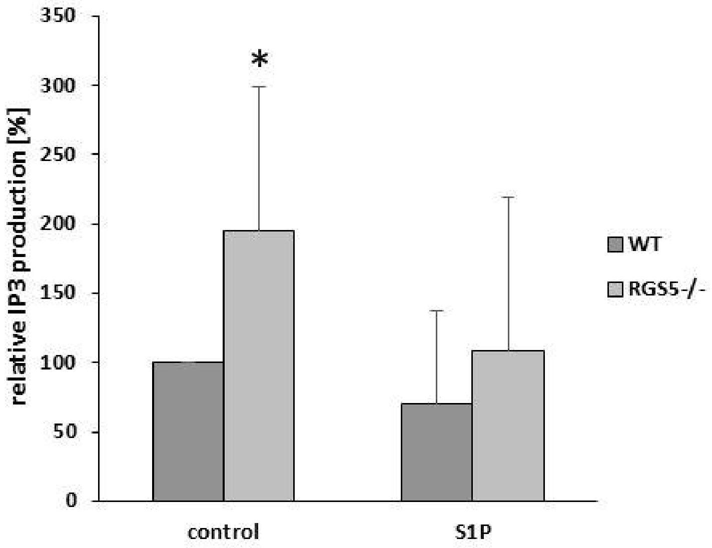

IP_3 production is increased in RGS5-deficient SMCs under baseline conditions.
Cultured vascular SMCs derived from the aortae of wild type (WT) or RGS5-deficient mice (RGS5-/-) were stimulated for 10 s with 10 µM S1P. Cells were immediately scraped off the culture dish, frozen and cellular IP_3 content was analyzed by using the mouse inositol 1,4,5,-trisphosphate (IP3) ELISA Kit (Cusabio, China) according to manufacturer's instructions. RGS5-/- SMCs showed significantly increased IP_3 levels compared to WT cells under baseline conditions.

We also analyzed the impact of RGS5 on other targets of RhoA signalling by performing an exemplary phospho kinase profiling. This approach revealed that the activity (phosphorylation) of familiar Rho-regulated protein kinases such as p38a (Dubroca et al., Hypertension, 2005; Zhang et al., J Biol. Chem., 1995), JNK (Teramoto et al., J Biol Chem., 1996), HSP27 (Dubroca et al., Hypertension, 2005) and FAK (Zhang et al., J Biol Chem., 2012) is enhanced about twofold in RGS5-overexpressing VSMCs. The activity of Rho-independet kinases such as ERK1/2 (Zuckerbraun et al., Circulation, 2003) were however not affected (these data are mentioned in the results section of the revised manuscript).
Originally, an increase in the activity of RhoA in RGS5-deficient SMCs was confirmed (i) by directly detecting and quantifying active RhoA in RGS5-deficient mice (Figure 5D), (ii) by quantifying RhoA activity (Figure 4 H and N), (iii) by monitoring RhoA-dependent stress fibre formation (Figure 4 A-G, I-M) and (iv) by utilizing RGS-Lsc - a specific inhibitor of $G\alpha_{12/13}$ signalling – to block S1P-stimulated Rho-dependent stress fiber formation (Supplement VII) in human VSMCs upon RGS5 knockdown and adenoviral RGS5 over-expression.
As an exemplary fifth approach, we additionally analysed the abundance of G-actin (globular actin) and nuclear translocation of the transcription factor MRTF-A in S1P-stimulated VSMCs (see below, see Supplement VIII). MRTF-A is bound to G-actin (globular actin) and liberated upon its incorporation into stress fibers (F-actin) – a process that is promoted by RhoA activity (e.g. Parmacek, Circ Res., 2007). In line with the other findings, the results of this additional series of experiments suggest that over-expression of RGS5 promotes nuclear translocation of MRTF-A and decreases the abundance of G-actin in S1P-stimulated cultured VSMCs. We hope that the additional findings together with our original observations alleviate the reviewer's concern.

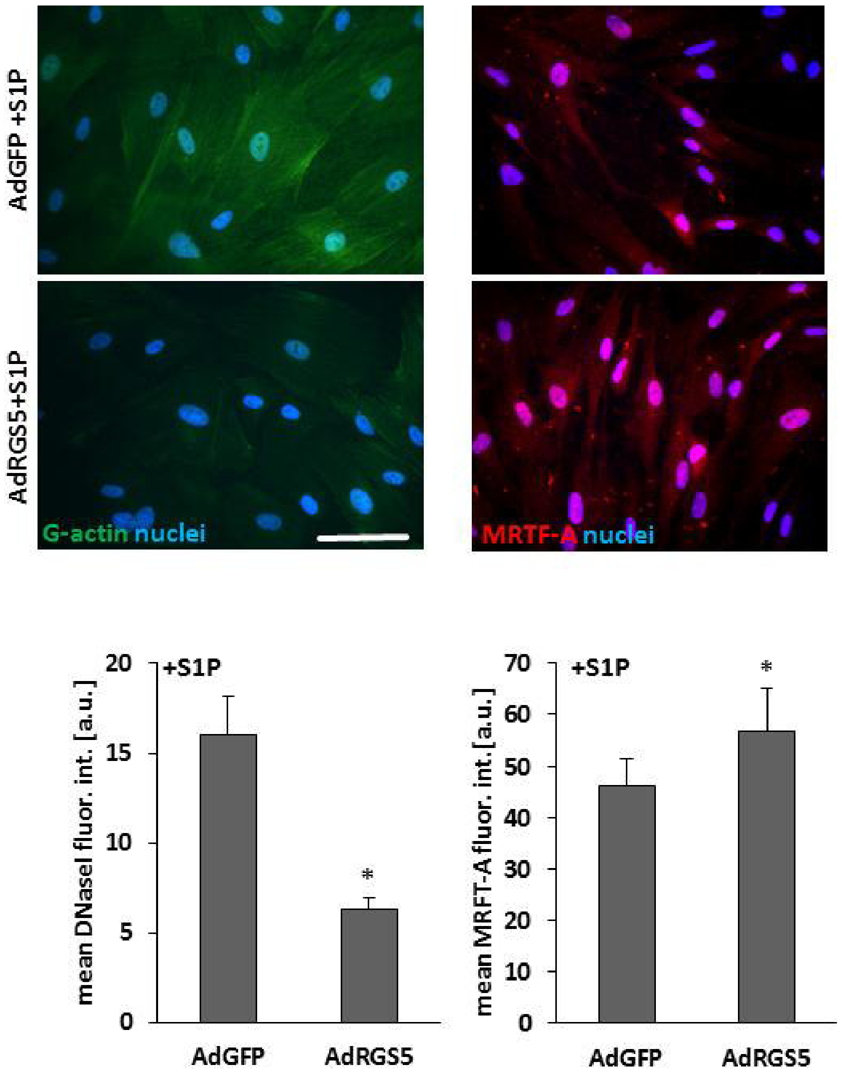

RGS5 over-expression in human arterial smooth muscle cells (HUASMCs) affects G-actin and MRTF-A localization

HUASMCs were transduced with adenoviruses expressing GFP (AdGFP) or RGS5 (AdRGS5) in serum-free medium for 48 hrs. Cells were stimulated with 10 µM S1P and processed for fluorescence detection of globular actin (monomeric G-actin binds to fluorescence-labeled DNaseI, green fluorescence) and MRTF-A (red immunofluorescence). Nuclei were visualized with DAPI (blue fluorescence). RGS5 over-expressing HUASMCs exhibited a decrease of DNaseI fluorescence intensity and more MRTF-A-positive nuclei as compared to AdGFP-transfected cells (*<p0.05 vs. AdGFP, one out of three experiments with comparable results performed in triplicates, scale bar: 100 µm).

Comment 3: It is not clear whether the effect shown in Figure 2C is exclusively dependent on Gαq/11 signaling since norepinephrine might activate Gαs-coupled β2-adrenergic receptors at high concentrations. This could be controlled by experiments using an α-adrenoceptor antagonist or the inclusion of a β-adrenergic antagonist to exclude indirect effects. In this context one can also argue that the S1P induced calcium release (Fig 2 A/B) could also modulated, beyond Gαq/11/Gα12/13, by other G-proteins like Gi, which are activated by S1P (e.g. Fegley et. al 2003 J. Surg. Res.). It would substantiate the experiment if other, more specific stimulations, like norepinephrine in combination which a β-adrenergic antagonist would be tested, to separate different G-protein dependent signaling cascades.

Response
Collectively, all our experimental results suggest that RGS5 inhibits norepinephrine and bradykinin-induced calcium mobilization from intracellular stores. To substantiate the RGS5-dependent inhibitory effect on G-protein-mediated calcium mobilization, we additionally analyzed this response in angiotensin II-treated VSMCs (see below and Supplement II of the revised manuscript). These results are in line with the publication of Holobotvskyy et al. and clearly demonstrate that RGS5 diminishes Ang II-induced calcium mobilization from intracellular stores.
We agree with the reviewer that norepinephrine may bind to other receptors at high concentrations but felt that extending this part of our study would not be linked to the main subject (arteriogenesis) of our work and were asked to improve the clinical relevance of our manuscript by analysing neointima formation in RGS5-deficient mice (see Supplement XIV). The data presented in Figure 2C (3C in the revised manuscript) was selected to demonstrate that arteries from our RGS5-deficient mice behave as has been reported in other publications (e.g. Characterization of RGS5 in regulation of G protein-coupled receptor signalling, Zhou et al., Life Sci. 2001; RGS5, RGS4, and RGS2 expression and aortic contractibility are dynamically co-regulated during aortic banding-induced hypertrophy, Wang et al., J Mol Cell Cardiol., 2008; PPARγ regulates resistance vessel tone through a mechanism involving RGS5-mediated control of protein kinase C and BKCa channel activity., Ketsawatsomkron et al., Circ. Res., 2012).
In this context, we do not understand as to how Ga_i *signaling may affect the release of calcium in S1P-stimulated VSMCs (the study of Fegley et al. investigated SMC migration) as activation of* Ga_i *would lead to a decrease in cAMP production and PKA activity which is unlikely to affect the calcium release under the chosen experimental conditions (lacking stimulation of the* Ga_s*/cAMP/PKA pathway).*

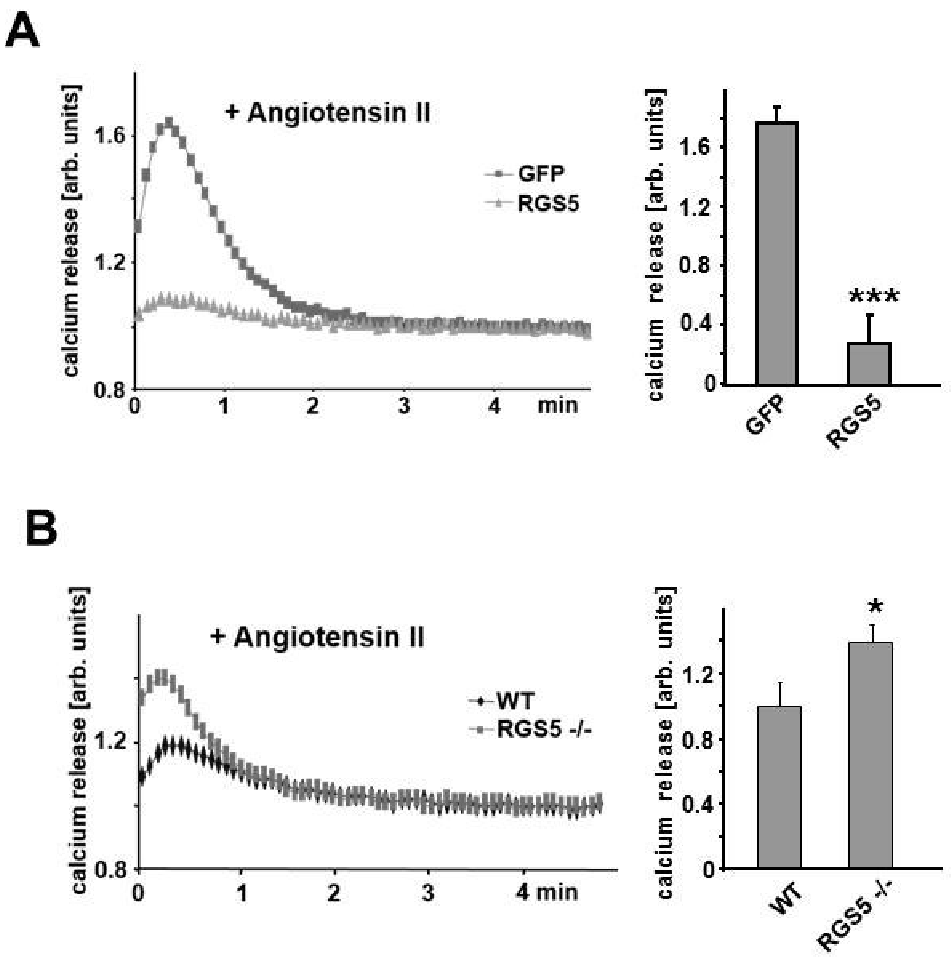

GPCR agonist-induced mobilization of intracellular calcium is modulated by RGS5
Cultured human umbilical artery SMCs were transduced with an adenoviral control (GFP) or RGS5 expression vector (RGS5), and then loaded with the calcium-sensing fluorophore Rhod-4 AM. Angiotensin II (1 µmol/L, A) elicited a rapid but transient rise in intracellular calcium in GFP-expressing cells which was virtually abrogated in cells overexpressing RGS5 (***$p<0.001$ vs. GFP-expressing cells, n=4; calcium transients were quantified by determining the area under the curve).
Cultured arterial SMCs derived from wild type (WT) or RGS5-deficient mice (RGS5-/-) were loaded with the calcium-sensing fluorophore Rhod-4 AM. Angiotensin II (1 µmol/L, B) moderately increased the intracellular calcium concentration in control SMCs. This effect was significantly reinforced in RGS5-deficient SMCs (*$p<0.05$ vs. control, n=4)

Comment 4: The specificity of the Rgs5 antibody should be controlled in immunohistological or western blot-experiments using tissue samples from Rgs5-deficient mice in combination with a GFP antibody acting as a control in the Rgs5-Ko mouse line.

Response
The specificity of the antibody has been scrutinized as shown in Supplement IV. Additionally, we verified its specificity as suggested by detecting RGS5 in pericytes where it is abundantly expressed (e.g. Bondjers et al., Am. J. Pathol., 2003; Berger et al., Blood, 2005). To this end, muscle tissue from wild type and RGS5-deficient mice was prepared in the same way as the tissues analyzed in our study.

RGS5-positive pericytes associated with capillaries (green CD31-specific fluorescence) were only detected in wild type mice (see below and Supplement XV of the revised manuscript).

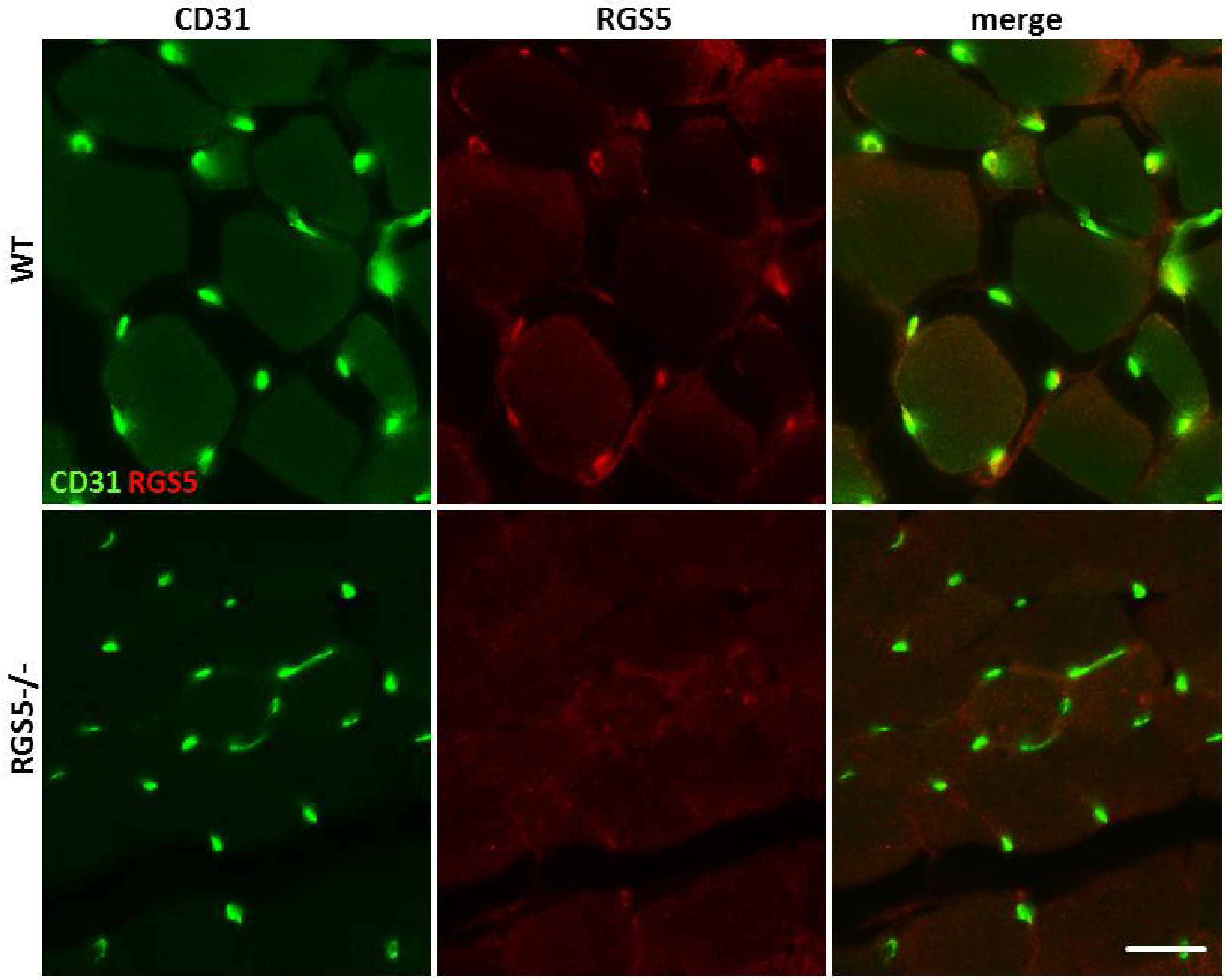

Immunofluorescence-based detection of RGS5 in wild type and RGS5-deficient mice
Pericyte-specific RGS5 was labelled on zinc-fixed and paraffin embedded muscle tissue sections of wild type and RGS5-deficient (RGS5-/-) mice (red fluorescence) by immunofluorescence techniques. Endothelial cells were visualized through detection of CD31 (green fluorescence). RGS5 was detected only in endothelial cell-associated pericytes of wild type mice which were reported to abundantly express RGS5 (scale bar: 20 µm).

Minor Comments:

Page 4, line 18: Please correct "remodellingremodelling"

Response
We apologize for this error which has been corrected.

Figure 1 B: Replace "flour" by "fluor" in the y-axis.

Response
We thank the reviewer for careful reading our manuscript and corrected this typing error.

Material and Methods: Cell culture and adenoviral transduction (page 14, 2nd paragraph). The description of cell isolation is very short. Please describe the isolation procedure in detail or refer to reference, where the isolation is described in detail.

Response
We expanded this section as suggested.

Referee #2 (Comments on Novelty/Model System):

The ligation model is state-of-the art as regards development of collateral circulation but may limit the possibilities for molecular analysis. The supporting data using isolated cells and tissue is OK, but since the paper deals with smooth muscle phenotype regulation there is a risk that the different models may yields inconsistent results taht need to be interpreted with caution.

Referee #2 (Remarks):

General: This interesting manuscript adds to an increasing literature describing the role of RGS5 in vascular homeostasis and injury responses. It is shown that ligation-induced arteriogenesis is accompanied by increased RGS5 signalling. Conversely, arteriogenesis was found to be inhibited in RGS5-defient mice. Increase (or presence) of RGS5 is shown to be associated with a shift of G-protein signalling from Gαq/11 towards Gα12/13-mediated responses. Evidence for this is decreased release of intracellular Ca and decreased contraction on agonist stimulation, as well as increased Rho signalling, accompanied by increased stress fibre formation, decreased G-actin contents and increased nuclear localisation of MRTF. This evidence was obtained partly by overexpression of RGS5 in human arterial cells and partly by a comparison of cells and vessels from WT and RGS5 KO mice.

The authors analyse their results in terms of a role for RGS5 in promoting a shift from a contractile to a synthetic phenotype of the VSMCs in their ligation model. This is based on decreased expression of myocardin and smooth muscle α-actin as well as increased nuclear PCNA in WT but not RGS5 KO collateral arteries. They also describe the response as a growth of the WT but not KO vessels, although only diameter increase but no other morphometric parameters are shown. Given the demonstration of increased contractile responses in the KO vessels, it can alternatively be argued that the KO vessels are more contracted and therefore limit blood perfusion. This issue may be important as previous studies have indicated that RGS5, via its negative effect on Gαq/11 signalling, indeed tends to limit vascular growth while increasing contractile responses. The manuscript would gain from a quantitation of vessel morphology to settle this point.

Response

We thank the reviewer for this suggestion and did additional analyses comparing the perfusion in the lower limb of wild type and RGS5-deficient mice before and after femoral artery ligation (see below). An overall increase in collateral arterial contraction would subsequently lead to a decrease in perfusion. However, we could not observe any corresponding differences right after femoral artery ligation indicating that there is no change in blood flow due to a hypercontractile response under the chosen experimental conditions. This can also be deduced from our blood pressure analyses indicating no significant differences in the diastolic pressure (representing the peripheral resistance and thus contraction of peripheral arteries) between wild type and RGS5-deficient mice.

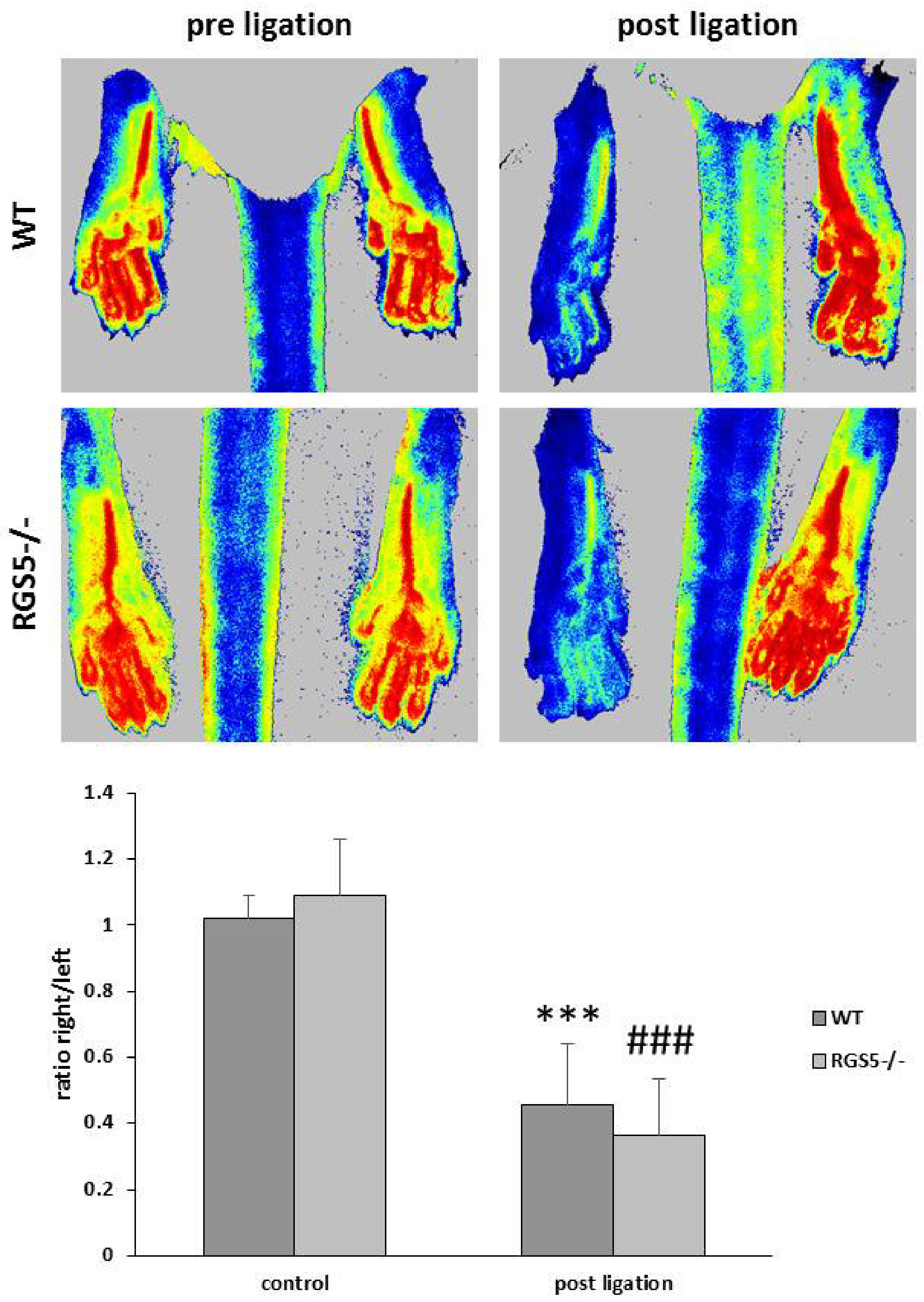

Tissue blood perfusion of footpads of WT and RGS5-/- mice.
Tissue blood perfusion was imaged using the PeriCam PSI System (Perimed) before and after ligation of the left femoral artery just distal to the origin of the depp femoral artery in wild type (WT) and RGS5-deficient (RGS5-/-) mice.. No differences in tissue perfusion were observed in both groups before ligation and perfusion decreased to the same extent in WT and RGS5-/- mice directly after ligation.

In addition, we determined the wall thickness as another morphologic parameter of remodelling collateral arterioles as suggested (see below). While no difference in wall thickness was observed under control conditions, this parameter was increased only in collaterals of wild type mice 7 days post femoral artery occlusion. The data has been included in Figure 5 (C) of the revised manuscript.

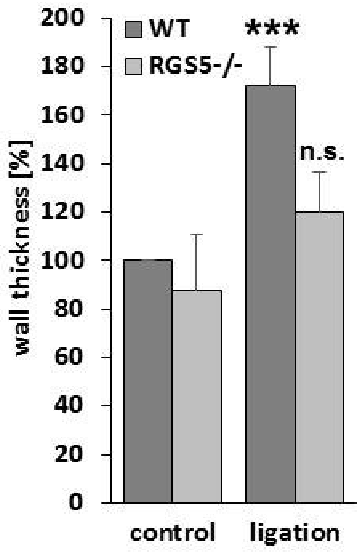

RGS5 deficiency inhibits growth of collateral arterioles
Arteriogenic remodelling of collateral arterioles in the mouse hindlimb was analysed 7 days post ligation of the femoral artery. Wall thickness is not increased during arteriogenesis in RGS5-deficient mice (C, ***p<0.001 vs. control (WT) and not significant (n.s.) vs. control (RGS5-/-), n=5).

The authors repeatedly use the terms "contractile" and synthetic" phenotypes in a way that differs from their established usage in relation to the molecular mechanisms of phenotype regulation. This is most clearly shown in Fig. 6, where increased activity of the Gαq/11-PLC-IP3 pathway is implied to stimulate a contractile phenotype, whereas increase of Gα12/13-RhoA-ROCK-actin polymerisation is associated with a synthetic phenotype. Not shown in the schemes is that Gαq/11 signalling is also associated with the MEK-ERK -Elk1 proliferative pathway, as shown e.g. in the studies of Althoff et al. 2012 (referenced). This may be a semantic issue but is confusing to the reader and, more importantly, tends to lead thinking into perhaps too narrow paths. According to an overwhelming literature a shift towards more RhoA-dominated signalling would lead to an increase of smooth muscle marker expression and thus, conventionally, promotion of a "contractile" phenotype.

Response
We appreciate this remark. We agree that our wording may confuse the readers as “contractile response” and “contractile phenotype” do not properly distinguish between signalling processes controlling either an acute SMC contraction or phenotype changes in SMCs. We have changed the wording in the revised manuscript to more precisely distinguish between “contracting”, “resting” and “activated” SMCs and have adapted the discussion accordingly.
From our point of view, SMCs respond context-dependently to distinct stimuli. For instance, acute norepinephrine-induced Ga$_{q/11}$ signalling does not usually induce SMC proliferation on its own despite being linked to the activation of ERK1/2. However, its chronic failure may prohibit phenotypic changes of SMCs during vascular remodelling as has nicely been demonstrated by Althoff et al. Along these lines, RhoA is a well-established determinant of SMC marker gene expression. It is nevertheless rate-limiting for vascular remodelling processes such as arteriogenesis and neointima formation requiring activation of SMCs (Shibata et al., Circulation 103:284-9, 2001; Eitenmuller et al., Circ Res 99:656-662,

2006; Troidl et al. ATVB 29:2093-2101, 2009). Thus, RhoA signalling appears to be both, a regulator of SMC contraction and activation: On the one hand, it is in fact involved in SMC contraction as it inhibits MLCP activity. On the other hand, RhoA may act context-dependently to control adaptive changes in the cytoskeleton (stress fibre formation) which is a prerequisite for cells to adequately respond to changes in biomechanical forces (e.g. Zhao et al, ATVB, 1995 or work from our laboratory: Wójtowicz et al., *Circ. Res., 2010; Numaguchi et al., Circ Res., 1999; Liu et al., Circ Res., 2007). Consequently, Rho activity is rate-limiting for the onset of arteriogenesis and therefore rate-limiting for adequate SMC phenotype changes. Considering this, we claim that RGS5 controls the SMC phenotype in the context of arteriogenesis by indirectly enhancing the activity of RhoA. Against this background and by considering arteriogenesis as a biomechanically triggered vascular remodelling process Rho signalling enables activation and proliferation of SMCs rather than expression of marker genes maintaining their quiescence under these conditions.*
Interestingly, we revealed that loss of RGS5 also inhibits neointima formation (see comments to reviewer 3 and Supplement XIV of the revised manuscript) which is known to be a Rho-dependent remodelling process (e.g. Shibata et al., Circulation, 2001) and has recently been associated with an increase in the abundance and activity of the Rho signalling-responsive transcription factor MRTF-A (Minami et al., EMBO J., 2012). Likewise, Althoff et al. report an increase in RhoA activity during neointima formation in wild type mice (J Exp Med., 2012; Figure 3A). However, the exact role of RGS5 in this context needs to be further explored as it may affect multiple signalling pathways as can be deduced from our phospho kinase array data (see responses to Reviewer 1).

On the other hand, in the specific context of the model used here perhaps inflammatory mediators or other factors might negatively influence the expression of myocardin and associated genes. This is however not specifically addressed in the manuscript, which instead concentrates on RhoA signalling, with ensuing apparent contradictions relative to previous literature.

Response
Myocardin has been reported as a critical transcriptional co-activator that controls the expression of gene products such as calponin or smooth muscle myosin heavy chain which are crucial for SMCs to develop a contractile phenotype. Loss of myocardin is accompanied by an increase in SMC proliferation and a decrease in the expression of its target genes (see below; Pfisterer et al., Cardiovascular Research, 2012) – characteristics of a synthetic or activated SMC phenotype. As proliferation, migration and synthetic activity is a hallmark of SMCs involved in vascular remodelling processes such as arteriogenesis, a decrease in myocardin abundance may indicate a limited capacity of SMCs to promote the expression of contractile gene products (Pfisterer et al., Cardiovascular Research, 2012).

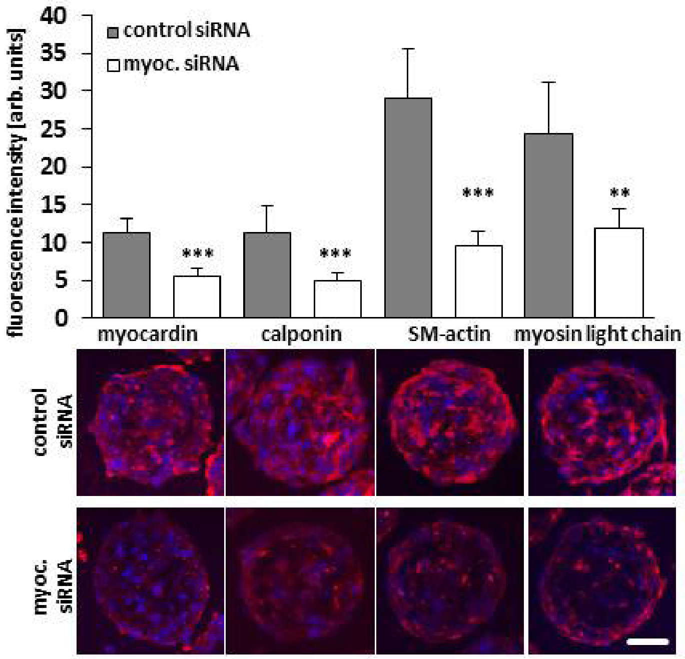

Analyses of the effects of myocardin silencing on the expression of SMC marker proteins

To culture SMCs in an organotypic manner, three-dimensional spheroids were generated from siRNA pre-treated smooth muscle cells which were fixed, embedded in paraffin and processed for sectioning. Immunofluorescence-based (red) detection of myocardin, calponin, smooth-muscle (SM)-actin and myosin light chain revealed a significant decrease in the abundance of these proteins (B, ***$p<0.001$, **$p<0.01$ vs. control siRNA). Shown are the means±SD of one representative of four independent experiments with comparable results summarizing the fluorescence intensity of 5-10 spheroids. The bottom panel shows one representative spheroid per condition (scale bar: 50 µm).

As we already published a manuscript about the biomechanical control of myocardin in vascular SMCs, those experiments were not repeated in the context of this manuscript. So far, we did not observe an effect of pro-inflammatory mediators such as MCP-1 on myocardin expression or stability in arterial SMCs (data not shown). However, biomechanical stretch - an important determinant of arteriogenesis – diminishes its abundance in SMCs (Pfisterer et al., Cardiovascular Research, 2012).

To directly address the reviewers concern, we additionally showed that macrophage infiltration of growing collaterals as a hallmark of arteriogenesis and prototypic pro-inflammatory response is impaired in RGS5-deficient mice (see below). This was hardly surprising as the activity of the pro-inflammatory transcription factor activator protein 1 (AP-1) is controlled by RhoA-mediated signalling in biomechanically stimulated SMCs (Mohamed et al., Am J Physiol Lung Cell Mol Physiol., 2010; Cattaruzza et al., J Biol Chem., 2001). Likewise, blockade of RhoA signalling prevents vascular inflammation (Matsumoto et al., ATVB, 2004; e.g. diminished MCP-1 expression and macrophage infiltration). Considering that AP-1 is rate-limiting for pro-inflammatory responses of SMCs during arteriogenesis (Demicheva et al., Circ. Res., 2008), diminished RhoA activity may thus interfere with collateral growth on multiple levels.

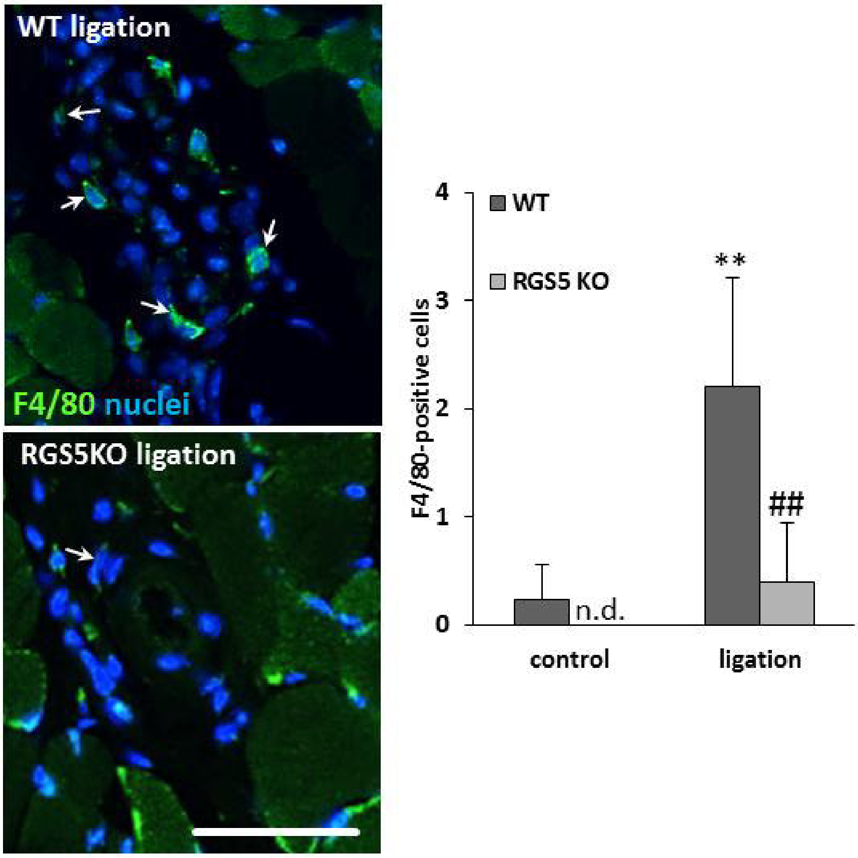

Comparison of arteriogenesis-associated macrophage recruitment in WT and RGS5-/- mice

Seven days after ligation of the femoral artery the number of macrophages (F4/80-positive cells, arrows, green fluorescence) in the media/adventitia of collateral arterioles was determined. While arteriogenic remodeling in wild type (WT) is associated with a significant increase in macrophage infiltration (**$p<0.01$ vs. control WT, n=5), decreased numbers of collateral-associated macrophages were detected in RGS5-deficient (RGS5KO; ##$p<0.01$ vs. WT ligation) mice (scale bar: 40 µm).

An additional concern is that the authors do not discuss a recent paper focusing on remodelling in RGS5 KO mice but showing discrepant results with respect to blood pressure.

<u>*Response*</u>

The discrepant findings are now mentioned in the discussion of the revised manuscript. However, the reason why we were somewhat hesitant to extensively discuss the data by Holobotovskyy et al. has been explained in the "Supplementary information for reviewers" that was provided with our original submission (see below). As we detected a cardiac phenotype of the mice utilized throughout that study (which may affect blood pressure), we repeated all of our experiments with RGS5-deficient mice that we had obtained from the group of Dr. Christer Betsholtz from Stockholm/Sweden. These mice do not suffer from a cardiac phenotype as has been confirmed by echocardiographic analyses (see below).

<u>***Comment on the publication of Holobotovskyy-V. et al., Circ Res 2013***</u>

Another study has shown that knockout of RGS5 leads to hypertension (Holobotovskyy-V. et al., Circ. Res. 2013) which is in contrast to the presented data. We are hesitant to discuss this work as the mice

utilized for the study of Holobotovskyy-V. et al. obviously suffer from a cardiac phenotype which was not considered.

We originally collaborated with Dr. Ruth Ganss and colleagues from Perth/Australia and were using her RGS5-deficient mice for our experiments. With respect to arteriogenesis, these mice displayed the same phenotype as has been described in our manuscript (see below, A). However, we could not verify the increase in blood pressure as has been described by her group (see below, B).

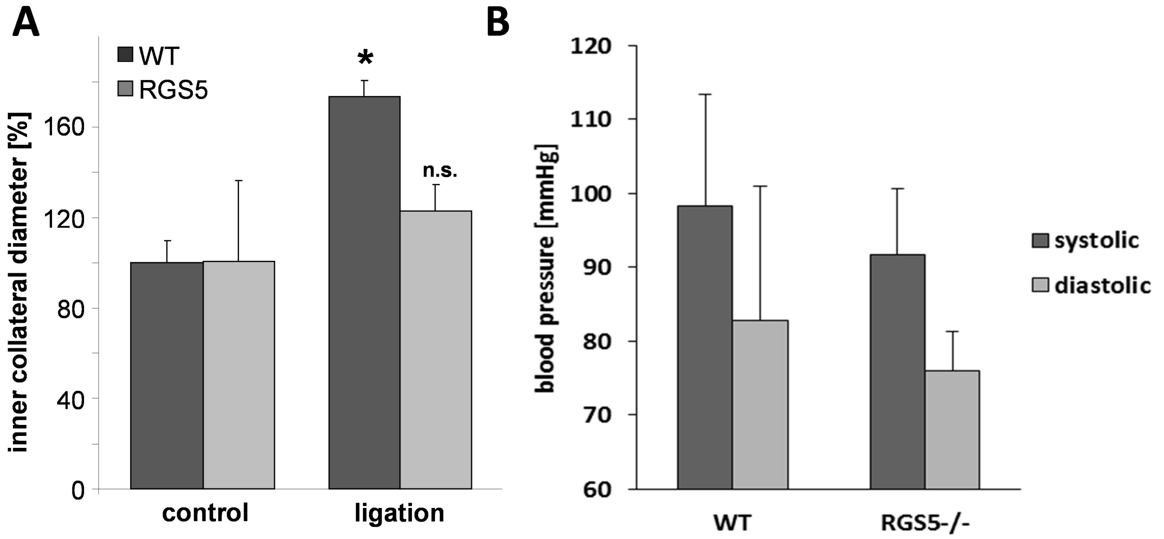

(A) RGS5 deficiency inhibits growth of collateral arterioles. Arteriogenic remodeling of collateral arterioles in the mouse hindlimb was analyzed 7 days post ligation of the femoral artery. Growth of the remodeling arterioles (arrows) is significantly increased during this period in wild type mice but not in RGS5-deficient mice (*p<0.05 vs. control, n.s. – not significant vs. control, n=4-6).

(B) Comparison of blood pressure values in wild type (WT) and *rgs5*$^{-/-}$ mice. *Rgs5*$^{-/-}$ mice exhibited a slightly but not significantly lower systolic and diastolic blood pressure than WT animals as was evidenced by repeated tail-cuff measurements (LE 5001 pressure meter, Panlab software). The graph shows mean systolic (dark grey) and mean diastolic (light grey) values + SD from 6 animals per group.

Moreover, our original data indicated that these mice show impaired cardiac parameters (see below) which may alter responses of the circulatory system.

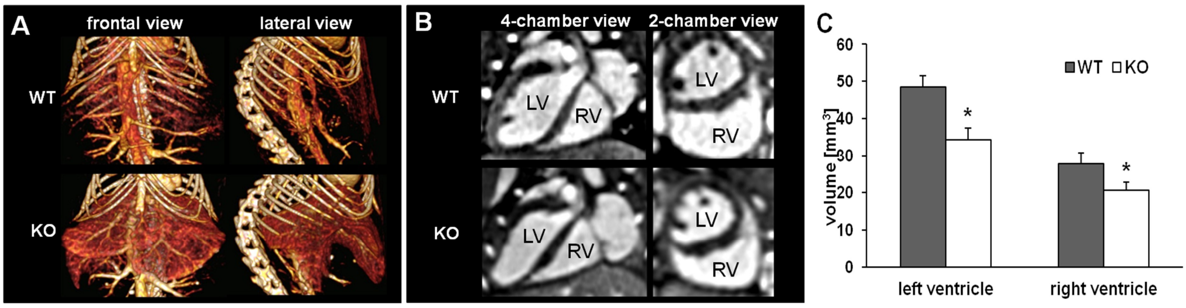

CT and VCT analyses of RGS5-deficient hearts

Non-invasive imaging results of wildtype (WT) and *rgs5* knockout (KO) mice from volumetric computed tomography (VCT) which was performed right after pressure overload induced by the injection of 600 µL contrast agent in the tail vein. 3D surface reconstructions of the mouse vasculature in frontal and lateral views (post mortem analysis) reveal an increase in intravascular volume in the liver of KO mice by venous congestion indicative for an insufficient heart function under these conditions (A, representative images; identical windowing levels for all images). Representative images of 4-chamber and 2-chamber views of the heart are shown below (B, LV, left ventricle; RV, right ventricle). Quantitative data from VCT imaging of the ventricle volumes indicate reduced ventricle volumes in rgs5-/- animals as compared to WT mice (C, *p<0.05, Wilcoxon rank sum test, n=5-6; error bars represent standard error).

To exclude a possible impact of this cardiac phenotype on our results, we repeated all of our experiments with RGS5-deficient mice that we had obtained from the group of Dr. Christer Betsholtz from Stockholm/Sweden which have a slightly different genetic background:

- *RGS5 knockout mice from Dr. Ganss were generated on a mixed genetic background (S129 x C57BL/6) and bred on a C3HeBFe or C57BL/6 background and backcrossed for more than 30 generations.*
- *RGS5 knockout mice from Dr. Betsholtz were generated on a mixed genetic background (S129 x C57BL/6) and bred on an ICR or C57BL/6 background and backcrossed to C57BL/6 for at least 7 generations.*

The mice utilized throughout our study do not suffer from a cardiac phenotype as has been scrutinized in the beginning of this study by rigorous echocardiographic analyses (see below). As both strains showed comparable results with respect to arteriogenesis, we are confident that our observations are not dependent on the genetic background or an impaired heart function.

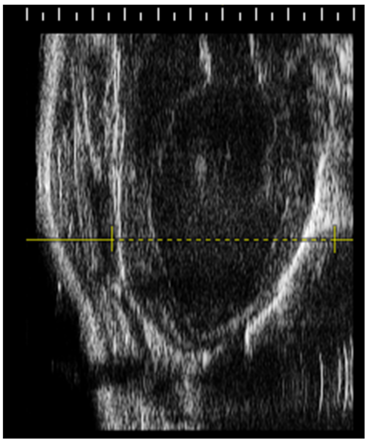

	Wild type	RGS5-/-
HW/BW	5.6 ± 0.4	6.5 ± 0.8
EF	65.4 ± 7.5 %	69.3 ± 6.6 %
FS	35.7 ± 5.4 %	38.5 ± 5.0 %
LVPW	0.8 ± 0.2 mm	0.9 ± 0.2 mm
CO	20.9 ± 4 ml/min	21.8 ± 3.2 ml/min

Echocardiographic data of RGS5-deficient and wild type mice
Heart weight-body weight ratio (HW/BW), ejection fraction (EF), fractional shortening (FS), left ventricle posterior wall thickness (LVPW), cardiac output (CO). Significant differences were not observed when comparing RGS5-/- and wild type values (n=7).

Specific: The manuscript is well written and the findings clearly described. As pointed out above more information on growth signalling and morphometry of remodelled vessels would be valuable and the discussion should be clarified. This in particular refers to the discussion of phenotype shifts and the schemes shown in Fig. 6.

Response
We thank the reviewer for this comment. We have included additional morphometric analyses of growing collaterals. Moreover, we improved the manuscript by adding experiments analysing RGS5 expression at the earlier time points of collateral remodelling (see responses to reviewer 3). Finally, we clarified the discussion and adapted the scheme shown in Figure 7 accordingly and hope that this alleviates the reviewer's concerns.

Referee #3 (Remarks):

Arnold et al. report on the role of regulator of G-protein signaling 5 (RGS5) in collateral artery growth in a murine model. The authors show that RGS5 is necessary for the transition of smooth muscle cell (SMC) phenotype from contractile to synthetic and thus for arteriogenesis in-vivo. Increased G12/12 mediated RhoA signaling and hampered Gq/11 mediated calcium influx is identified as an underlying mechanisms.

The manuscript elaborates on an important area in the field of vascular growth research, that is the onset of collateral artery growth, an adaptive natural process able to compensate for arterial obstruction. Understanding the mechanisms driving arteriogenesis might eventually help to stimulate this adaptive growth process in the clinical situation. Following identification of RGS5 in growing collateral arteries, the authors elucidate specific mechanisms of loss and gain of function models in-vivo and in-vitro.

There are some issues that need to be clarified in the current manuscript.

- The reported increase of RGS5 signaling in collateral arteries in-vivo on day 7 is late. The authors should also investigate RGS5 expression at earlier time points (e.g. day 3), where shear stress/wall stretch in the growing vessels are higher. At d7, much of the growth and proliferation have already taken place.

<u>*Response*</u>

We agree with the reviewer that the expression pattern of SMCs may change throughout early and late phases of arteriogenesis. Based on our experience and underlined by the presented results, a robust collateral artery growth is observed 7 days post induction of arteriogenesis which gradually weakens within 14 days. To convince the reviewer that arteriogenesis is in fact accompanied by an increase in RGS5 expression, we verified the increase in RGS5 abundance in SMCs of growing collaterals three days after ligation of the femoral artery (see below and Supplement I of the revised manuscript).

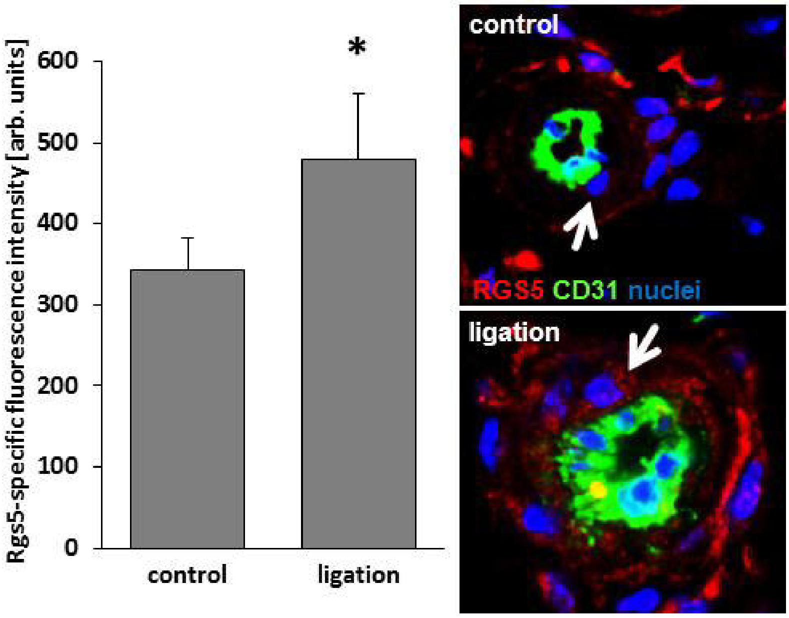

RGS5 protein abundance is increased in remodelling collateral arterioles (3d)
Arteriogenic remodelling of collateral arterioles in the mouse hindlimb was analysed 3 days post ligation of the femoral artery (early remodeling phase). Quantification of RGS5-specific fluorescence intensity (red fluorescence) in the SMCs of these arterioles (arrow) revealed a moderate increase in RGS5 protein abundance (*$p<0.05$ vs. control, n=5, analysing up to 3 collaterals per animal).

In addition, we reanalysed an earlier experiment which is founded on two different mouse lines (BPL and BPH) based on the same genetic background. The BPL mice have a lower blood pressure and show only minimal arteriogenic responses as compared to BPH mice (see below). This gave us the opportunity to analyse RGS5 expression in growing collaterals within the same experimental setup but different levels of arteriogenesis. Quantitative real-time analyses of mRNA samples isolated from growing collateral arterioles suggest that the level of RGS5 expression depends on the level of arteriogenesis (see below). We hope that this data fully alleviates the reviewer's concern.

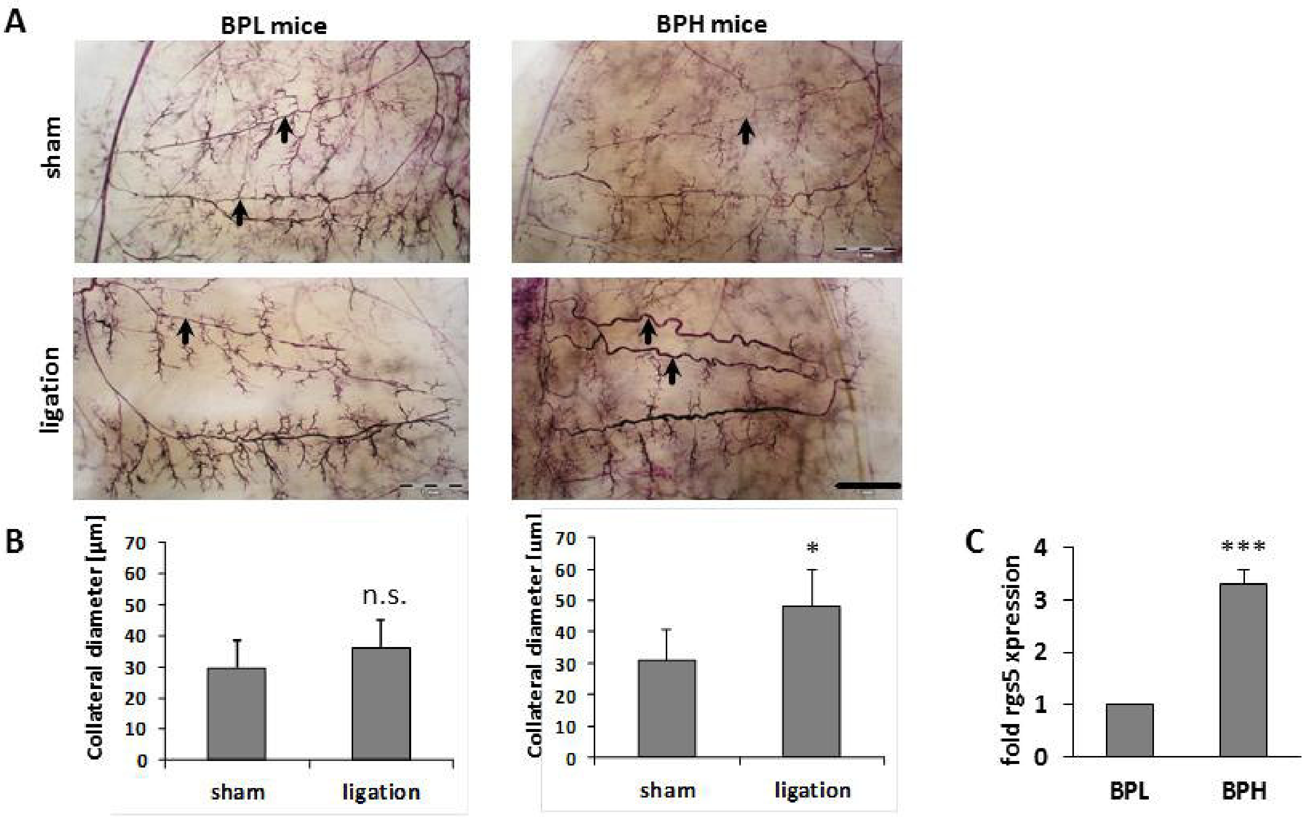

Comparison of arteriogenesis in BPL and BPH mice.
Seven days after ligation of the femoral artery, the inner diameter of collateral arterioles (A: black arrows, representative images) was determined by utilizing the morphometric analysis software Cell^R (Olympus). Panel B depicts the mean collateral diameter + SD obtained by analysing 4 to 5 mice for each condition. BPH mice exhibited a significant increase in collateral diameter (B, *p<0.05 vs. sham) whereas BPH mice hardly showed collateral growth (B, n.s. – not significant, scale bar 1 mm). PCR analyses of growing collaterals (7 days after ligation) isolated from these tissues showed that RGS5 mRNA levels were significantly increased in BPH mice compared to BPL mice (C, ***p<0.001 vs. BPL, n=4-5; BPL values were set to 1).

- While RGS5 knockdown leads to inrased aortic contractions upon norepinephrin stimulation, the role of RGS5 on endothelial function is not shown. Aortic ring reactions to carbachol or nitroglycerin would further improve our understand of RGS5 in vascular remodeling in the genetically modified mouse model used, and demonstrate if endothelial function also plays a role (especially as NO triggers RGS5).

Response
We are unsure to which experiment the reviewer is referring to as no aortic ring contraction experiments were performed. Although we do not fully exclude an endothelial cell-specific function of RGS5 during arteriogenesis, we respectfully disagree that carbachol- or nitroglycerin-induced dilation

of aortic rings might help to further understand its role in collateral arteriolar remodelling. Generally, the mice utilized within our study do not appear to suffer from relevant endothelial dysfunction as their vascular system develops normally and neither developmental nor tumor angiogenesis is altered (Maya et al., Mol. Cell. Biol. 28:2324-31, 2008). Moreover, we only observe an alteration in arterial contraction but not dilation. To fully explore the specific role of RGS5 in endothelial and smooth muscle cells, its cell-specific knockdown appears to be a prerequisite. Corresponding 'floxed mice' are currently generated in collaboration with the group of Prof. Offermanns and will be available approximately within one year.

- Along this line, the effect of RGS5 on endothelial cells (angiogenesis as a counterpart of arteriogenesis) should more thoroughly be investigated to round up current data. Loss and/or gain of function experiments should also be performed in endothelial cells (e.g. HUVEC) in-vitro. Capillary growth in-vivo, as investigated in the adductor muscle, should also be investigated in the lower limb, where it takes place to a stronger extent in the animal model used.

Response

We performed additional analyses on capillary growth by determining the capillary density in hypoxic calf muscles (lower limb) 3 days post femoral artery ligation as suggested. We could not observe any differences when comparing angiogenesis in wild type and RGS5-deficient mice (see below). This data has been included in the revised version of our manuscript as Supplement X. These findings are in line with the study originally characterizing the mice utilized in our study (Maya et al., Mol. Cell. Biol. 28:2324-31, 2008).

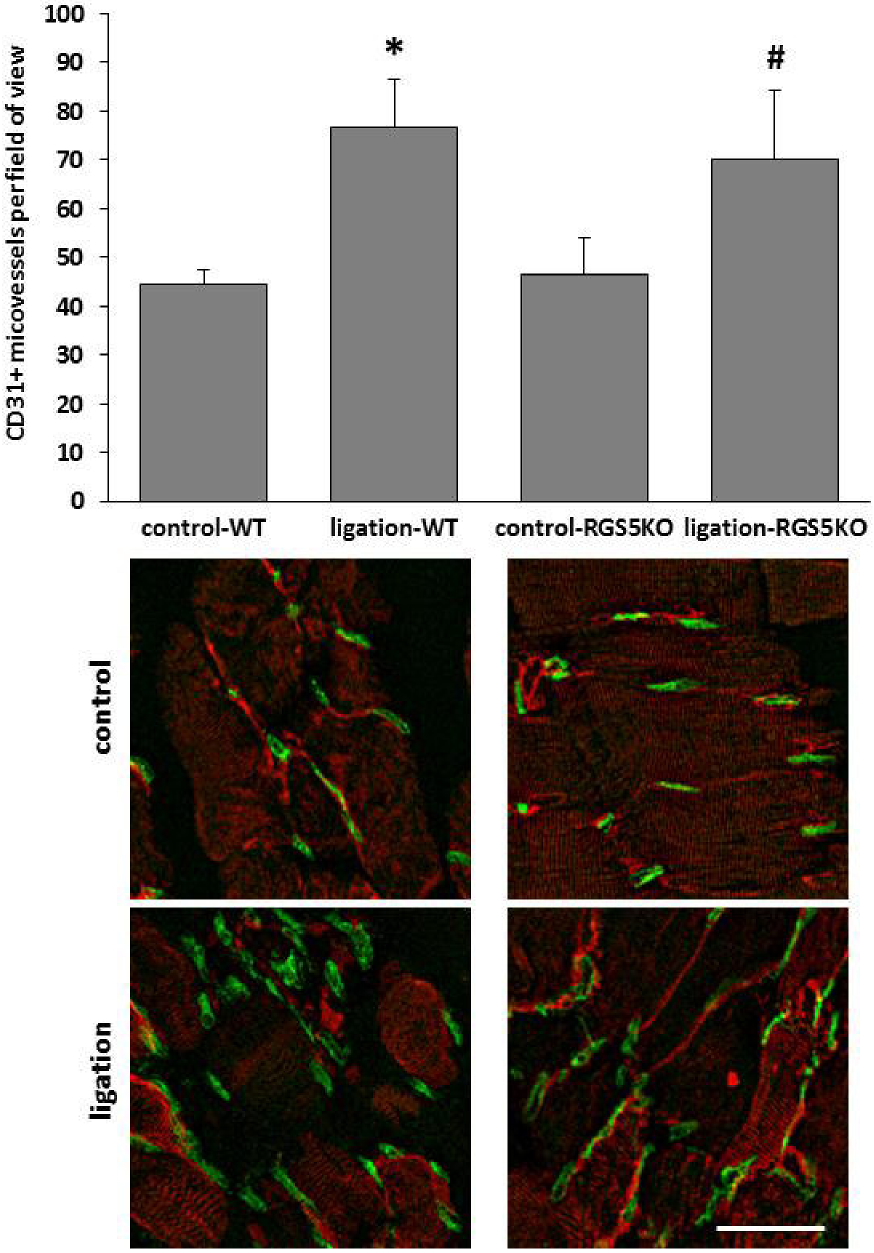

Comparison of the capillary density in the ischemic calf muscle of WT and RGS5-/- mice

Three days after induction of ischemia through ligating the femoral artery, calf muscle tissue was dissected, fixed in zinc fixative, embedded in paraffin and sectioned. The graph shows the mean number of capillaries +SD per microscopic field of view (MFV) in the calf muscle tissue of WT and RGS5-deficient (RGS5KO) mice (n=5, determining CD31-positive capillaries in at least two MFV per animal and condition). Ligation-induced ischemia stimulated an increase in capillary density in muscle tissues of both wild type (WT) and RGS5KO mice (*$p<0.05$ vs. WT control, #$p<0.05$ vs. RGS5KO control). Representative images show CD31-positive endothelial cells (green fluorescence, scale bar: 50 µm).

- Although SMC proliferation is essential for arteriogenesis, it is also important in neointima formation and atherosclerosis. The authors should at least discuss this potential drawback, or provide data (from the literature?). What are potential clinical applications in the long run of the data presented? Can RGS5 be stimulated e.g. in a small molecule approach?

<u>*Response*</u>

We thank the reviewer for this remark and have expanded our experiments as suggested. We applied another in vivo model which triggers neointima formation by ligating the carotid artery. This remodelling process is associated with and dependent on an increase in RhoA activity (e.g. Shibata et al., Circulation, 2001). In line with our original findings, SMC proliferation and neointima formation is impaired in RGS5-deficient mice (see below and Supplement XIV of the revised manuscript). The discussion has been adapted accordingly and additionally addresses small molecule approaches (e.g. Romas et al. Mol Pharmacol. 71:169-75, 2007) interfering with the function of RGS proteins and

possible clinical drawbacks of those therapies (e.g. Ketsawatsomkron et al. Circ Res., 111:1446-58, 2012).

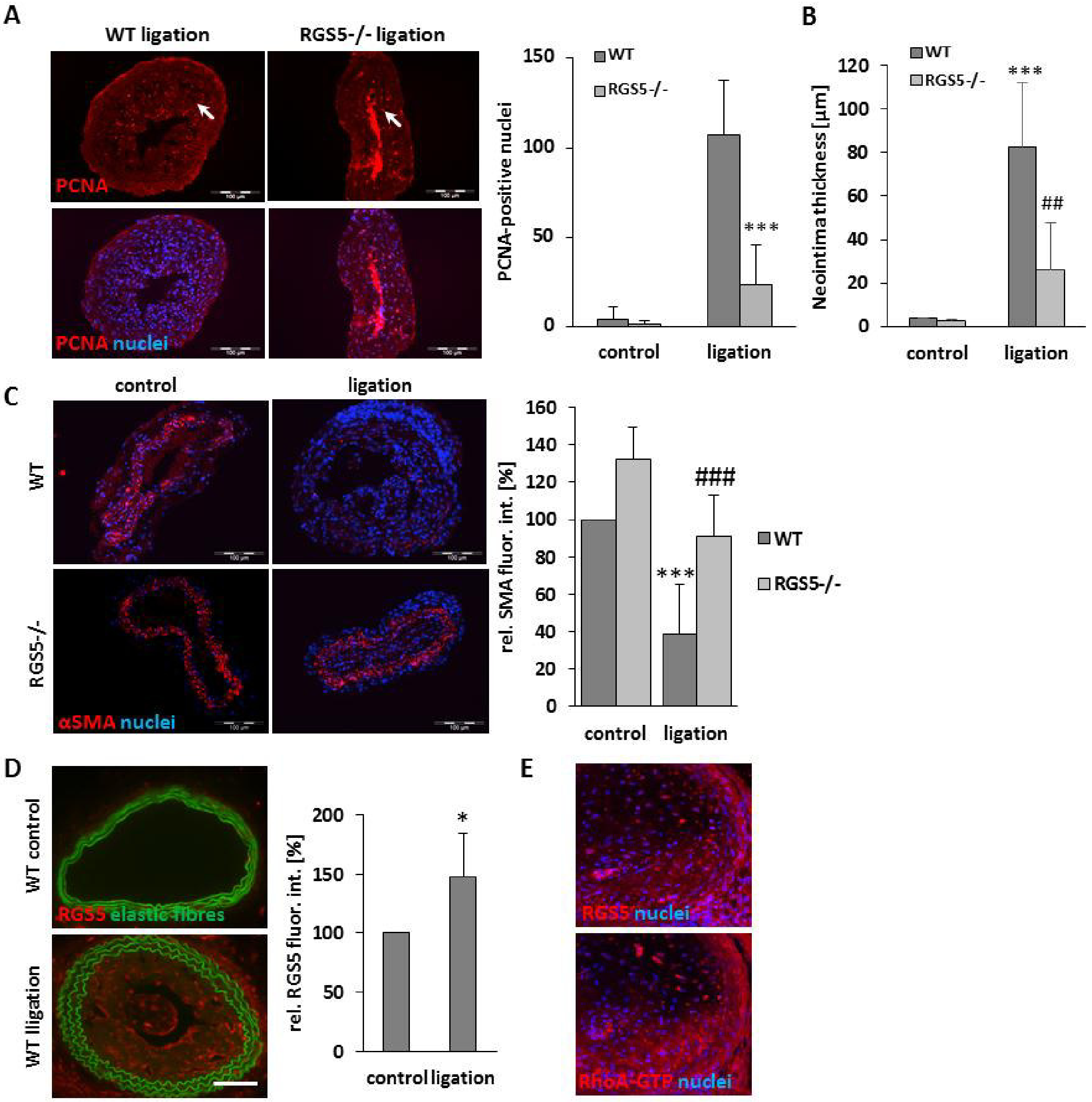

Comparison of neointima formation in wild type (WT) and RGS5-/- mice

The left common carotid artery was occulded proximal to the bifurcation of the external and internal carotid artery to induce flow cessation in the distal part of that artery. After 4 weeks carotids were harvested, fixed in zinc fixative, embedded in paraffin and sectioned. Proliferation of neointimal cells was significantly decreased in RGS5-deficient mice as evidenced by staining of the proliferation marker PCNA (A, ***$p<0.001$ vs. WT ligation; PCNA - red fluorescence, white arrows; scale bar 100 µm). Neointima and intima thickness was measured in cross-sections of carotid arteries by using the morphometric Cell^R software (Olympus). A significant increase in intima thickness was observed in WT animals (B, ***$p<0.001$ vs. WT control, n=6) but not in RGS5-/- mice (###$p<0.001$ vs. WT ligation, n=7). SMA-specific (red) fluorescence intensity was significantly decreased in the neointima of WT mice (C, ***$p<0.001$ vs. WT control) while increased in RGS5-deficient mice (D, ###$p<0.001$ vs. WT ligation). RGS5-specific (red) fluorescence intensity was significantly increased in neointimal SMCs (D, *$p<0.05$ vs. control; RGS5-fluorescence in the (control) media was set to 100%, green fluorescence: medial elastic fibers, scale bar 100 µm). Serial sections indicated that RGS5 RhoA-GTP were present in the same neointimal regions (E, red fluorescence).

- The mouse model used is discussed extensively in the supplemental section. However, data presented on cardiac remodeling of the two mouse models are difficult to compare as they are not both performed in the same laboratory using the same technique. Furthermore, the relation cardiac remodeling and vascular remodeling remains elusive. These data are, therefore, not helpful in the current manuscript. I suggest to shortly discuss the mouse model in the actual manuscript instead. Whether agitation of the animals during blood pressure measurements in earlier reports is debatable, as mice should have run-in measurements to get used to the procedure.

Response
We agree with the reviewer. The information about the mouse model was not intended to be shown in our final manuscript and has been introduced to point out our concerns regarding the manuscript of Holobotovskyy et al. We removed the corresponding supplement and adapted the discussion as suggested.

-Many data are presented in the supplement. Upregulation of RGS5 by strech or NO as presented in Supplement X is important. These data are, however, only alluded to in the discussion section, and need to be moved to the results part of the manuscript.

Response
We agree with the reviewer and present the data of Supplement X as Figure 2 of the revised manuscript as suggested.

2nd Editorial Decision 14 May 2014

Thank you for the submission of your revised manuscript to EMBO Molecular Medicine. We have now received the enclosed reports from the referees that were asked to re-assess it. As you will see the reviewers are now globally supportive and I am pleased to inform you that we will be able to accept your manuscript pending editorial amendments.

Please submit your revised manuscript within two weeks. I look forward to seeing a revised form of your manuscript as soon as possible.

***** Reviewer's comments *****

Referee #1 (Remarks):

No further comments.

[Referees 2 and 3 had no additional comments and were satisfied].
